# Canopy Catalysts for Alkyne Metathesis: Investigations into a Bimolecular Decomposition Pathway and the Stability of the Podand Cap

**DOI:** 10.1002/chem.202102080

**Published:** 2021-08-26

**Authors:** Julius Hillenbrand, J. Nepomuk Korber, Markus Leutzsch, Nils Nöthling, Alois Fürstner

**Affiliations:** ^1^ Max-Planck-Institut für Kohlenforschung 45470 Mülheim an der RuhrMülheim/Ruhr Germany

**Keywords:** alkyne metathesis, metal-metal bonding, metal alkylidynes, molybdenum, tungsten

## Abstract

Molybdenum alkylidyne complexes with a trisilanolate podand ligand framework (“canopy catalysts”) are the arguably most selective catalysts for alkyne metathesis known to date. Among them, complex **1 a** endowed with a fence of lateral methyl substituents on the silicon linkers is the most reactive, although fairly high loadings are required in certain applications. It is now shown that this catalyst decomposes readily via a bimolecular pathway that engages the Mo≡CR entities in a stoichiometric triple‐bond metathesis event to furnish RC≡CR and the corresponding dinuclear complex, **8**, with a Mo≡Mo core. In addition to the regular analytical techniques, ^95^Mo NMR was used to confirm this unusual outcome. This rapid degradation mechanism is largely avoided by increasing the size of the peripheral substituents on silicon, without unduly compromising the activity of the resulting complexes. When chemically challenged, however, canopy catalysts can open the apparently somewhat strained tripodal ligand cages; this reorganization leads to the formation of cyclo‐tetrameric arrays composed of four metal alkylidyne units linked together via one silanol arm of the ligand backbone. The analogous tungsten alkylidyne complex **6**, endowed with a tripodal tris‐alkoxide (rather than siloxide) ligand framework, is even more susceptible to such a controlled and reversible cyclo‐oligomerization. The structures of the resulting giant macrocyclic ensembles were established by single‐crystal X‐ray diffraction.

## Graphical abstract

**Vulnerable**? The activity and functional group tolerance of metal alkylidyne canopy catalysts for alkyne metathesis is excellent, but their stability leaves room for optimization. The search for the Achilles′ heels unveiled a new decomposition pathway through bimolecular coupling and led to the discovery of a facile cyclo‐tetramerization reaction by (reversible) opening of the tripodal ligand cage.

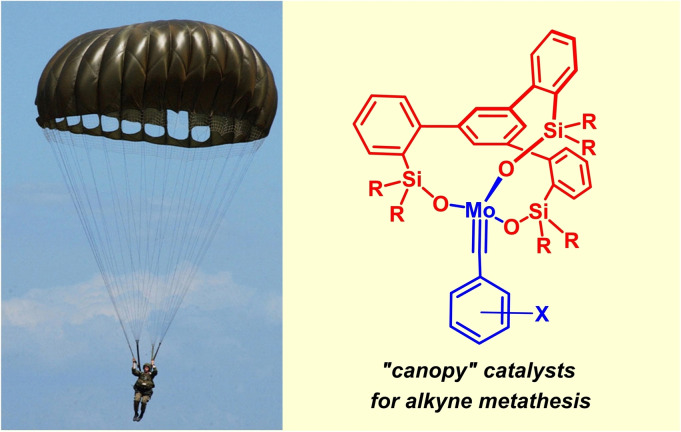

## Introduction

**Scheme 1:**
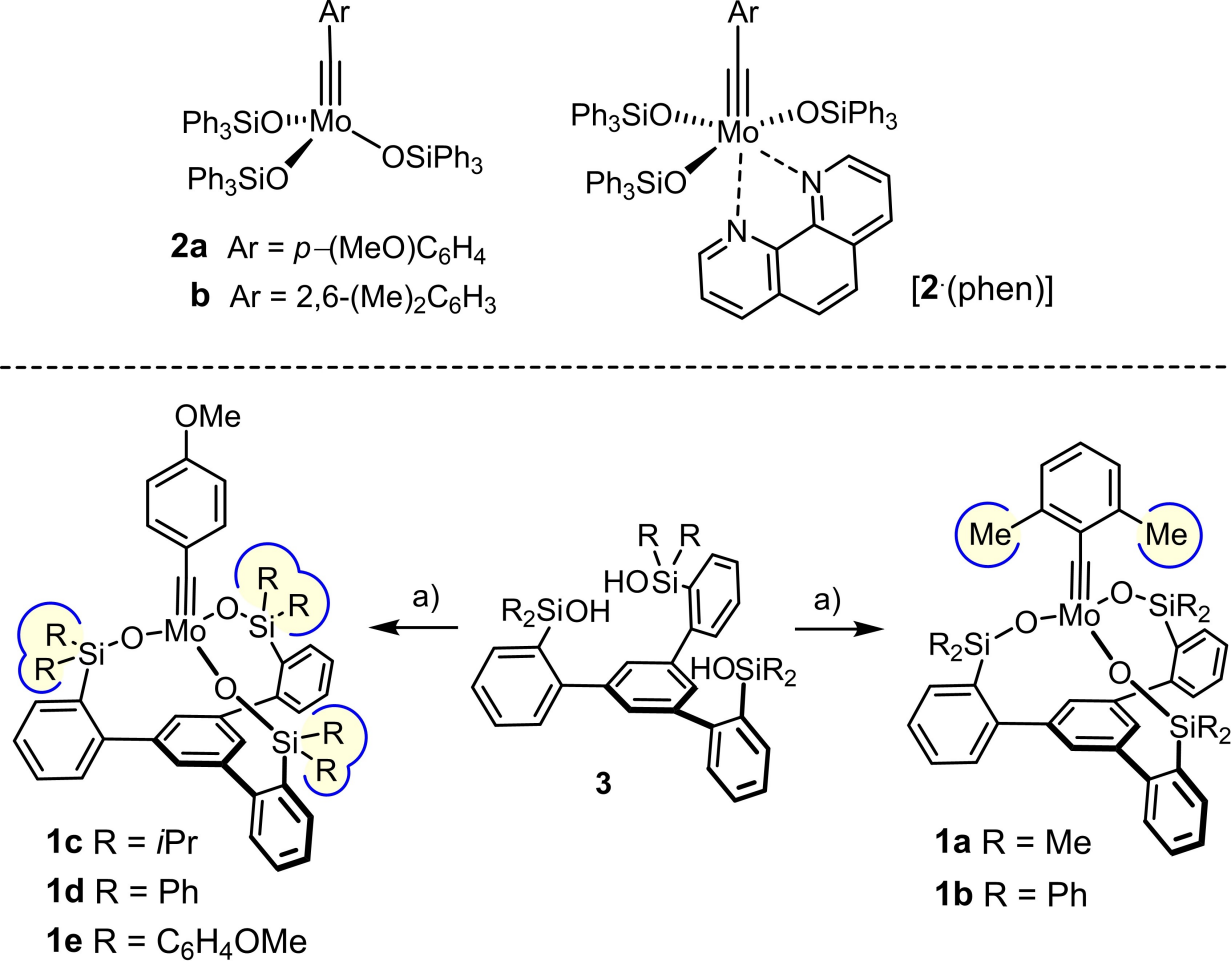
a) ArC≡Mo(O*t*Bu)_3_ (**4**), toluene; Ar=2,6‐(Me)_2_C_6_H_3_‐ or *p*‐(MeO)C_6_H_4_‐.

The discovery that silanolate ligands synergize remarkably well with molybdenum alkylidynes marks an important step in the development of alkyne metathesis.[^1^, ^2^, ^3^, ^4^, ^5^, ^6^] Even the parent complexes of type **2** combine high activity with a previously unknown tolerance towards functional groups (Scheme 1);[^7^, ^8^, ^9^, ^10^, ^11^] therefore, these catalysts opened new vistas for material science[^12^, ^13^] and stood the test of natural product total synthesis in numerous demanding cases.[^14^, ^15^] Moreover, the derived phenanthroline adducts [**2** ⋅ (phen)] are bench‐stable and hence easy to handle, yet can be re‐converted into the catalytically active species on treatment with ZnCl_2_ or MnCl_2_.[^7^, ^8^]

We conjectured that further improvements might be reached by taking advantage of the chelate effect. Success was met with ligands of type **3**, which furnished molybdenum alkylidyne complexes **1** distinguished by a tripodal ligand framework.[^16^, ^17^, ^18^, ^19^, ^20^, ^21^] Despite a high‐valent early transition metal forming the active center, these catalysts proved compatible with numerous Lewis basic heteroatom donor sites in the substrates, including secondary and tertiary amines, hydroxylamines, thioethers, pyridine, quinoline, thiazole, thiophene, etc. At the same time, they allow alkynes endowed with unprotected alcohol substituents or other protic groups to be metathesized, which had previously been largely inconceivable; even a certain stability towards moisture was noticed, although there remains much room for improvement.[^17^, ^22^, ^23^] Combined spectroscopic, crystallographic and computational studies unveiled the unorthodox mechanism by which such “canopy catalysts” operate as a consequence of the geometric constraints imposed onto the reactive intermediates by the tripodal silanolate ligand sphere.[^24^, ^25^]

**Figure 1:**
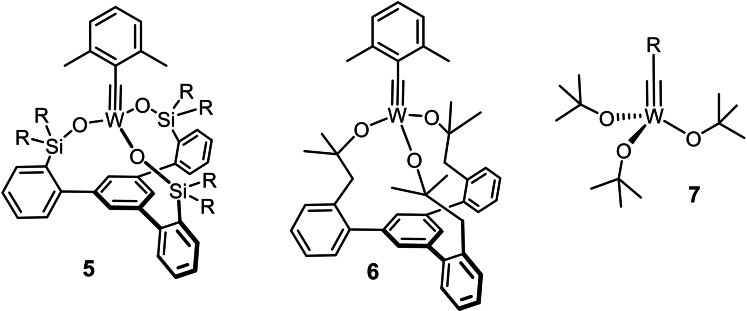
Tungsten alkylidynes with very different catalytic activity.

In view of these many virtues, it is perplexing that the remarkable synergy between silanolates and Mo(+6) does not find correspondence in the tungsten alkylidyne series (Figure 1). The Lewis acidity of the W(+6) center in **5** is upregulated to the extent that the catalytic turn‐over ceases.[^24^, ^25^, ^26^] Therefore, a fundamentally different ligand design had to be pursued:^[26]^ complex **6** with an expanded tris‐alkoxide (rather than siloxide) chelate ligand framework constitutes a promising new lead in that it outperformed a classical Schrock catalyst of type **7** (R=2,6‐dimethylphenyl),[^27^, ^28^, ^29^, ^30^] although it still does not rival the best molybdenum catalysts known to date.

While the basic features of the new catalyst families are fairly well understood,[^16^, ^17^, ^18^, ^24^, ^25^, ^26^] it is mandatory to refine the picture in order to lay a solid ground for further improvements. To this end, the following important aspects need to be addressed, which had only briefly been touched upon in our original disclosures: how stable are the podand ligand frameworks of **1** and **6**? Equally high on the agenda is a further interrogation of complex **1 a** as the arguably most performant catalyst of this series, which is superbly active and selective, yet often mandates rather high loadings for reasons that were not entirely clear. The investigation summarized below sought answers to these questions, which, in turn, provide valuable guidance for catalyst design.

## Results and Discussion

### Key strategic issues

**Scheme 2:**
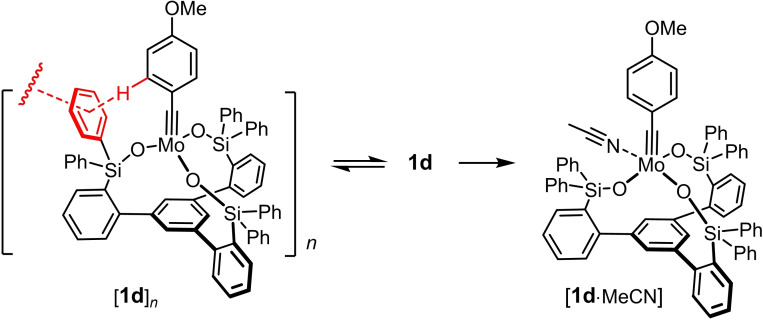
Canopy complexes such as **1 d** with a benzylidyne group and lateral aryl substituents on the silicon linkers form supramolecular dimers (oligomers) as a result of numerous intra‐ and intermolecular C−H/π‐ and π/π interactions shown schematically in red; these aggregates disassemble in solution upon gentle warming or upon coordination of MeCN.

So far, two subsets of “canopy catalysts” have been obtained:

Complexes endowed with an unhindered *p*‐methoxybenzylidyne unit mandate bulky substituents on the lateral silicon tethers; complexes **1 c**–**e** are representative.[^16^, ^17^] For R=aryl, the corresponding complexes actually form supramolecular aggregates in the solid state by virtue of innumerable C−H/π‐ and π/π interactions.^[31]^ These aggregates dissociate in solution upon gentle warming or upon coordination of a two‐electron donor ligand such as MeCN (Scheme 2).[^16^, ^17^]

Complexes comprising a more encumbered 2,6‐disubstituted benzylidyne group provide greater flexibility with regard to the substituents on silicon; supramolecular aggregation is not observed even for R=aryl.^[17]^ Smaller R groups tend to entail higher catalytic activity, because substrate binding and product de‐coordination are more facile; the relevant intermediates are neither (over)stabilized by dispersion interactions nor on electronic grounds.^[25]^

**Scheme 3:**
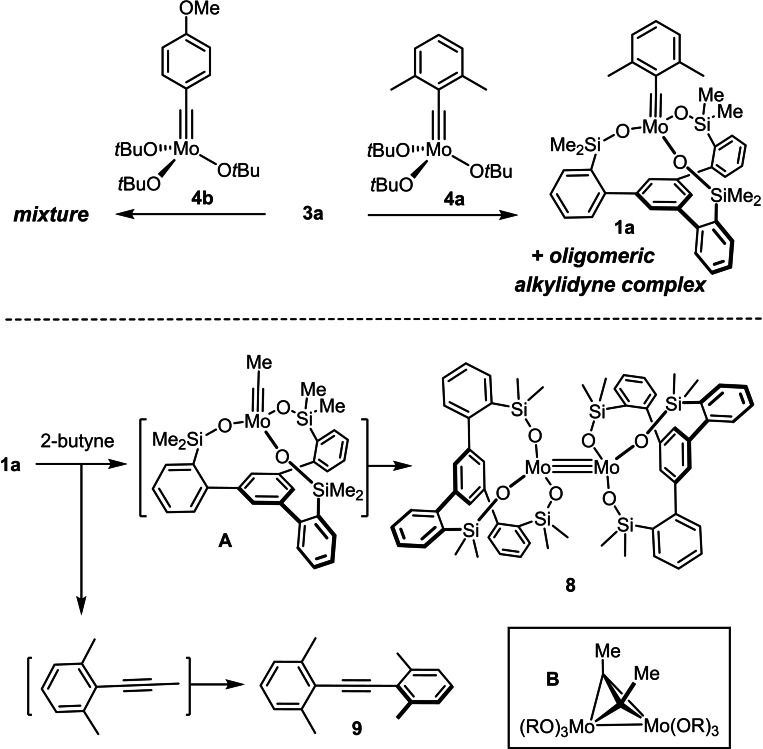
Formation and fate of catalyst **1 a**.

**Figure 2:**
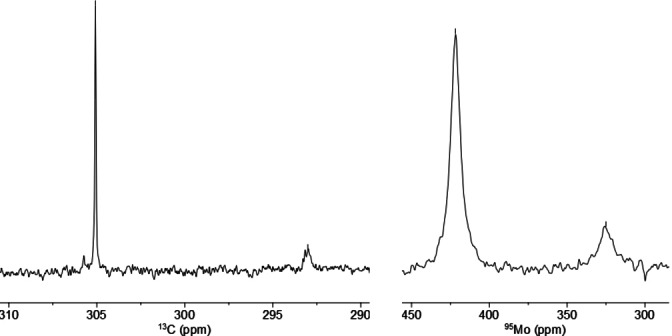
Relevant regions of the ^13^C NMR (left) and ^95^Mo NMR (right) spectra ([D_8_]toluene) of the product formed upon reaction of the trisilanol ligand **3 a** with the molybdenum alkylidyne **4 a**, comprising the expected canopy complex **1 a** and a second—likely oligomeric—alkylidyne complex.

Complex **1 a** as the currently most reactive catalyst of this class, however, seems to mark the limits. Upon formation from ligand **3 a** and precatalyst **4 a**, a second species is generated in small but non‐negligible quantity, which could neither be separated nor fully characterized (Scheme 3).^[17]^ The diagnostic spectral fingerprints (*δ*_C_=292.8 ppm; *δ*_Mo_=325.0 ppm) leave no doubt that this companion is an alkylidyne species too (Figure 2); the broad ^13^C NMR signal seems to indicate that a dimeric/oligomeric entity might have formed by covalent cross‐linking (rather than supramolecular assembly). Although the presence of this “impurity” does not distract from the excellent catalytic properties of **1 a**, it implies that oligomerization can compete with the formation of regular monomeric tripodal cage structures. In line with this notion, reaction of **3 a** with the slimmer alkylidyne precatalyst **4 b** furnished a complex mixture likely composed of various oligomeric species, which could not be characterized any further (Scheme 3).^[17]^

### Catalyst decomposition by bimolecular ligand coupling

The conclusion drawn from the experiments shown in Scheme 3 that the substitution pattern of the alkylidyne and the peripheral bulk of the ligand must be matching to ensure formation and structural integrity of a monomeric canopy complex is of utmost relevance for catalyst design. In this context, it is important to reiterate that the substituted benzylidyne of the starting complex gets replaced in the first turn of the catalytic cycle by a sterically much less demanding ethylidyne group, when working with methyl‐capped alkynes as the by far most common substrates for alkyne metathesis.[^2^, ^3^, ^4^, ^5^, ^6^] If the size of this operative substituent is linked to the stability of the ancillary ligand sphere, however, the initiation step might jeopardize the constitutional integrity of the active species. It is therefore necessary to investigate whether the podand ligand structure, once formed, persists during a catalytic reaction or not.

To probe this aspect, **1 a** as the most active catalyst of this series was exposed to but‐2‐yne (2 equiv.) in toluene at ambient temperature. NMR inspection showed quantitative formation of the tolane derivative **9** within ≈60 min reaction time.^[32]^ The signals of the starting complex decayed with the same rate, giving rise to a new species carrying the silanolate ligand, which was isolated in 65 % yield. Single crystals suitable for X‐ray diffraction could not be grown, but all available analytical and spectroscopic data speak for the homo‐bimetallic species **8**. Specifically, the signal of the former alkylidyne unit is missing but the ^1^H, ^13^C and ^29^Si NMR spectra show that the *C*_3_‐symmetric arrangement of the tripodal silanolate ligands of the starting material is preserved. The HRMS and combustion analysis data fitting to a molecular formula of C_60_H_66_Mo_2_O_6_Si_6_ are also consistent with the proposed dinuclear nature. Arguably most diagnostic, however, is the ^95^Mo NMR shift of **8** at *δ*_Mo_=2631.5 ppm, far off the molybdenum alkylidyne region (for comparison, **1 a** resonates at *δ*_Mo_=421.8 ppm)^[17]^ but very well in line with the data reported for [(*t*BuO)_3_Mo≡Mo(O*t*Bu)_3_] (*δ*_Mo_=2645 ppm)^[33]^ and [(MMPO)_3_Mo≡Mo(OMMP)_3_] (*δ*_Mo_=2632 ppm; MMPOH=1‐methoxy‐2‐methylpropanol).^[34]^

The formation of **8** from **1 a** constitutes a stoichiometric triple‐bond metathesis event and is therefore perhaps unsurprising from the fundamental point of view; documented examples of such a coupling reaction, however, are surprisingly rare and their significance remained basically unassessed.[^35^, ^36^] What had been isolated in the past was a dimetallatetrahedrane complex of type **B** as the presumed key intermediate of this type of transformation;[^8^, ^37^, ^38^] this species had been taken as an indication that collision of two metal alkylidynes might constitute a previously undescribed catalyst decomposition pathway.^[8]^ The present result confirms this interpretation and shows that the coupling process does not stop at this intermediate stage but goes to completion with release of a low‐valent triple‐bonded Mo(+3) dimer,^[39]^ if a small‐enough ligand set is chosen. Although other as yet unidentified molybdenum‐containing side products might be present in the crude mixture, bimolecular metathetic coupling of **1 a** is obviously facile in the presence of but‐2‐yne (i. e., under the conditions of a catalytic alkyne metathesis reaction) and constitutes the major decomposition pathway. This observation is thought to explain why some applications of this catalyst required high loadings, especially in cases in which elevated temperature was necessary, because productive metathesis and catalyst decomposition might proceed with similar rates.[^17^, ^40^] This conclusion also implies that a reasonable catalyst lifetime is contingent upon efficient scavenging of but‐2‐yne, which is commonly achieved with the aid of molecular sieves following an earlier suggestion of our group.[^7^, ^8^, ^41^, ^42^]

### Ligand tuning

It is important to note that this bimolecular degradation in the presence of but‐2‐yne is particularly facile for complex **1 a** as the sterically least encumbered complex of this series. This structural attribute is also one of the reasons why this catalyst is so reactive, as the small peripheral “fence” does neither impede substrate binding nor product decoordination.^[25]^ Complex **1 b** with phenyl groups on silicon does not decompose along this pathway but is notably less active.

**Scheme 4:**
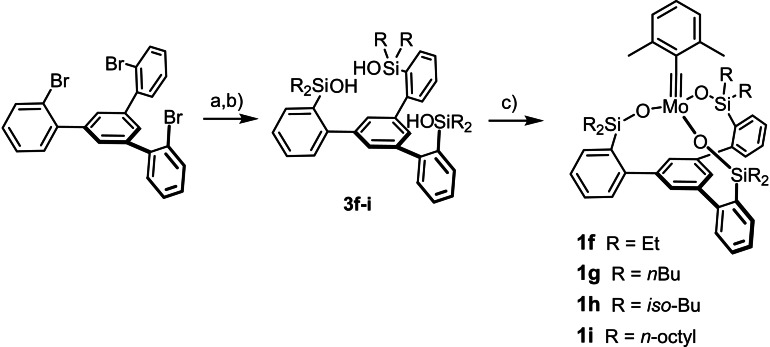
a) i) *t*BuLi, Et_2_O, −125 °C; ii) R_2_SiXH, −125 °C to RT, 91 % (R=Et), 89 % (R=*n*Bu), 77 % (R=*iso*‐Bu), 51 % (R=*n*‐octyl); b) *m*CPBA, CH_2_Cl_2_, 0 °C to RT, 99 % (**3 f**, R=Et), 71 % (**3 g**, R=*n*Bu), 97 % (**3 h**, R=*iso*‐Bu), 86 % (**3 i**, R=*n*‐octyl); c) **4 a**, toluene, 99 % (**1 f**), 58 % (**1 g**), 86 % (**1 h**) 30 % (**1 i**); *m*CBPA=*meta*‐chloroperbenzoic acid.

**Figure 3:**
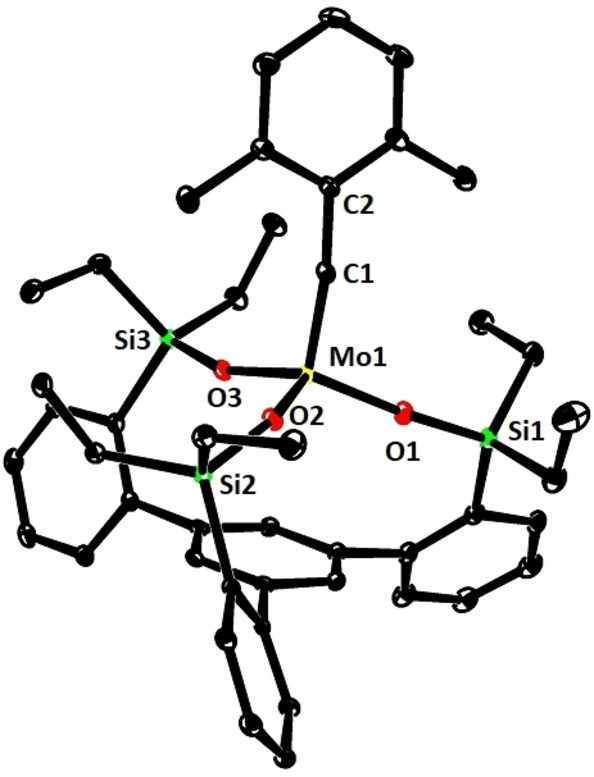
Structure of complex **1 f** in the solid state.

It seemed worthwhile to investigate whether yet other substitution patterns lead to a better balance between catalytic performance and stability. Although a certain loss of activity seems inevitable when going away from R=Me, small and unbranched alkyl groups might represent a reasonable compromise. To this end, complexes **1 f**–**i** were prepared by adapting the established route; in no case was the formation of oligomeric side‐products observed during the actual complexation step (Scheme 4). As expected, **1 f**–**i** are monomeric species in solution and in the solid state (Figure 3).^[43]^ The fact that the recorded ^95^Mo NMR chemical shifts (**1 f**: 417 ppm; **1 g**: 420 ppm; **1 h**: 433 ppm; **1 i**: 420 ppm) are almost identical with that of **1 a** (*δ*_Mo_=422 ppm) is taken as an indication that the electronic character remains largely unaffected by the replacement of the methyl groups by higher alkyl substituents on the silicon linkers. Any change in activity and stability is hence (essentially) steric in origin.

**Figure 4:**
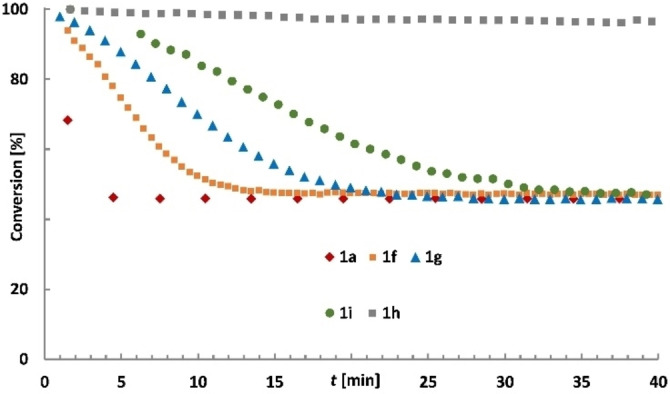
Benchmarking of the catalytic activity of the new complexes: the consumption of 1‐methoxy‐4‐(prop‐1‐yn‐1‐yl)benzene was monitored by ^1^H NMR ([D_8_]‐toluene, 27 °C, 5 mol% catalyst loading).

The homo‐metathesis of 1‐methoxy‐4‐(prop‐1‐yn‐1‐yl)benzene with formation of 1,2‐bis(4‐methoxyphenyl)ethyne was chosen to benchmark their activity (Figure 4). The reaction was deliberately carried out in the absence of molecular sieves to preclude any interference with the innate reactivity of the different complexes. As expected, **1 a** is unrivaled in that it leads to the equilibrium composition in ≤5 min at 27 °C. Gratifyingly, however, the ethyl‐ (**1 f**) and the *n*‐butyl variants (**1 g**) also show high activity (15–20 min), and even the *n*‐octyl derivative (**1 i**) results in fast conversion (35 min). In contrast, complex **1 h** carrying *iso*‐butyl residues on silicon is hardly active at (near) ambient temperature.

In terms of stability, however, the larger substituents pay valuable dividends: even the ethyl derivative **1 f** did not decompose by bimolecular coupling of the alkylidyne unit in the presence of but‐2‐yne to any notable extent; a solution in [D_8_]toluene was stable when kept overnight, as judged by NMR. Moreover, we note that the more greasy environment crafted about the molybdenum center by the larger alkyl groups entails improved stability in air: specifically, complex **1 g** has a half‐lifetime on the bench on the order of 24 h. Although long‐term storage under inert atmosphere remains necessary, the complex can be weighed and transferred in air, which is deemed an important step towards a more user‐friendly system. Although **1 a** likely remains the catalyst of choice, especially when it comes to metathesizing substrates which themselves are sterically demanding, the otherwise favorable attributes of the higher alkyl‐homologues **1 f**–**i** warrant further study. A more comprehensive investigation into their activity, stability and fate is currently underway.

### Opening of the tripodal cage: Case study of a defined cyclo‐oligomerization in the molybdenum series

As has already been pointed out above, partial cross‐linking accompanies the preparation of **1 a** (Scheme 3, Figure 2). It is hence important to investigate whether or not this side reaction can also occur by re‐opening of an intact ligand cage once a monomeric complex of type **1** has been formed.

**Scheme 5:**
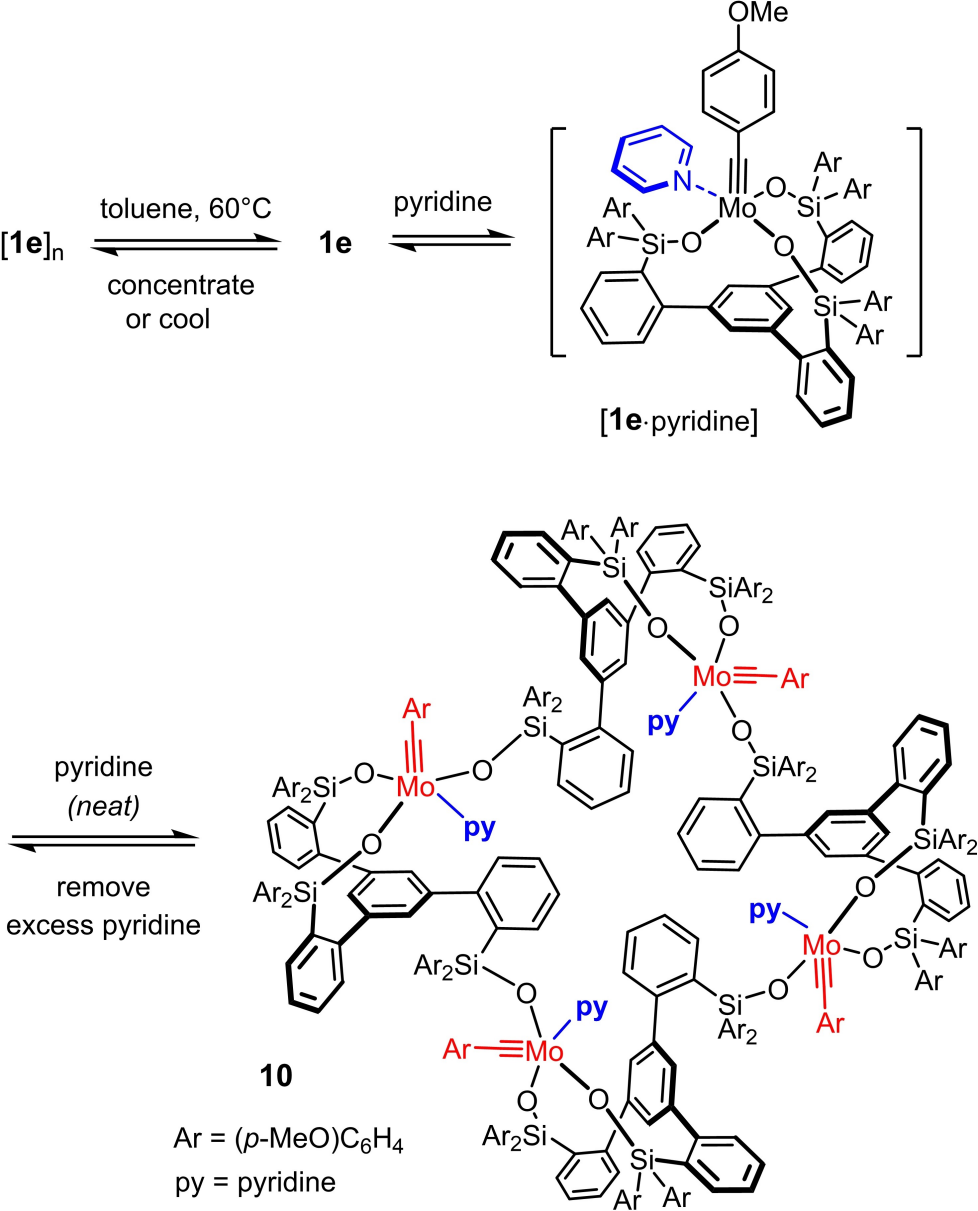
Formation of a cyclo‐tetrameric array.

**Figure 5:**
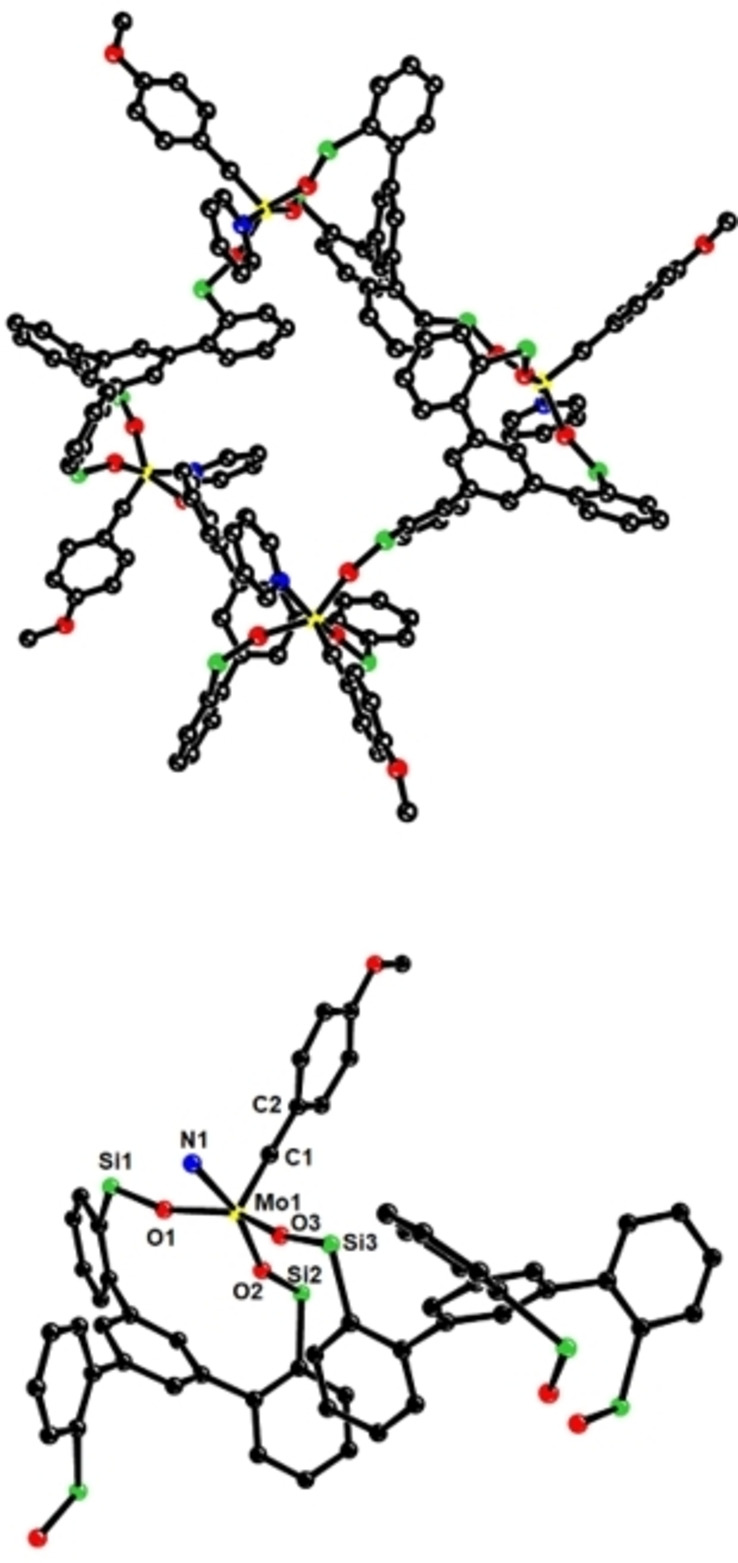
Top: Truncated view of the cyclo‐tetramer **10** containing four molybdenum benzylidyne units and four coordinated pyridine ligands (the two additional phenyl groups at each silicon atom as well as all hydrogen atoms are omitted for the sake of clarity). Bottom: one of the four alkylidyne units in the cyclo‐tetramer with a partly opened ligand framework; the full structure and additional crystallographic information are found in the Supporting Information. Color code: C=black, Mo=yellow, N=blue, O=red, Si=green; averaged values of selected bond lengths [Å] and angles [°]: Mo1−C1 1.746(7), Mo1−N1 2.271(6), Mo1−O1 1.935(5), Mo1−O2 1.931(4), Mo1−O3 1.924(4), Mo1−C1−C2 177.5(66), Mo1−O1−Si1 162.3(3), Mo1−O2−Si2 152.7(3), Mo1−O3−Si3 158.6(3).

At the present stage of development, we have no indications that partial or total cleavage of the tripodal framework is a major concern during a catalytic alkyne metathesis reaction, even though a single piece of evidence shows that the ligand architecture is indeed vulnerable. In an attempt to break supramolecular aggregation, a solution of the yellow *p*‐methoxybenzylidyne complex [**1 e**]_*n*_ in [D_8_]toluene was treated with pyridine (1–4 equiv.) to give what appears to be the expected adduct [**1 e** ⋅ pyridine] (Scheme 5). At low temperature, the spectra are sufficiently well resolved to assign this structure with confidence (see the Supporting Information). When [**1 e**]_*n*_ was dissolved in neat pyridine, however, a deep purple solution was formed that did not contain the expected monomeric pyridine adduct; rather, selective cyclo‐oligomerization with formation of the giant ring system **10** had occurred. Although this product is molecularly well defined, the NMR signals are broad and rather featureless. Gratifyingly though, purple crystals suitable for X‐ray diffraction could be grown by layering the pyridine solution with pentane, which gave a definitive answer as to the constitution of this complex (Figure 5).^[43]^

**Figure 6:**
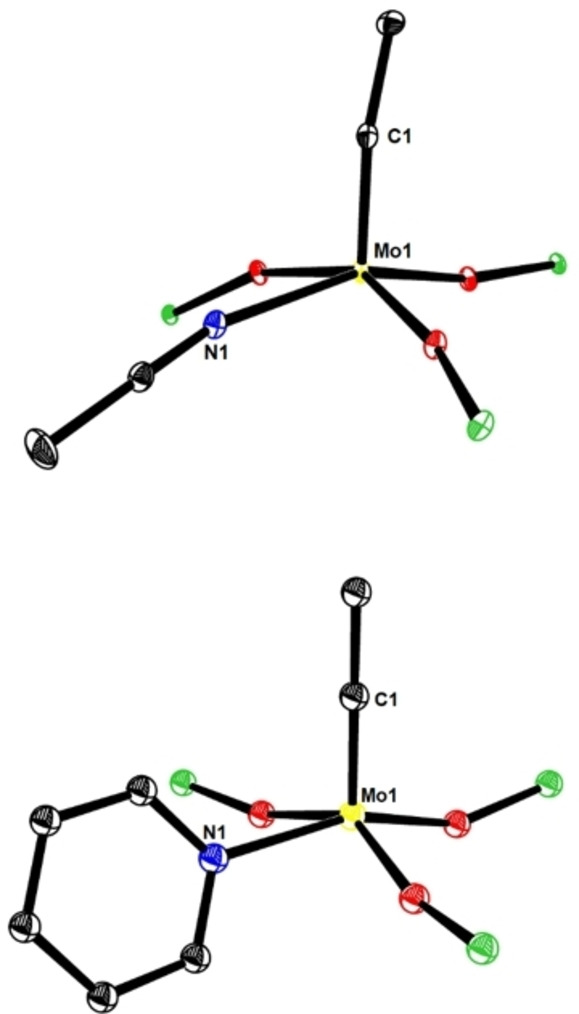
The inner cores of the canopy complex [**1 d** ⋅ MeCN] (top) and one of the four alkylidyne units comprised in **10** (bottom) have notably different curvatures. Dihedral angles C1−Mo1−O−Si: 142.8, 115.6, 11.9 ([**1 d** ⋅ MeCN]); 12.4, 10.3, 15.5, 14.2, 10.9, 15.5 (**10**).

Complex **10** incorporates four molecules of former **1 e** joined together via one silanolate arm of each constituent to form a macrocyclic array comprising four intact alkylidyne entities; each molybdenum center carries an additional pyridine ligand. The metal atoms formally reside on a bent rectangle and are almost equidistant from each other (11.09–11.12 Å). Whereas the bond lengths and angles of the individual alkylidyne units all fall into the normal ranges,[^7^, ^8^, ^16^, ^17^] the curvature of the silanolate umbrella is noteworthy: specifically, the C1−Mo1−O−Si dihedral angles are much smaller than those of a typical canopy catalyst. Figure 6 illustrates this aspect by comparison of the inner cores of one of the four alkylidyne groups contained in **10** with that of [**1 d** ⋅ MeCN], which was chosen as the reference point because it also carries an external nitrogen‐based donor ligand.^[16]^ As viewed from the central metal, the Si atoms point “upward” in **10**, resulting in a “concave” ligand environment; in the canopy catalyst [**1 d** ⋅ MeCN], in contrast, they are oriented “downward”, away from the molybdenum center, to form a “convex” ligand architecture. In this conformational regard, the individual alkylidyne units forming cyclo‐tetramer **10** resemble the parent complexes [Ph_3_SiO)_3_Mo≡CAr] (**2**) carrying totally unconfined monodentate ligands.[^7^, ^8^] The transformation of [**1 e**]_*n*_ into the cyclo‐tetrameric adduct **10** hence comes along with a significant geometric change, which likely indicates strain relief; it is reasonable to assume that the resulting enthalpic gain constitutes a major driving force (even though ligation of the metal center to pyridine may also play a role, see below). The reasons as to why this particular cyclotetrameric adduct is generated, however, are not clear at this point.

Parenthetically we note that the structure of **10** in the solid state is also remarkable from the purely crystallographic viewpoint because four of these giant cyclo‐tetrameric rings are contained in the unit cell, which gains a volume of no less than 35098(3) Å^3^ (for a discussion, see the Supporting Information). The elemental analysis as well as the recorded HRMS data are consistent with the assigned structure: the fact that the mass of the tetramer without the four coordinated pyridines was detected by MS indicates that these heterocyclic donor ligands come off the high‐valent molybdenum centers without instant breakdown of the cyclo‐oligomeric frame (for details, see the Supporting Information); this observation, in turn, suggests that even adduct **10** likely retains some catalytic activity.^[44]^ When the excess pyridine was pumped off in high vacuum, however, the color of the sample changed back to yellow and the NMR spectrum of the residue in [D_8_]toluene at elevated temperature showed **1 e** as the only detectable species. This particular case hence provides compelling evidence that opening of the canopy ligand framework, as present in complexes of type **1**, is possible but potentially reversible.

### Concentration‐dependent cyclo‐tetramerization of the tungsten catalyst 6

**Scheme 6:**
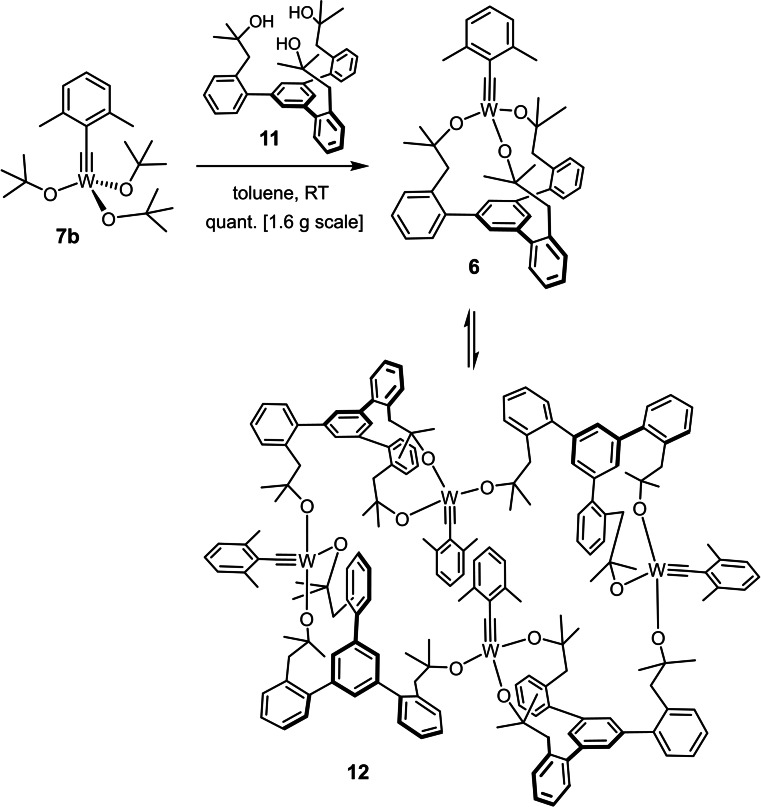
Preparation of the tungsten complex **6** and concentration‐dependent equilibrium with the cyclo‐tetrameric array **12**.

NMR spectra recorded in [D_8_]‐THF suggested that the crude product formed on treatment of the tungsten complex **7 b** with ligand **11** contains only the monomeric tungsten alkylidyne **6**, which is easily identified by virtue of the (approximately) *C*_3_‐symmetric coordination sphere.^[26]^ When the same crude material was dissolved in [D_8_]‐toluene, however, signals of a second alkylidyne complex were detected, the amount of which was found to be concentration‐dependent. Under high‐dilution conditions, as commonly applied to ring closing alkyne metathesis (RCAM) reactions for the formation of macrocycles, only the monomeric species **6** was detected, whereas the signals of the second complex of lower symmetry gained intensity when the concentration was increased; the process is reversible (Scheme 6). DOSY NMR data showed that the two entities are of considerably different size/molecular weight (*D*_exp_=6.62×10^−10^ vs. 9.02×10^−10^ m^2^ ⋅ s^−1^); both of them are metal alkylidynes as deduced from the characteristic ^13^C and ^183^W NMR shifts in [D_8_]toluene (*δ*_C_=264 and 260 ppm; *δ*_W_=114 and 27 ppm, respectively).^[26]^

Equilibration of **6** with a dimeric or (cyclo)oligomeric species was the most plausible explanation for this observation put forward in the original publication,^[26]^ but an unambiguous conformation was difficult to attain because the concentration‐dependent composition of the mixture rendered all attempts at crystallizing the individual constituents challenging. After many failed attempts, tiny single crystals could be grown at −20 °C from concentrated solutions in fluorobenzene; for their very small size, however, the intensity of a synchrotron beam was necessary to record suitable diffraction data.

**Figure 7:**
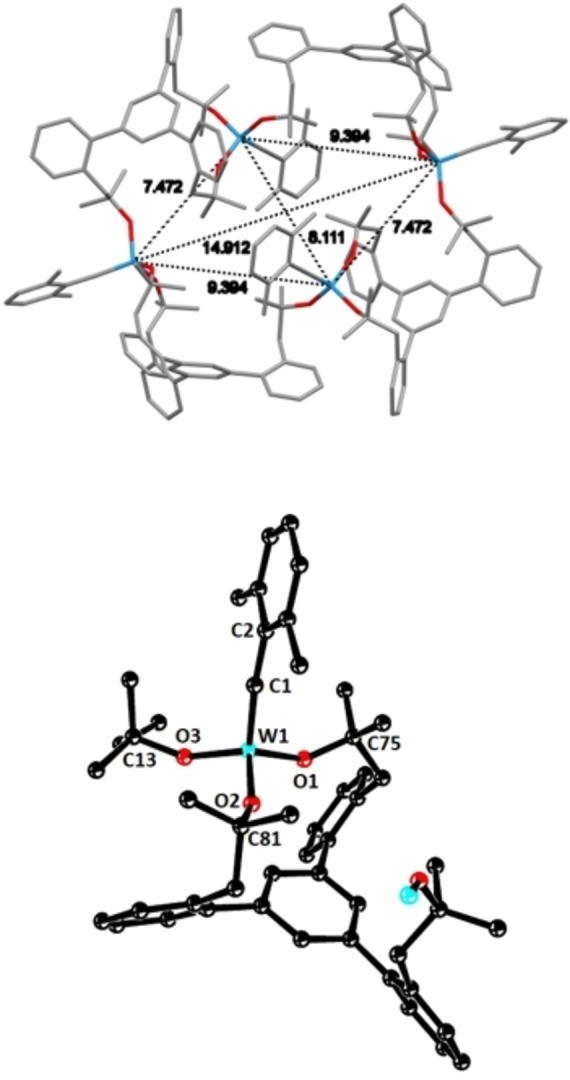
Top: Structure of the cyclo‐tetrameric tungsten alkylidyne complex **12** in the solid state; a capped‐sticks presentation is chosen and H‐atoms are omitted for clarity. Bottom: One of the four constituent tungsten alkylidyne units, showing the partly opened chelate ligand sphere; for the full structure and additional crystallographic information, see the Supporting Information. Color code: W=cyan, O=red; averaged values of selected bond lengths [Å] and dihedral angles [°]: W1‐C1 1.778(14), W1−O1 1.867(8), W1−O2 1.866(8), W1−O3 1.872(8), C1−W1−O1−C75 17.4(14), C1−W1−O2−C81 20.5(15), WC1−W1−O3−C13 40.5(19).

Figure 7 shows the cyclo‐tetrameric array of **12** in the solid state and also zooms in on one of the subunits.^[43]^ In contrast to the molybdenum case described above, all four metal atoms reside on the same plane to form a virtual parallelogram. With 9691 Å^3^, the unit cell is much smaller than that of **10**, because it contains only a single such tetrameric entity. The C≡W bond lengths fall into the normal range, as do the bond angles of the W−O−C units (for details, see the Supporting Information).^[29]^ As no crystal structure of the monomeric precursor complex **6** is available, a detailed comparison with the derived cyclo‐tetramer **12** is currently not possible. Yet, we note that all four tethered tungsten alkylidyne entities appear largely unstrained and undistorted, featuring a relaxed “concave” shape of the first coordination sphere (as manifested in the invariably small dihedral angles C1−W1−O−C), which the classical Schrock alkylidyne complex [(*t*BuO)_3_W≡CCMe_3_] (**7**) is also known to adopt.^[45]^ Therefore, and in analogy to the molybdenum case discussed above, strain release is again believed to be accountable, at least in part, for the formation of this remarkable ensemble.

## Conclusions

An earlier study has already shown that reducing the size of the substituents on the silicon linkers leads to a notable increase in the catalytic activity of molybdenum alkylidyne complexes endowed with a podand trisilanol ligand structure at the expense of their lifetime.^[17]^ In line with this notion, the “canopy” complex **1 a** endowed with a “fence” of lateral methyl groups represents the currently best performing member of this series, but sometimes requires fairly high loadings. It has now been found that this particular catalyst degrades via a peculiar bimolecular pathway, which results in the formation of the dinuclear Mo(+3) complex **8** comprising a Mo≡Mo bond and the substituted alkyne derived from the organic fragment of the former alkylidyne unit. Although such a stoichiometric triple‐bond metathesis event is perfectly feasible, it has surprisingly little precedent in the literature; in fact, this case seems to be the first example for which the significance has been recognized and discussed. This finding is arguably of relevance for future attempts at finding an optimal balance between the activity and stability of alkyne metathesis catalysts in general and canopy catalysts in particular. The preparation of the higher homologues **1 f**–**i** represents a first step in this direction.

It is equally relevant to note that the more encumbered siblings of type **1** with bulkier silyl substituents are (much) more resistant to this decomposition process, but can succumb to cyclo‐oligomerization by opening of the tripodal ligand architecture. Crosslinking will ensue, as manifested in the formation of the cyclo‐tetrameric entity **10** upon dissolution of complex [**1 e**]_*n*_ with neat pyridine. A related process was observed for the tungsten alkylidyne **6** carrying a tripodal trisalkoxide (rather than siloxide) ligand, which responds to changes of the concentration in toluene solution by facile and apparently reversible cyclo‐tetramerization with formation of complex **12**. Based on crystallographic data, it is proposed that the driving force for these surprisingly selective cyclo‐oligomerization reactions stems, at least in part, from the relaxation of the ligand sphere upon (partial) opening of the obviously somewhat strained tripodal cages. This information is also relevant for ongoing catalyst development exercises, which must attempt at designing any strain out of the ligand sphere while maintaining the advantages rooted in the chelate effect.

## Conflict of interest

The authors declare no conflict of interest.

## Supporting information

As a service to our authors and readers, this journal provides supporting information supplied by the authors. Such materials are peer reviewed and may be re‐organized for online delivery, but are not copy‐edited or typeset. Technical support issues arising from supporting information (other than missing files) should be addressed to the authors.

Supporting Information
