# Supplementary material for: Canopy Catalysts for Alkyne Metathesis: Investigations into a Bimolecular Decomposition Pathway and the Stability of the Podand Cap
Source: Chemistry. 2021 Aug 26;27(56):14025–33. doi: 10.1002/chem.202102080 (PMC8518412; doi:10.1002/chem.202102080)
Supplement: Supplementary file 1 — Supporting Information [file CHEM-27-14025-s001.pdf]

Chemistry–A European Journal

Supporting Information

Canopy Catalysts for Alkyne Metathesis: Investigations into a Bimolecular Decomposition Pathway and the Stability of the Podand Cap

Julius Hillenbrand, J. Nepomuk Korber, Markus Leutzsch, Nils Nöthling, and Alois Fürstner*

Supporting Crystallographic Information

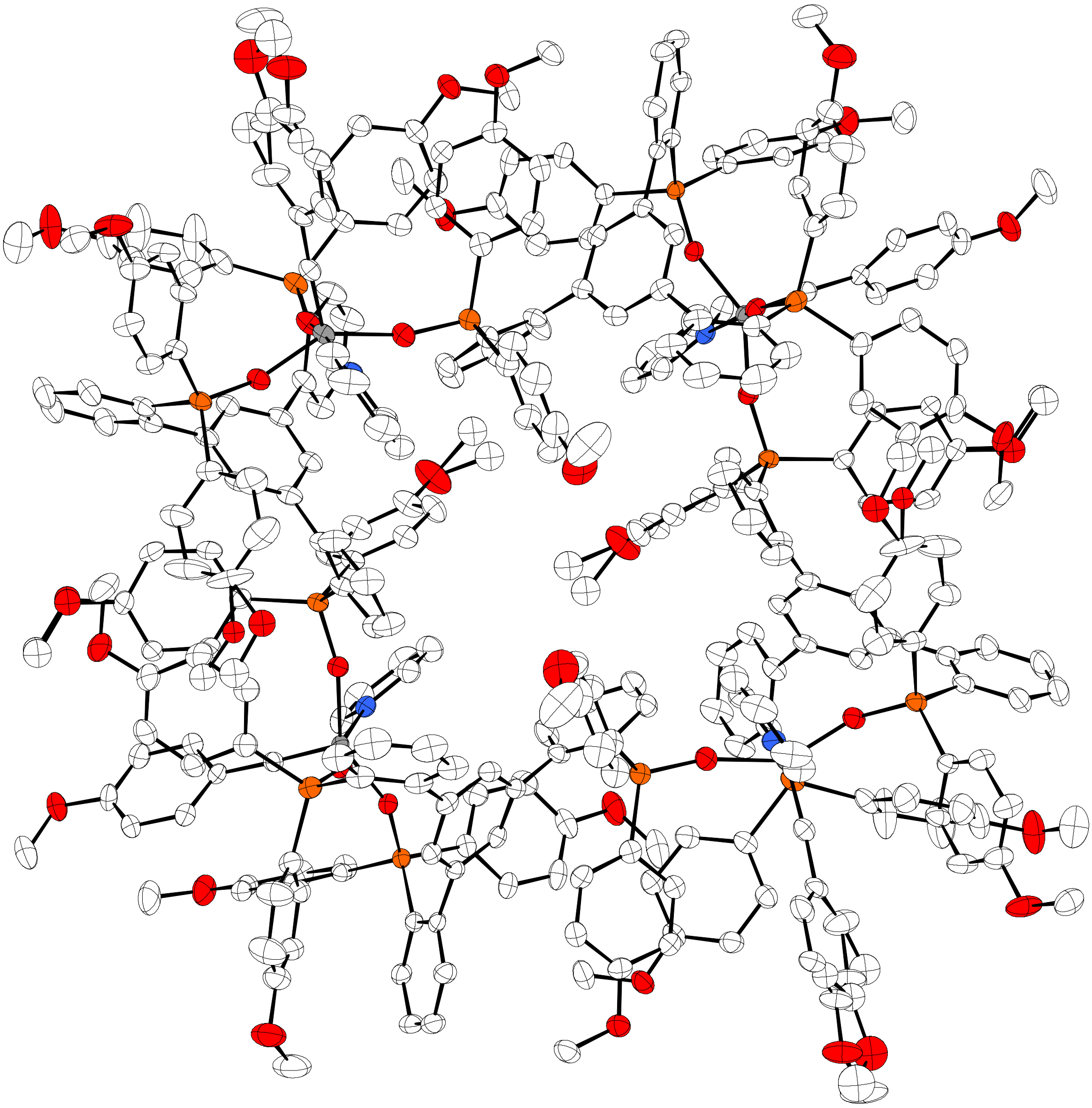

Figure S1. Plot of the molecular structure of tetrameric pyridine adduct **10**. Atomic displacement ellipsoids shown at the 50 % probability level; H atoms are omitted for clarity. Color code: Mo = grey, O = red, N = blue, Si = orange.

X-ray Crystal Structure Analysis of the Tetrameric Pyridine Adduct 10: $C_{316}H_{276}Mo_4N_4O_{40}Si_{12}$, M_r = 5490.23 g · mol^{-1}, violet prism, crystal size 0.257 x 0.105 x 0.100 mm^3, monoclinic, space group $C2/c$ [15], a = 25.6416(12) Å, b = 29.0265(13) Å, c = 47.213(2) Å, β = 92.816(2)°, V = 35098(3) Å^3, T = 100(2) K, Z = 4, D_{calc} = 1.039 g · cm^3, λ = 0.71073 Å, $\mu(Mo\text{-}K_\alpha)$ = 0.238 mm^{-1}, analytical absorption correction (T_{min} = 0.96, T_{max} = 0.98), Bruker-AXS Kappa Mach3 diffractometer with APEX-II detector and IμS micro focus X-ray source, 0.864 < θ < 26.287°, 575157 measured reflections, 35471 independent reflections, 28987 reflections with $I > 2\sigma(I)$, R_{int} = 0.0715, 1726 parameters, S = 1.072, residual electron density

+1.2 (0.94 Å from Mo2) / -0.9 (0.65 Å from Mo2) e · Å^{-3}. The structure was solved by *SHELXT* and refined by full-matrix least-squares (*SHELXL*) against F^2 to R_1 = 0.061 [$I > 2\sigma(I)$], wR_2 = 0.147. **CCDC-1987918**

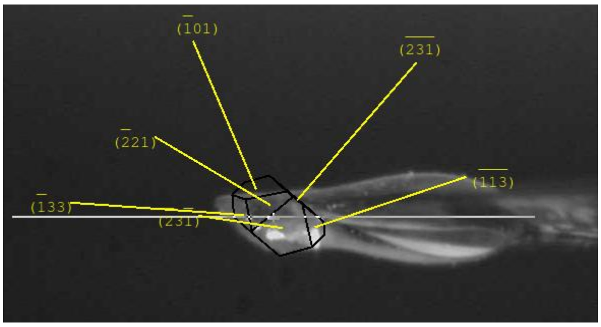

H	K	L	Distance [mm]
-2	3	-1	0.060
2	-3	1	0.060
-1	-1	-3	0.090
1	1	3	0.070
2	-2	-1	0.050
-2	2	1	0.050
1	-3	-3	0.060
-1	3	3	0.050
1	0	-1	0.040
-1	0	1	0.050
2	3	1	0.040
-2	-3	-1	0.050

INTENSITY STATISTICS FOR DATASET

Resolution	#Data	#Theory	%Complete	Redundancy	Mean I	Mean I/s	Rmerge	Rsigma
Inf - 3.27	546	566	96.5	16.82	58.87	80.34	0.0250	0.0098
3.27 - 2.19	1283	1283	100.0	27.17	22.51	75.39	0.0325	0.0092
2.19 - 1.74	1795	1795	100.0	28.96	15.76	67.45	0.0406	0.0103
1.74 - 1.51	1896	1897	99.9	28.47	11.16	56.46	0.0505	0.0124
1.51 - 1.37	1857	1857	100.0	25.66	9.01	44.99	0.0586	0.0155
1.37 - 1.27	1876	1876	100.0	20.97	7.84	35.38	0.0683	0.0199
1.27 - 1.20	1693	1693	100.0	18.60	6.36	28.14	0.0810	0.0253
1.20 - 1.14	1793	1793	100.0	16.85	5.45	23.55	0.0931	0.0310
1.14 - 1.09	1844	1844	100.0	15.63	4.77	20.11	0.1071	0.0367
1.09 - 1.05	1715	1715	100.0	14.83	4.50	18.28	0.1137	0.0407
1.05 - 1.01	1987	1987	100.0	14.27	3.91	15.83	0.1280	0.0474
1.01 - 0.98	1725	1725	100.0	13.79	3.33	13.67	0.1502	0.0567
0.98 - 0.95	1952	1952	100.0	13.39	3.04	12.39	0.1641	0.0639
0.95 - 0.92	2162	2162	100.0	12.69	2.63	10.59	0.1867	0.0762
0.92 - 0.90	1675	1675	100.0	11.76	2.36	9.14	0.2074	0.0893
0.90 - 0.88	1727	1727	100.0	10.94	2.08	7.80	0.2306	0.1049
0.88 - 0.86	2008	2008	100.0	10.51	2.08	7.43	0.2422	0.1108
0.86 - 0.84	2114	2114	100.0	9.98	2.05	6.86	0.2448	0.1192
0.84 - 0.83	1170	1170	100.0	9.89	1.90	6.36	0.2621	0.1306
0.83 - 0.81	2460	2460	100.0	9.59	1.77	5.84	0.2823	0.1452
0.81 - 0.80	928	992	93.5	6.71	1.60	4.58	0.3036	0.2022
0.90 - 0.80	10407	10471	99.4	9.83	1.94	6.62	0.2544	0.1279
Inf - 0.80	36206	36291	99.8	16.03	6.30	23.84	0.0688	0.0321

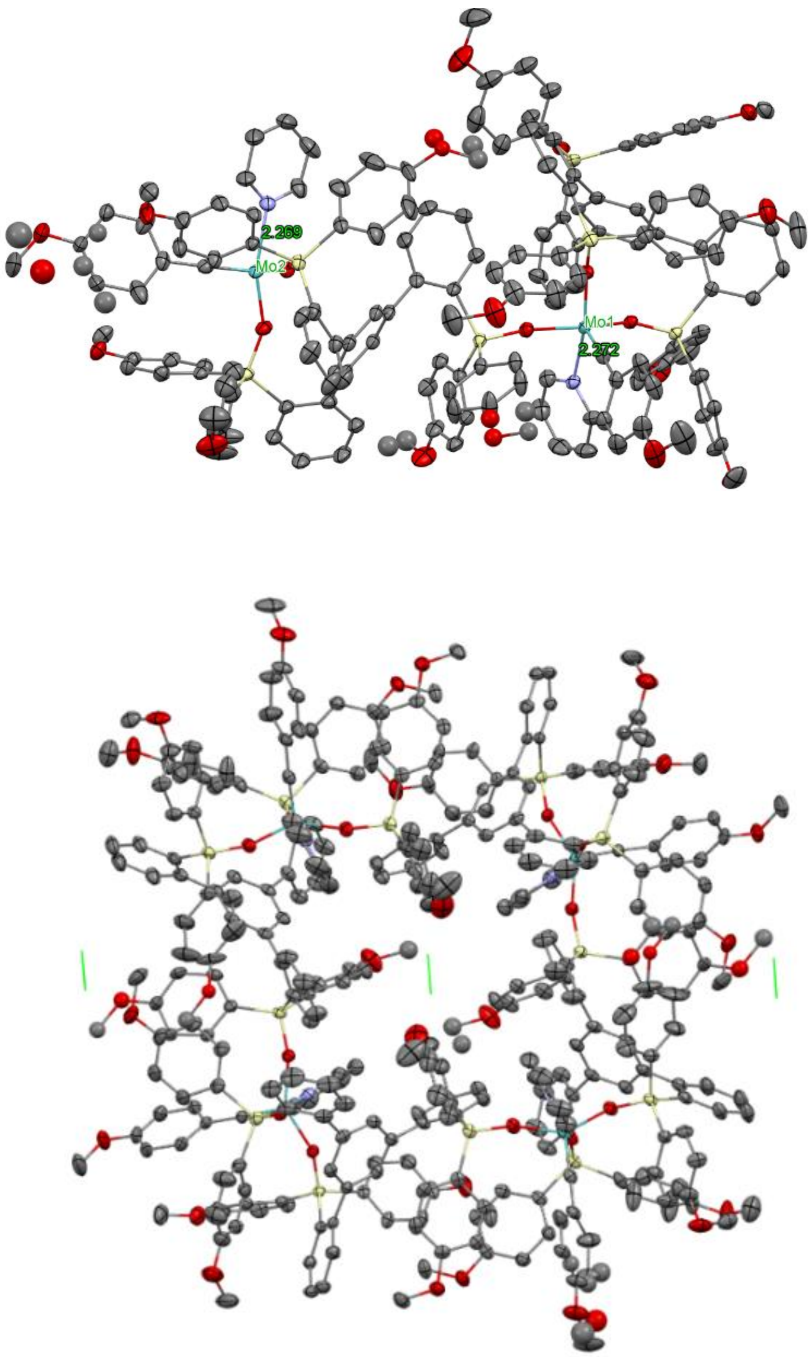

Figure S2. Asymmetric unit of **10** showing coordination of pyridine to the metallic centre (top) and tetrameric structure of **10** (bottom) about the twofold crystallographic axis at the centre of gravity.

The tetrameric molecule forms an untypically large entity with four molecules in the unit cell dimensions of a = 25.6416(12) Å, b = 29.0265(13) Å, c = 47.213(2) Å and V = 35098(3) Å^3. There is evidence of several non-interacting pyridine molecules and possibly disordered pentane present in the structure. The relatively poor crystal quality is reflected in the diffraction data, which do not allow an exact description of the disordered solute. To improve signal-to-noise ratio quality the SQUEEZE routine in PLATON was applied to dataset.[1] This results in a residual electron density of 1.178 and -0.911 eÅ^{-3} after final refinement.

---

1 A. L. Spek, *Acta Cryst.* **2015**, C71, 9-18.

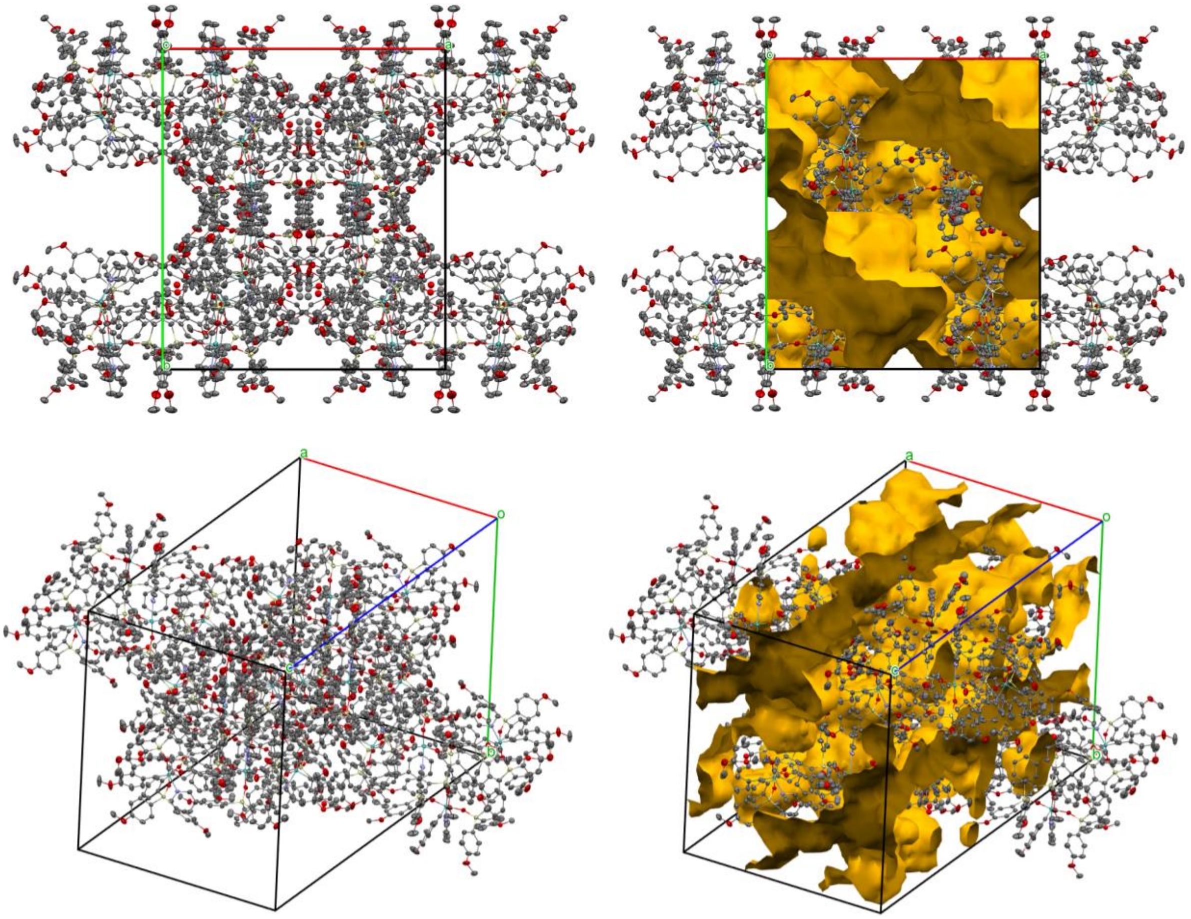

Figure S3. Packing of compound **10** in the unit cell. View along the *c* axis (top left) and in a random orientation (bottom left). Calculated voids (probe radius 1.2 Å, 0.7 Å grid spacing) are shown respectively on the right.

After application of the SQUEEZE routine, voids remain in the structure, corresponding to 27.2% (9554.91 Å^3) empty space in a total unit cell volume of 35098(3) Å^3. Eight low index diffraction intensities were obstructed by the beam stop and omitted from final refinement. In addition, the terminal groups of several coordinating *p*-methoxybenzylidyne ligands are partially disordered (70:30 and 50:50). H atoms were refined using a riding model.

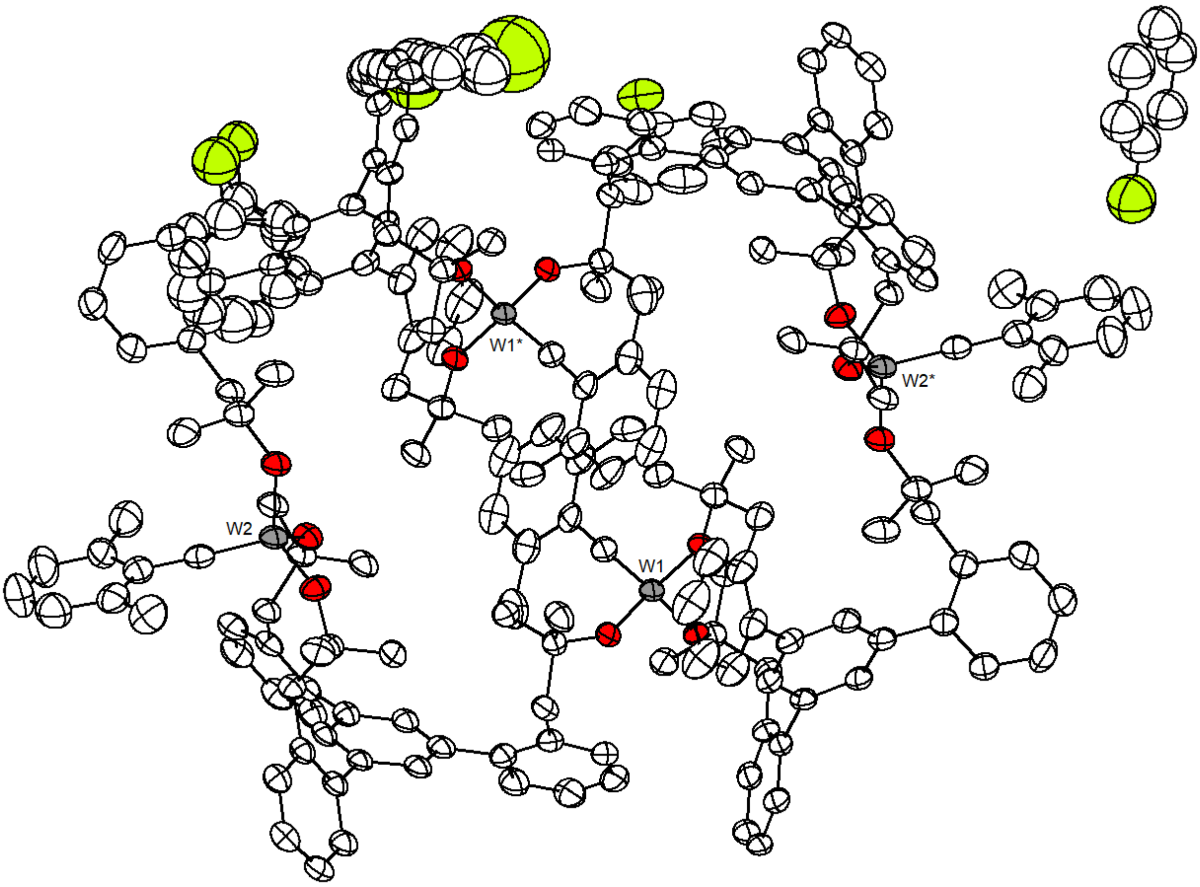

Figure S4. Plot of the molecular structure of the cyclotetrameric complex **[12 · 8 fluorobenzene]**. Atomic displacement ellipsoids shown at the 50 % probability level, H-atoms are omitted for clarity. Color code: W = grey, O = red, F = yellow-green.

X-ray Crystal Structure Analysis of Complex [12 · 8 fluorobenzene]: C_{114} H_{116} F_4 O_6 W_2, M_r = 2025.76 g mol^{-1}, yellow needle, crystal size 0.02 x 0.01 x 0.01 mm^3, monoclinic, $P2_1$/n [14], a = 15.609(11) Å, b = 29.629(4) Å, c = 21.072(8) Å, β = 96.054(19)°, V = 9691(8) Å^3, T = 100(2) K, Z = 4, D_{calc} = 1.388 g·cm^3, λ = 0.6199 Å, $\mu(\lambda)$ = 1.712 mm^{-1}, no absorption correction, P11 beamline at PETRAIII (DESY, Hamburg) synchrotron facility equipped with single ϕ-axis goniometer and Pilatus 6M detector, 1.038 < θ < 24.410°, 156324 measured reflections, 23365 independent reflections, 17198 reflections with $I > 2\sigma(I)$, R_{int} = 0.0947. The structure was solved by *SHELXT* and refined by full-matrix least-squares (*SHELXL*) against F^2 to R_1 = 0.0567 [$I > 2\sigma(I)$], wR_2 = 0.1486, 1102 parameters, 137 restraints. **CCDC-2086711**

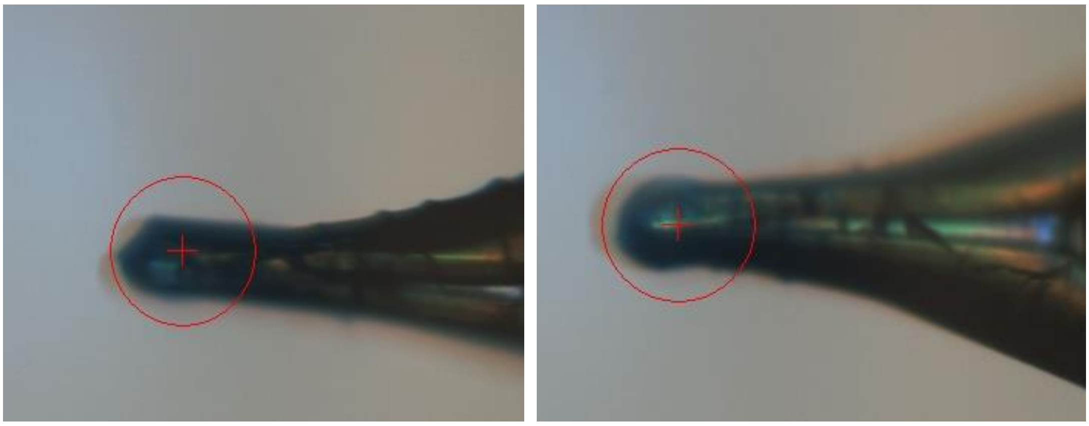

Figure S5. Images of the mounted crystal on a 30 µm MiTeGen loop at 180° and 270° ϕ-angle. Red cycle and cross indicating the profile of 100 µm primary beam.

INTENSITY STATISTICS FOR DATASET

Resolution	#Data	#Theory	%Complete	Redundancy	Mean I	Mean I/s	Rmerge	Rsigma
Inf - 2.87	467	469	99.6	6.83	88.46	28.81	0.0250	0.0294
2.87 - 1.90	1090	1091	99.9	6.59	44.08	24.30	0.0396	0.0321
1.90 - 1.50	1564	1571	99.6	6.99	33.24	22.85	0.0468	0.0324
1.50 - 1.31	1524	1535	99.3	6.35	21.78	18.68	0.0609	0.0389
1.31 - 1.18	1679	1715	97.9	6.66	16.40	16.57	0.0757	0.0426
1.18 - 1.10	1436	1467	97.9	6.84	11.91	14.25	0.0884	0.0499
1.10 - 1.03	1663	1701	97.8	6.62	9.27	12.28	0.1125	0.0603
1.03 - 0.98	1490	1517	98.2	6.32	7.78	10.52	0.1337	0.0715
0.98 - 0.93	1812	1872	96.8	6.49	6.32	9.35	0.1593	0.0826
0.93 - 0.90	1264	1327	95.3	6.54	5.39	8.30	0.1791	0.0940
0.90 - 0.87	1471	1509	97.5	6.69	4.53	7.31	0.2064	0.1092
0.87 - 0.84	1639	1737	94.4	6.15	3.98	6.28	0.2387	0.1303
0.84 - 0.81	1907	1987	96.0	5.96	3.38	5.26	0.2771	0.1600
0.81 - 0.79	1454	1530	95.0	6.08	3.11	4.84	0.2962	0.1744
0.79 - 0.77	1596	1682	94.9	6.12	2.66	4.33	0.3395	0.2061
0.77 - 0.75	1715	1819	94.3	6.18	2.28	3.76	0.3892	0.2415
0.75 - 0.74	967	1013	95.5	5.69	1.98	3.11	0.4421	0.3001
0.74 - 0.72	2030	2180	93.1	5.53	1.89	2.93	0.4567	0.3225
0.72 - 0.71	1090	1150	94.8	5.70	1.64	2.53	0.4997	0.3808
0.71 - 0.69	2392	2590	92.4	5.60	1.43	2.23	0.5477	0.4435
0.69 - 0.68	627	857	73.2	3.82	0.83	1.20	0.7042	0.9499
0.78 - 0.68	9673	10521	91.9	5.60	1.82	2.87	0.4571	0.3367
Inf - 0.68	30877	32319	95.5	6.18	9.98	9.24	0.1041	0.0676

Because of small crystal size and high reactivity of this compound, an investigation with synchrotron radiation (λ = 0.61990 Å) was undertaken at P11@PETRAIII (DESY, Hamburg). A resolution cut off (SHEL 99 0.75) was applied to suppress poorly measured intensities at higher diffraction angles. Disorder solute molecules (two fluorobenzene molecules with 50:50 occupancy) are modeled using DSR tool implemented in OLEX2. Solute molecules are partially described by isotropic displacement parameters. The high residual electron density (SHELXL: Highest peak 2.37 e A^{-3} at 0.6997 0.5226 0.7755, 0.90 Å from W2) could possibly be caused by anharmonic displacement of the W2 atom. No absorption correction was applied but the fact that only one W atom is affected indicates that absorption effects are not the cause of the residual electron density near to W2.

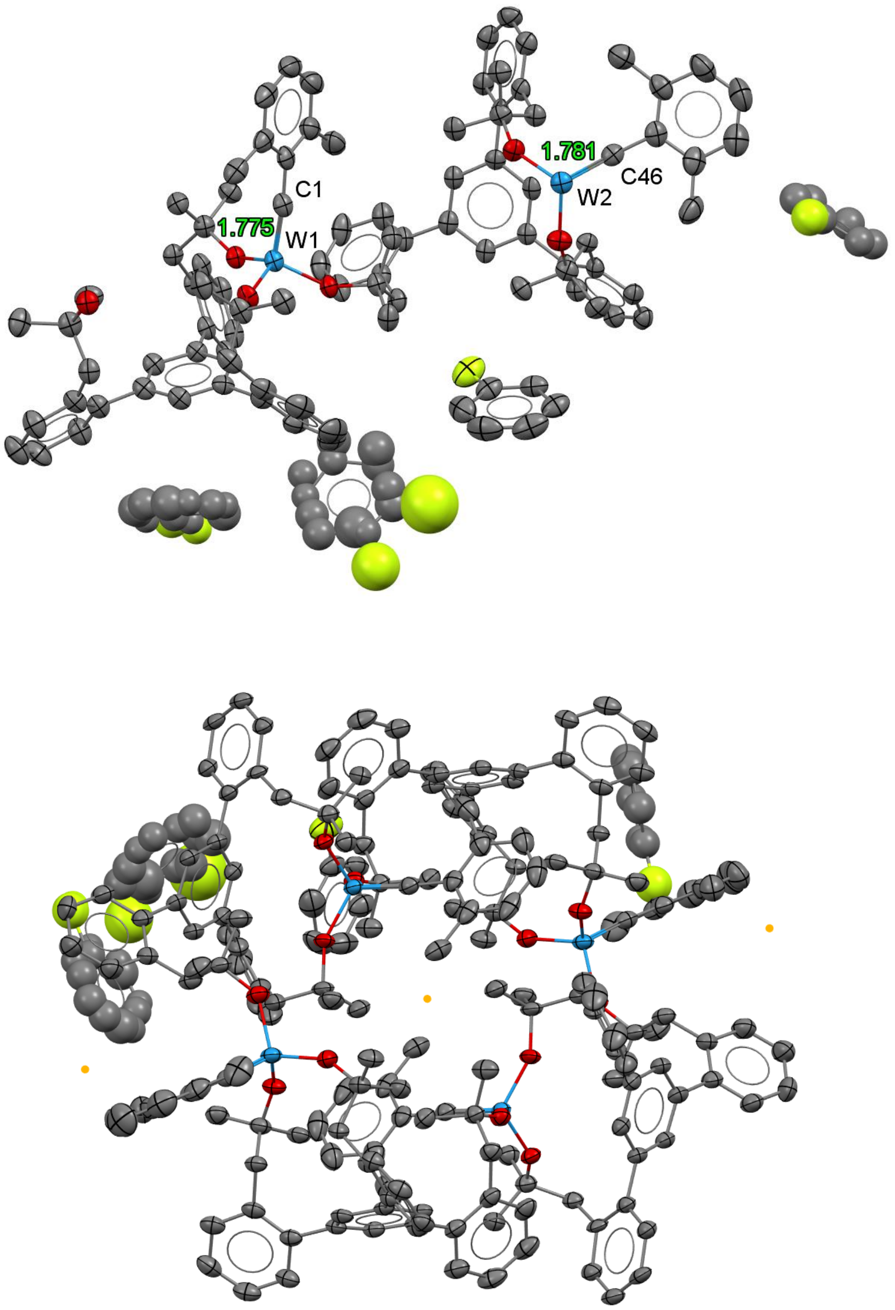

Figure S6. Asymmetric unit of the tetrameric structure with W≡C bond lengths of both independent molecules (top). Tetrameric structure of complex **12** with a crystallographic inversion centre (orange dot) in middle of the entity (bottom).

It was found that the compound forms monoclinic crystals ($P2_1$/n) with unit cell dimensions of a = 15.609(11) Å, b = 29.629(4) Å, c = 21.072(8) Å, β = 96.054(19)°, a total unit cell volume of V = 9691(8) Å^3 and Z = 4. One-half of the centrosymmetric tetramer comprises the crystallographic asymmetric unit. Each tungsten atom is situated in an almost ideal tetrahedral coordination sphere of three alkoxy oxygen and one alkyne carbon atom. The average W–O distance is 1.868(7) Å and the W-O-C angles ranges from 139.47 to 153.03°. The W–C bond lengths are 1.775(7) and 1.782(7) Å, W-C-C bond angles are 173.5(5) and 175.6(5)°. All four tungsten entites are interconnected by four tripodal ligands. Each ligand is coordinating in a 1:2 ratio to two individual tungstens atoms. No hydrogen bonds or W-π interactions could be found in the described structure.

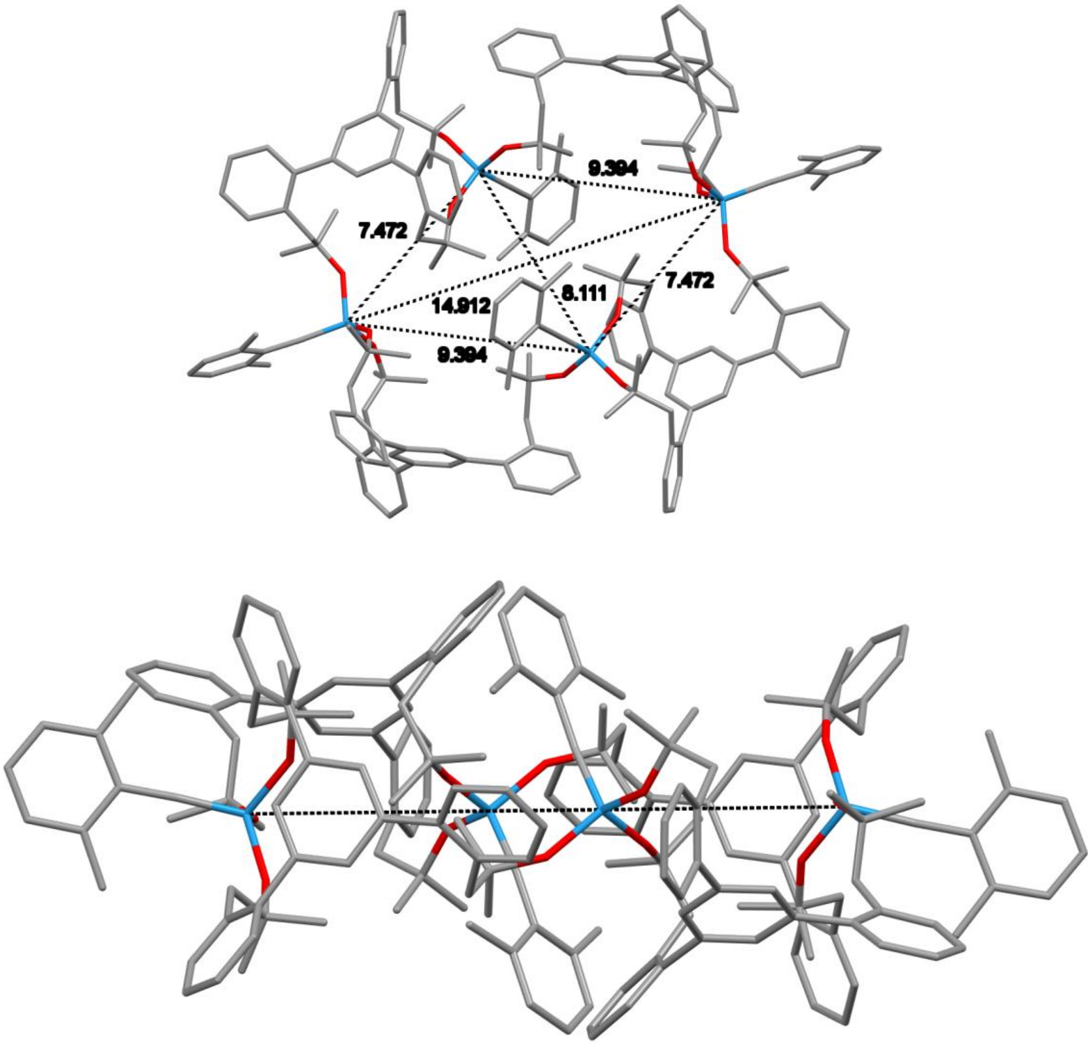

Figure S7. The arrangement of the four W atoms in the complex **12**, showing the non-bonding W····W distances (top) and coplanar geometry (bottom).

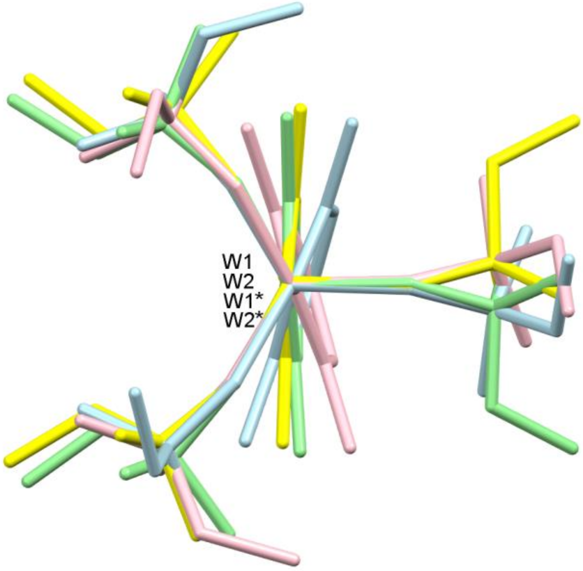

Figure S8. Overlay of the local environments (WO_3C) of the four W atoms in the complex **12**, showing the relative conformation of the respective 2,6-dimethylbenzylidyne groups.

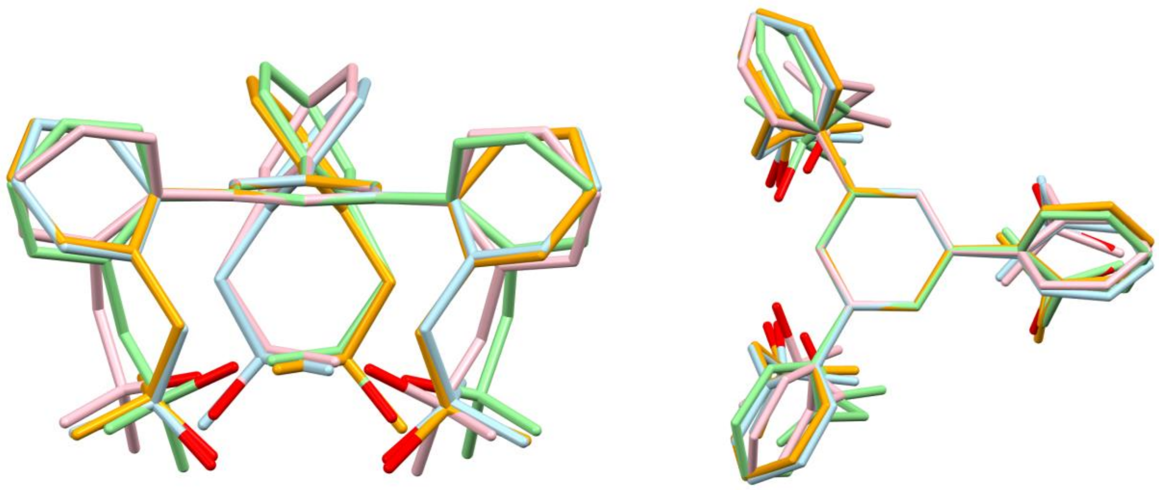

Figure S9. Overlay of the central C6 ring of the four podand ligands in the tetrameric complex **12**, showing their relative conformations (side and top views, O atoms shown in red).

A search for similar structures in the CSD database (performed on May 28, 2021 using ConQuest 2020.2.0; CSD version 5.41 (November 2019) + 1 update) with the input shown in the Insert resulted in 17 hits in the database. Among them, 7 similar structures ($(O)_3W{\equiv}C$-Aryl) could be identified. The relevant geometries are summarized below.

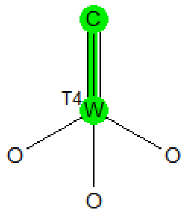

Table S1. CSD search results

CCDC refcode	W≡C bond length (Å)	C–C–W bond angle (°)
COXVOA and COXVOA10	1.757	175.79
FEWPOO	1.760	176.01
UJEYUE	1.745	174.07
VIZVOS	1.759	177.91
WEJCEW	1.769	173.67
YITCIQ	1.763	178.50
Average of this study	*1.778*	*174.52*

A comparison of bond lengths and angles shows that they are in good agreement with known alkoxytungsten-alkylidyne complexes, albeit the W≡C distance is the longest in this study. It is important to mention, that all of the listed literature structures are monomeric entities.

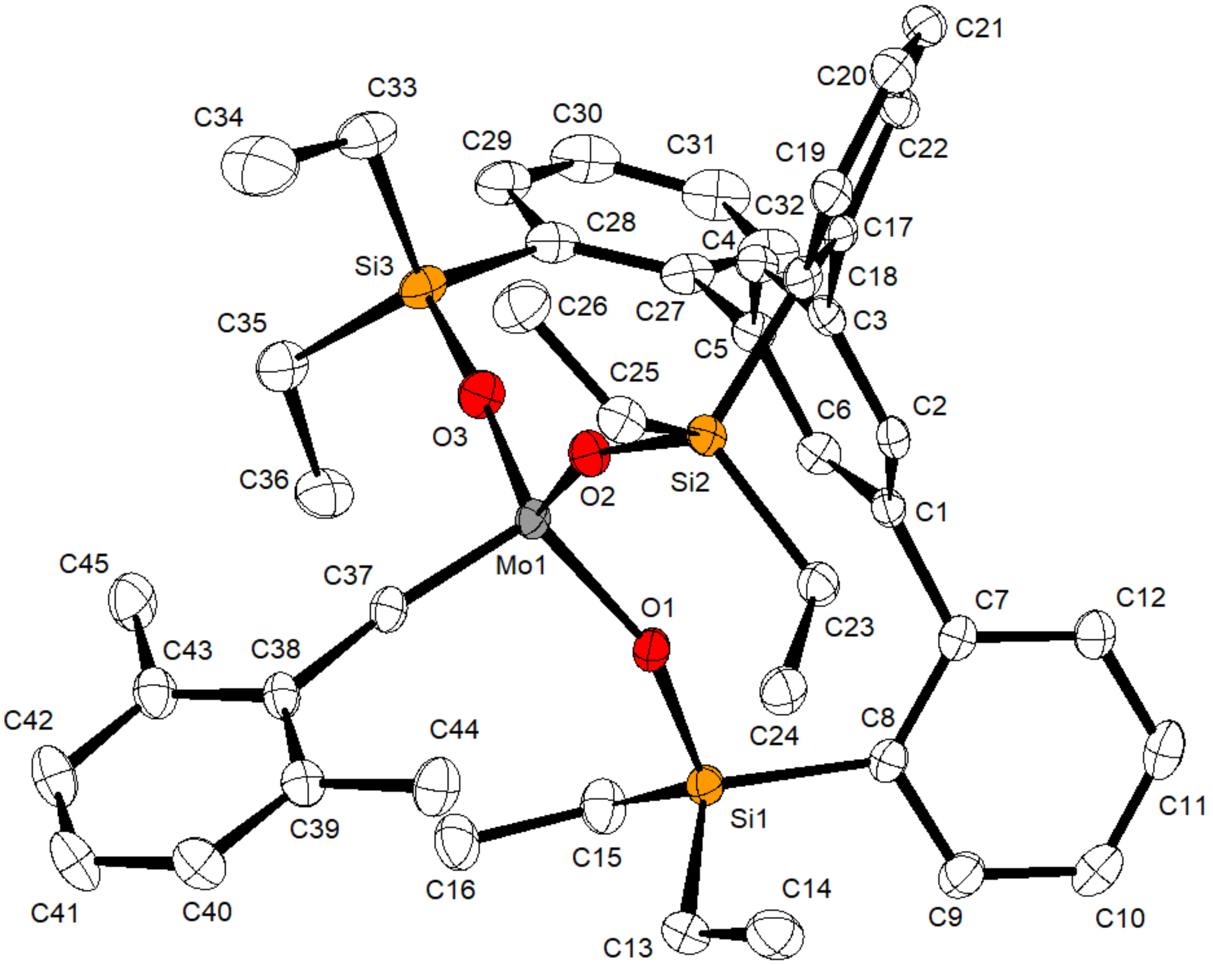

Figure S10. Plot of the molecular structure of complex **1f**; atomic displacement ellipsoids shown at the 50 % probability level, H-atoms are omitted for clarity. Color code: Mo = grey, O = red, Si = orange.

X-ray Crystal Structure Analysis of complex Complex 1f: $C_{45}H_{54}MoO_3Si_3$, M_r = 823.09 g mol^{-1}, yellow prism, crystal size 0.12 x 0.10 x 0.06 mm^3, triclinic, *P*-1 [2], a = 10.1902(8) Å, b = 11.108(3) Å, c = 20.652(5) Å, α = 95.927(14)°, β = 97.963(15)°, γ = 114.883(13)°, V = 2065.9(7) Å^3, T = 100(2) K, Z = 2, D_{calc} = 1.323 g·cm^3, λ = 0.71073 Å, μ(*Mo*-K_α) = 0.443 mm^{-1}, Gaussian absorption correction (T_{min} = 0.95095, T_{max} = 0.97694), Bruker AXS Enraf-Nonius KappaCCD diffractometer with a FR591 rotating Mo-anode X-ray source, 2.613 < θ < 33.195°, 75165 measured reflections, 15720 independent reflections, 10753 reflections with $I > 2\sigma(I)$, R_{int} = 0.0915. The structure was solved by *SHELXS* and refined by full-matrix least-squares (*SHELXL*) against F^2 to R_1 = 0.0522 [$I > 2\sigma(I)$], wR_2 = 0.1294, 477 parameters. **CCDC-2088379**

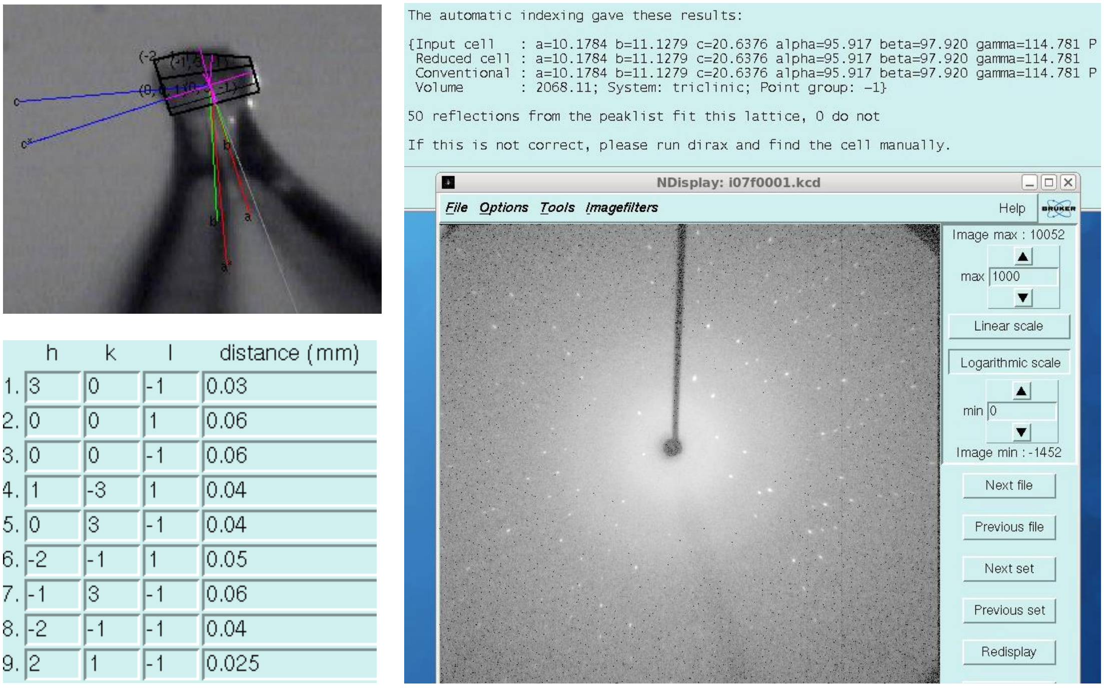

	h	k	l	distance (mm)
1.	3	0	-1	0.03
2.	0	0	1	0.06
3.	0	0	-1	0.06
4.	1	-3	1	0.04
5.	0	3	-1	0.04
6.	-2	-1	1	0.05
7.	-1	3	-1	0.06
8.	-2	-1	-1	0.04
9.	2	1	-1	0.025

Figure S11. Crystal faces and unit cell determination of complex **1f**.

INTENSITY STATISTICS FOR DATASET

Resolution	#Data	#Theory	%Complete	Redundancy	Mean I	Mean I/s	Rmerge	Rsigma
Inf - 2.59	238	248	96.0	9.71	106.70	44.89	0.0487	0.0183
2.59 - 1.76	550	552	99.6	6.78	50.26	30.97	0.0437	0.0247
1.76 - 1.39	812	813	99.9	6.15	39.66	27.35	0.0449	0.0279
1.39 - 1.22	769	769	100.0	5.95	28.82	22.57	0.0511	0.0322
1.22 - 1.11	777	777	100.0	5.81	21.69	19.49	0.0623	0.0372
1.11 - 1.03	793	793	100.0	5.66	18.64	17.62	0.0714	0.0420
1.03 - 0.97	798	798	100.0	5.48	14.58	15.22	0.0855	0.0505
0.97 - 0.92	807	807	100.0	5.22	12.53	12.82	0.0982	0.0597
0.92 - 0.88	801	801	100.0	5.03	12.00	12.15	0.1067	0.0655
0.88 - 0.84	931	931	100.0	4.79	10.86	10.47	0.1178	0.0762
0.84 - 0.81	863	864	99.9	4.60	9.56	9.25	0.1358	0.0911
0.81 - 0.79	626	626	100.0	4.44	8.62	8.18	0.1589	0.1067
0.79 - 0.77	705	705	100.0	4.38	7.59	6.95	0.1732	0.1269
0.77 - 0.75	777	777	100.0	4.23	6.75	5.74	0.2084	0.1575
0.75 - 0.73	860	860	100.0	3.99	5.81	4.48	0.2441	0.2082
0.73 - 0.71	979	979	100.0	3.91	5.45	3.90	0.2693	0.2508
0.71 - 0.70	521	521	100.0	3.73	4.91	3.14	0.3115	0.3224
0.70 - 0.68	1122	1122	100.0	3.67	4.09	2.36	0.3396	0.4417
0.68 - 0.67	655	655	100.0	3.58	4.12	2.04	0.3677	0.5111
0.67 - 0.66	670	672	99.7	3.44	3.63	1.62	0.4068	0.6471
0.66 - 0.65	667	767	87.0	2.95	3.26	1.34	0.4581	0.7832
0.75 - 0.65	5474	5576	98.2	3.63	4.53	2.79	0.3210	0.3989
Inf - 0.65	15721	15837	99.3	4.75	14.60	11.20	0.0868	0.0841

One reflection (4 -3 3) was omitted from dataset before the final refinement cycles. Complete .cif-data of the compound are available under **CCDC-2088379**.

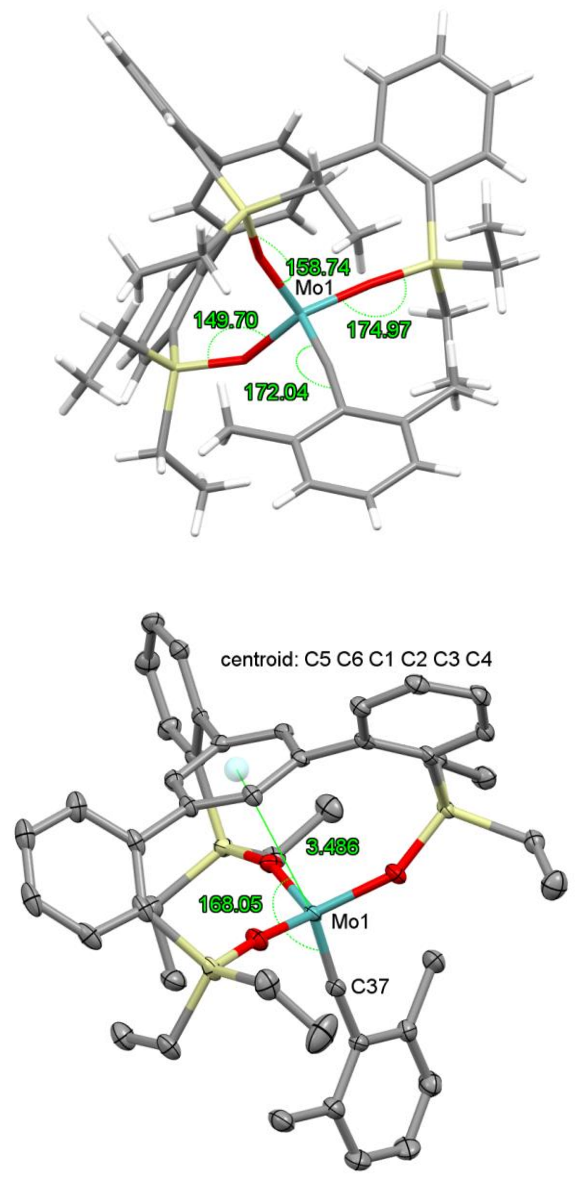

Figure S12. The molecular structure of complex **1f**, showing the significantly different Mo-O-Si angles (top) and the arrangement of the basal phenyl ring with respect to the alkylidyne moiety (bottom).

Crystal Structure Survey. A search for similar structures in the CSD database (performed on May 28, 2021 using ConQuest 2020.2.0; CSD version 5.41 (November 2019) + 1 update) performed with the input shown in the Insert resulted in 8 hits in the database. Two of them are very similar structures with a tridentate ligands and the remaining six structures containing three individual monodentate ligands. There relevant geometries are summarized below.

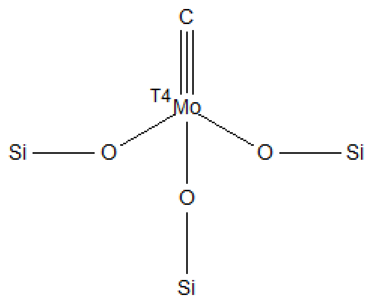

Table S2. CSD search results

Refcode	Mo-O1-Si1 (°)	Mo-O2-Si2 (°)	Mo-O3-Si3 (°)	Mo≡C (Å)	Mo-O1 (Å)	Mo-O2 (Å)	Mo-O3 (Å)	ligand type	**Si** substi-tuent
**QOSSAV**	172.145	161.593	168.544	1.746	1.878	1.870	1.861	tridentate	aryl
**QOSSEZ**	166.533	165.165	161.897	1.741	1.866	1.877	1.882	tridentate	aryl
**QOSSID**	160.175	139.936	147.255	1.748	1.880	1.892	1.880	monodentate	aryl
**QOSSOJ**	173.494	145.472	143.408	1.749	1.876	1.884	1.893	monodentate	aryl
**LEKFOY**	154.099	146.365	169.420	1.748	1.887	1.886	1.881	monodentate	aryl
**LEKHAM**	159.456	147.725	141.255	1.745	1.884	1.880	1.887	monodentate	aryl
**LEKHAM**	142.463	162.902	149.908	1.747	1.890	1.876	1.882	monodentate	aryl
**POJDEZ**	145.047	143.963	147.999	1.730	1.888	1.889	1.892	monodentate	OtBu
**POJDUP**	164.085	147.633	145.704	1.734	1.843	1.877	1.885	monodentate	OtBu
**POJDUP**	144.533	124.646	148.746	1.741	1.901	1.918	1.884	monodentate	OtBu
**1f**	158.74	149.70	174.96	1.751	1.869	1.890	1.893	tridentate	alkyl

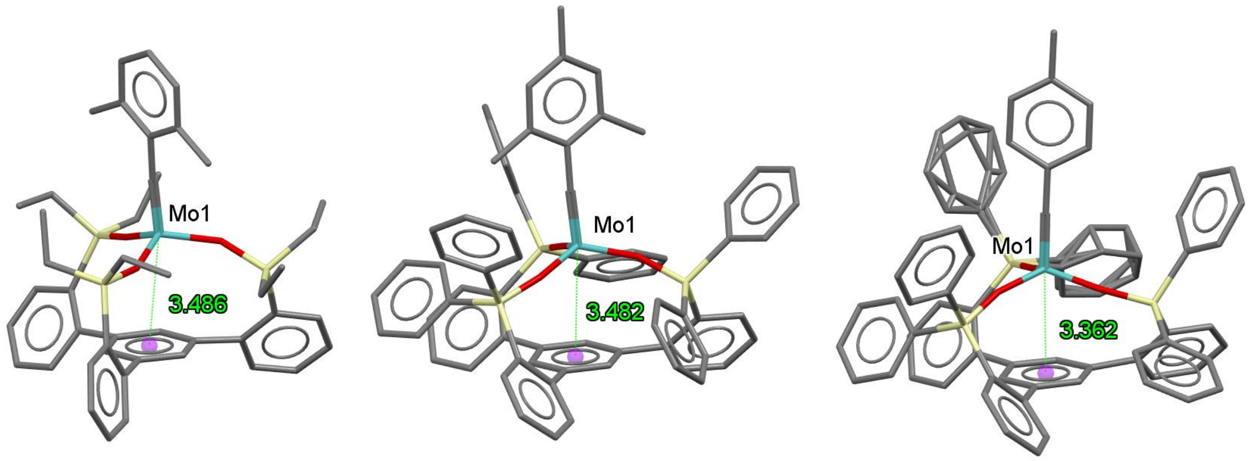

Figure S13. Comparison of the distances between the central Mo atom and calculated centroids of the basal aryl ring of the tripodal ligand framework: Complex **1f** (left), QOSSAV (middle) and QOSSEZ (right).

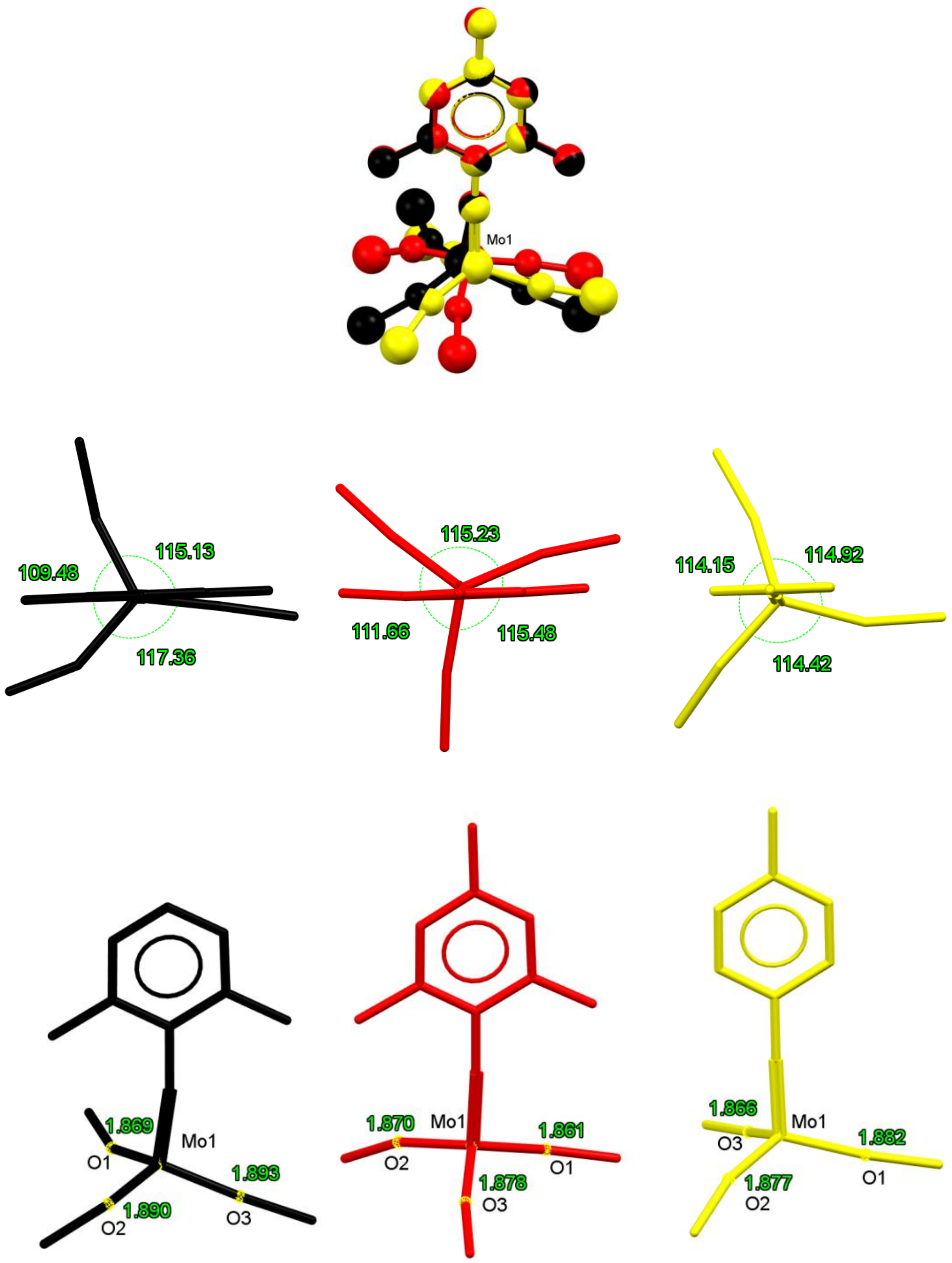

Figure S14. The local environments of the Mo atoms in complex **1f** (black), QOSSAV (red) and QOSSEZ (yellow).

General. Unless stated otherwise, all reactions were carried out under Ar in flame-dried glassware. The solvents were purified by distillation over the drying agents indicated and were transferred under Ar: THF, Et_2O, 1,4-dioxane (Mg/anthracene), CH_2Cl_2, DME, MeCN (CaH_2), *n*-pentane, benzene, toluene (Na/K). Flash chromatography on silica gel (FC): Merck silica gel 60 (230–400 mesh).

All commercially available compounds (Fluka, Lancaster, Aldrich) were used as received, unless stated otherwise. The molecular sieves used in this investigation were dried for 24 h at 150°C (sand bath) under vacuum prior to use and were stored and transferred under argon atmosphere.

IR: Spectrum One (Perkin-Elmer) spectrometer, wavenumbers ($\tilde{\nu}$) in cm^{-1}. MS (EI): Finnigan MAT 8200 (70 eV), ESI-MS: ESQ3000 (Bruker), accurate mass determinations: Bruker APEX III FT-MS (7 T magnet) or Mat 95 (Finnigan). Elemental analysis: H. Kolbe, Mülheim/Ruhr.

NMR: Spectra were acquired on Bruker AvanceIII 400, 500 MHz or AVneo 600 MHz NMR spectrometers in the solvents indicated; the AVneo 600 MHz NMR spectrometer was equipped with a Bruker BBO CryoProbe, which significantly reduced the measurement time of most of the spectra, especially the 1D ^{13}C NMR data.

Chemical shifts (δ) are given in ppm relative to TMS, coupling constants (*J*) in Hz. The solvent signals were used as references and the chemical shifts converted to the TMS scale ($CDCl_3$: $\delta_C \equiv 77.0$ ppm; residual $CHCl_3$ in $CDCl_3$: $\delta_H \equiv 7.26$ ppm; CD_2Cl_2: $\delta_C \equiv 53.8$ ppm; residual $CHDCl_2$: $\delta_H \equiv 5.32$ ppm; [D_8]-toluene: $\delta_C \equiv 20.4$ ppm; residual $D_5C_6CD_2H$: $\delta_H = 2.09$ ppm).

^{95}Mo NMR spectra were acquired with the aring pulse sequence to minimize acoustic ringing from the NMR probe. The π/2 pulse was calibrated for a 2 M Na_2MoO_4 in D_2O and had a typical length of 22.5 µs at a power of 85W. Chemical shifts were referenced indirectly to the ^1H chemical shift of the solvent.[1] For broad signals, larger amounts of the sample (> 40 mg) were necessary. Dependent on the line width of the signal, 8000 to 150000 FID containing 8192 complex data points were averaged to obtain a reasonable signal-to-noise ratio. The acquisition time of a single FID was around 150 ms. The data was Fourier-transformed with zero-filling to 8192 data points and with a line broadening lb = 20 Hz, unless noted otherwise.

Diffusion coefficients were obtained from a double stimulated echo sequence with bipolar gradient pulses, convection compensation, longitudinal eddy current delay (LED) and three spoiler gradients (Bruker sequence: dstebpgp3s). The gradient pulse strength G was incremented from 2% to 98% of the maximum G_{max} with a squared gradient ramp in 60 steps. The diffusion time (Δ) used was 71 ms and the length of a gradient pulse gradient pulse (δ/2) of the encoding gradient was 1.3 ms. The maximum gradient strength G_{max} of the NMR probe (PA BBO 400S1 BBF-H-D-05 Z PLUS) was 53.5 $G\cdot cm^{-1}$. Diffusion coefficients were obtained by averaging three diffusion coefficients obtained from fitting the signal decay of three different resonance integrals to the Stejskal-Tanner equation (I) in the Bruker TOPSPIN T1T2 relaxation module:

$$I(G) = I_0 e^{-D(\gamma G \delta)^2(\Delta - \delta/3)} \qquad \text{(I)}$$

Diffusion values were predicted using an EXCEL spreadsheet Stokes–Einstein Gierer-Wirtz Estimation (SEGWE) method.[2]

New Ligands.

(5'-(2-(Diethylsilyl)phenyl)-[1,1':3',1''-terphenyl]-2,2''-diyl)bis(diethylsilane) (S1).[3] A two-necked, round-bottomed flask was equipped with a magnetic stir bar and a gas inlet connected to an argon-vacuum manifold. The flame-dried flask was filled with argon and charged with 1,3,4-tris-2′-bromophenylbenzene (2.00 g, 3.68 mmol)[4,5] and Et_2O (77 mL). The resulting mixture was cooled to −125 °C (pentane/liquid nitrogen bath). A solution of *tert*-butyllithium (14.2 mL, 22.6 mmol, 1.6 M in *n*-pentane) was added dropwise and the resulting mixture was allowed to warm to ambient temperature. After stirring for 1 h, the mixture was cooled to −125 °C and diethylsilane (2.86 mL, 22.1 mmol) was added dropwise. The mixture was then warmed to ambient temperature and stirring was continued overnight. The reaction was carefully quenched with water and the resulting mixture was transferred into a separation funnel. The organic phase was separated and the aqueous solution was extracted with ethyl acetate (3 x 50 mL). The combined organic layers were dried over $MgSO_4$, filtered and concentrated in vacuo. The residue was purified by flash chromatography on silica gel (hexanes/ethyl acetate, 10:1) to give the title compound as a colorless solid (1.9 g, 91%). ^{1}H NMR (400 MHz, CD_3Cl): δ = 7.58 – 7.54 (m, 3H), 7.42 – 7.36 (m, 3H), 7.35 – 7.28 (m, 6H), 7.22 (s, 3H), 4.13 (p, *J* = 3.4 Hz, 3H), 0.84 (t, *J* = 7.8 Hz, 18H), 0.60 (m, 12H). ^{13}C NMR (101 MHz, $CDCl_3$): δ = 149.4, 143.1, 136.0, 134.2, 129.6, 129.1, 128.9, 126.4, 8.5, 4.2. IR (film): ṽ 3051, 2952, 2872, 2102, 1583, 1558, 1460, 1409, 1378, 1260, 1230, 1124, 1098, 1063, 1006, 970, 891, 873, 736, 686, 640, 623, 607, 527, 458 cm^{-1}. HRMS-APPI (*m/z*): calcd. for $C_{36}H_{48}Si_3$ $[M]^{\cdot+}$, 564.30584; found, 564.30642.

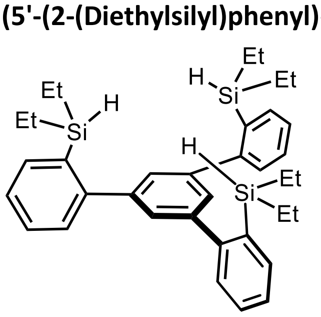

Compound S2. A 50 mL, two-necked flask equipped with an argon manifold was charged with 1,3,4-tris-2′-bromophenylbenzene (300 mg, 0.55 mmol)[4,5] and diethyl ether (20 mL). The resulting mixture was cooled to −125 °C using a bath of *n*-pentane and liquid nitrogen. A solution of *tert*-butyllithium (2.10 mL, 3.37 mmol, 1.6 M in *n*-pentane) was added dropwise and the mixture was allowed to warm to ambient temperature. After stirring for 1 h, the mixture was cooled to −125 °C before di-*n*-butyl silane (0.64 mL, 3.31 mmol) was added dropwise. The mixture was then warmed to ambient temperature and stirring was continued overnight. The reaction was carefully quenched with water (15 mL), the layers were separated and the aqueous layer was extracted with dichloromethane (3 × 20 mL). The combined organic layers were dried over magnesium sulfate, filtered, and the filtrate was evaporated. The residue was purified by flash chromatography on silica gel (*n*-pentane) to give the title compound as a colorless oil (362 mg, 89%). ^{1}H NMR (500 MHz, $CDCl_3$) δ 7.58 (ddd, *J* = 7.4, 1.6, 0.6 Hz, 3H), 7.41 – 7.28 (m, 9H), 7.23 (s, 1H), 4.17 (p, *J* = 3.6 Hz, 3H), 1.25 – 1.11 (m, 24H), 0.78 – 0.69 (m, 18H), 0.69 – 0.55 (m, 12H). ^{13}C NMR (126 MHz, $CDCl_3$) δ 149.3, 143.1, 136.0, 134.7, 129.5, 129.0, 128.9, 126.4, 27.1, 26.3, 13.8, 12.7. ^{29}Si NMR (99 MHz, $CDCl_3$) δ −10.4. IR (film): ṽ 2955, 2920, 2871, 2855, 2103, 1583, 1464, 1408, 1377, 1189, 1123, 1098, 1080, 1064, 1027, 889, 805, 757, 731, 686, 635, 623, 460. HRMS (ESI) calcd. for $C_{48}H_{71}Si_3$ $[M{-}H]^-$: 731.48691; found: 731.48625.

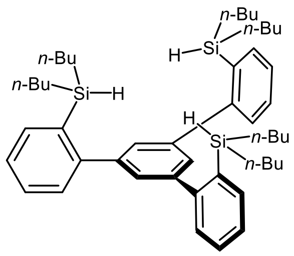

Compound S3.[3] A two-necked, round-bottomed flask was equipped with a magnetic stir bar and a gas inlet connected to an argon-vacuum manifold. The flame-dried flask was filled with argon and charged with 1,3,4-tris-2′-bromophenylbenzene (1.50 g, 2.76 mmol)[4,5] and Et_2O (58 mL). The resulting mixture was cooled to −125 °C (pentane/liquid nitrogen bath). A solution of *tert*-butyllithium (8.94 mL, 17.0 mmol, 1.9 M in *n*-pentane) was added dropwise and the mixture was allowed to warm to ambient temperature. After stirring for 1 h at ambient temperature, the mixture was cooled to −125 °C before di-*iso*-butylchlorosilane (3.07 mL, 16.6 mmol) was added dropwise. The mixture was warmed to ambient temperature and stirring was continued overnight. The reaction was carefully quenched with water and the resulting mixture was transferred into a separation funnel. The organic phase was separated and the aqueous solution extracted with ethyl acetate (3 x 50 mL). The combined organic layers were dried over $MgSO_4$, filtered and concentrated in vacuo. The residue was purified by flash chromatography on silica gel (hexanes/*t*-butyl methyl ether, 99:1) and the product dried in high vacuum (70°C, 10^{-3} mbar, 3 h) to give the title compound as a colorless oil (1.56 g, 77%). ^1H NMR (600 MHz, CD_3Cl): δ = 7.64 (ddd, *J* = 7.4, 1.4, 0.6 Hz, 3H), 7.40 – 7.36 (m, 6H), 7.35 – 7.31 (m, 3H), 7.27 (s, 3H), 4.35 – 4.28 (m, 3H), 1.68 – 1.57 (m, 6H), 0.82 – 0.75 (m, 36H), 0.68 – 0.56 (m, 12H). ^{13}C NMR (151 MHz, $CDCl_3$): δ = 149.3, 143.0, 136.3, 135.1, 129.6, 129.1, 129.0, 126.4, 26.1, 25.7, 25.5, 24.3. ^{29}Si NMR (119 MHz, $CDCl_3$): δ = −14.4. IR (film): $\tilde{\nu}$ 3053, 2952, 2895, 2867, 2826, 2113, 1584, 1558, 1463, 1408, 1382, 1364, 1328, 1261, 1203, 1163, 1124, 1085, 1034, 950, 891, 849, 759, 740, 723, 637, 623, 527, 461, 417 cm^{-1}. HRMS-APPI (*m/z*): calcd. for $C_{48}H_{72}Si_3$ $[M]^+$, 732.49364; found, 732.49398.

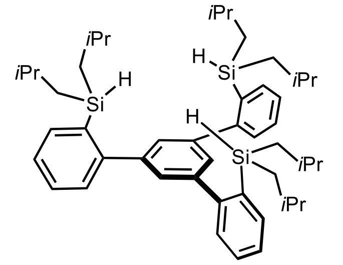

Compound S4. A 100 mL, three-necked flask equipped with a 25 mL dropping funnel and an argon manifold was charged with 1,3,4-tris-2′-bromophenylbenzene (1.00 g, 1.84 mmol)[4,5] and diethyl ether (60 mL). The resulting mixture was cooled to −125 °C using a bath of *n*-pentane and liquid nitrogen. A solution of *tert*-butyllithium (6.60 mL, 11.2 mmol, 1.7 M in *n*-pentane) was added dropwise *via* the dropping funnel and the mixture was allowed to warm to ambient temperature. After stirring for 1 h, the mixture was cooled to −125 °C before di-*n*-octyl silane (2.36 g, 9.21 mmol) was added dropwise. The mixture was then allowed to warm to ambient temperature and stirring was continued for 3 d. The reaction was carefully quenched with ethanol (3 mL) and water (40 mL). The layers were separated and the aqueous layer was extracted with diethyl ether (3 × 40 mL). The combined organic layers were dried over magnesium sulfate, filtered, and the solvents were evaporated. The residue was purified by flash chromatography (*n*-pentane) to give the title compound as a yellow liquid (1.00 g, 51%). ^1H NMR (500 MHz, $CDCl_3$) δ 7.60 (ddd, *J* = 7.3, 1.5, 0.6 Hz, 3H), 7.40 (td, *J* = 7.7, 7.2, 1.5 Hz, 3H), 7.36 (ddd, *J* = 7.7, 1.5, 0.6 Hz, 3H), 7.33 (td, *J* = 7.2, 1.5 Hz, 3H), 7.27 (s, 3H), 4.19 (p, *J* = 3.6 Hz, 3H), 1.31 – 1.08 (m, 72H), 0.86 (t, *J* = 7.2 Hz, 18H), 0.66 – 0.58 (m, 12H). ^{13}C NMR (126 MHz, $CDCl_3$) δ 149.3, 143.1, 136.0, 134.7, 129.5, 129.0, 128.9, 126.4, 33.5, 32.1, 29.4, 29.4, 25.0, 22.8, 14.3, 13.0. ^{29}Si NMR (99 MHz, $CDCl_3$) δ −10.3. IR (film): $\tilde{\nu}$ 2956, 2920, 2851, 2127, 2102, 1465, 1409, 1123, 1099, 1064, 1002, 915, 891, 871, 834, 810, 758, 721, 686, 635, 623, 460. HRMS (ESI) calcd. for $C_{72}H_{120}Si_3$ $[M]^+$: 1068.8692; found: 1068.8700.

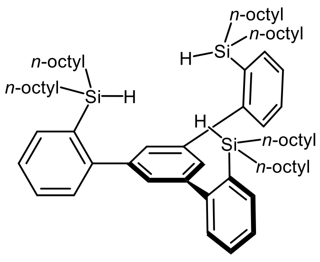

Ligand 3f.[3] A one-neck round bottomed flask equipped with a stir bar was charged with silane **S1** (222 mg, 0.393 mmol) and CH_2Cl_2 (5 mL). The resulting mixture was cooled to 0 °C. *m*-Chloroperbenzoic acid (77% *w/w*, 291 mg, 1.30 mmol) was added in portions and the mixture was allowed to warm to ambient temperature. After 4 h, the mixture was carefully transferred into a separation funnel, diluted with CH_2Cl_2 (10 mL), and washed with sat. $NaHCO_3$ (4 x 15 mL) and brine (3 x 10 mL). The organic phase was then dried over $MgSO_4$, filtered and concentrated *in vacuo* to give the title compound as a colorless solid material (240 mg, 99%). ^{1}H NMR (400 MHz, $CDCl_3$): δ = 7.51 – 7.47 (m, 3H), 7.43 – 7.33 (m, 6H), 7.33 – 7.30 (m, 3H), 7.24 (s, 3H), 0.90 – 0.85 (m, 18H), 0.75 (m, 12H). ^{13}C NMR (101 MHz, $CDCl_3$): δ = 149.0, 144.1, 135.9, 134.6, 129.9, 128.9, 127.6, 126.4, 8.2, 6.9. ^{29}Si NMR (119 MHz, $CDCl_3$): δ = 7.7. IR (film): ṽ 3318, 3051, 2955, 2912, 2875, 1583, 1558, 1461, 1410, 1378, 1260, 1235, 1162, 1124, 1090, 1065, 1005, 959, 908, 888, 822, 760, 711, 615, 530, 512, 465 cm^{-1}. HRMS-ESI (*m/z*): calcd. for $C_{36}H_{47}O_3Si_3$ [M-H]$^-$, 611.28386; found, 611.28383.

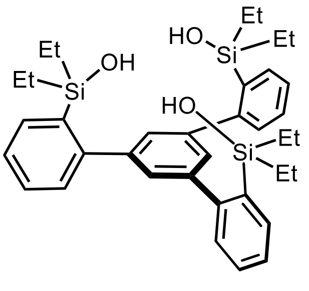

Ligand 3g. A 50 mL, one-necked flask open to air was charged with silane **S2** (362 mg, 0.49 mmol) and dichloromethane (5 mL). The resulting mixture was cooled to 0°C. *m*-Chloroperoxybenzoic acid (77% *w/w*, 365 mg, 1.63 mmol) was added in portions and the resulting mixture stirred at ambient temperature for 5 h. The mixture was diluted with dichloromethane (30 mL), transferred into a separation funnel, and washed with saturated aqueous solutions of sodium bicarbonate (3 × 50 mL) and brine (50 mL). The organic layer was dried over magnesium sulfate, filtered, and the solvents were evaporated to give the title compound as a white solid material (273 mg, 71%). ^{1}H NMR (500 MHz, $CDCl_3$) δ 7.49 (dd, *J* = 7.3, 1.6 Hz, 3H), 7.42 – 7.31 (m, 9H), 7.27 (s, 3H), 3.56 (s, 3H), 1.31 – 1.14 (m, 27H), 0.82 – 0.67 (m, 27H). ^{13}C NMR (126 MHz, $CDCl_3$) δ 148.6, 144.0, 136.4, 134.5, 129.5, 128.7, 127.6, 126.2, 26.4, 25.2, 16.8, 13.5. ^{29}Si NMR (99 MHz, $CDCl_3$) δ 5.8. IR (film): ṽ 3298, 3051, 2956, 2922, 2871, 2857, 1584, 1464, 1409, 1377, 1194, 1124, 1080, 1024, 999, 964, 884, 826, 760, 735, 724, 481. HRMS (ESI) calcd. for $C_{48}H_{72}O_3Si_3Na$ [M+Na]$^+$: 803.46815; found: 803.46770.

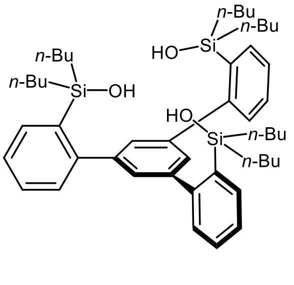

Ligand 3h.[3] A two-neck round bottomed flask equipped with a stir bar was charged with silane **S3** (563 mg, 0.768 mmol) and CH_2Cl_2 (10 mL). The resulting mixture was cooled to 0 °C. *m*-Chloroperbenzoic acid (77% *w/w*, 568 mg, 2.53 mmol) was added in portions and the resulting mixture was allowed to warm to ambient temperature. After 4 h, the mixture was carefully transferred into a separation funnel, diluted with CH_2Cl_2 (10 mL) and washed with sat. $NaHCO_3$ (4 x 15 mL) and brine (3 x 10 mL). The organic phase was then dried over $MgSO_4$, filtered and concentrated *in vacuo* to give the title compound as a colorless solid material (584 mg, 97%). ^{1}H NMR (600 MHz, $CDCl_3$): δ = 7.54 – 7.51 (m, 3H), 7.40 – 7.36 (m, 3H), 7.34 – 7.32 (m, 6H), 7.27 (s, 3H), 1.75 (hept, *J* = 6.6 Hz, 6H), 0.80 (m, 36H), 0.78 – 0.69 (m, 12H). ^{13}C NMR (151 MHz, $CDCl_3$): δ = 148.5, 144.1, 137.4, 135.0, 129.7, 128.9, 127.9, 126.2, 28.5, 26.6, 26.3, 24.3. ^{29}Si NMR (119 MHz, $CDCl_3$): δ = 4.5. IR (film): ṽ 3449, 2951, 2924, 2894, 2866, 1584, 1463, 1435, 1409, 1381, 1364, 1328, 1219, 1163, 1123, 1088, 1064, 1033, 951, 908, 889, 830, 814, 759, 733, 667, 643, 622, 528, 487, 468 cm^{-1}. HRMS-ESI (*m/z*): calcd. for $C_{48}H_{72}O_3Si_3Na$ [M+Na]$^+$, 803.46815; found, 803.46891.

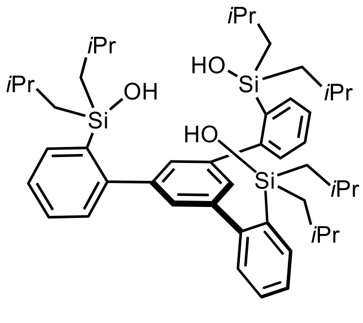

Ligand 3i. A 50 mL, one-necked flask open to air was charged with silane **S4** (999 mg, 0.93 mmol) and dichloromethane (25 mL). The resulting mixture was cooled to 0°C. *m*-Chloroperoxybenzoic acid (77% *w/w*, 690 mg, 3.08 mmol) was added in portions and the resulting mixture was stirred at ambient temperature for 5 h. The mixture was diluted with dichloromethane (40 mL), transferred into a separation funnel, and washed with saturated aqueous solutions of sodium bicarbonate (3 × 50 mL) and brine (50 mL). The organic layer was dried over magnesium sulfate, filtered, and the solvents were evaporated. The residue was purified by flash column chromatography (*n*-pentane /*tert*-butyl methyl ether, 50:1) to give the title compound as a colorless oil (898 mg, 86%). ^{1}H NMR (600 MHz, $CDCl_3$) δ 7.48 (dd, *J* = 7.3, 1.5 Hz, 3H), 7.38 (td, *J* = 7.5, 1.5 Hz, 3H), 7.34 (td, *J* = 7.3, 1.4 Hz, 3H), 7.31 (dd, *J* = 7.5, 1.4 Hz, 3H), 7.25 (bs, 3H), 3.62 (s, 3H), 1.30 – 1.03 (m, 72H), 0.86 (t, *J* = 7.2 Hz, 18H), 0.80 – 0.66 (m, 12H). ^{13}C NMR (151 MHz, $CDCl_3$) δ 148.8, 144.1, 136.6, 134.7, 129.7, 128.9, 127.8, 126.4, 33.7, 32.1, 29.4, 29.3, 23.3, 22.8, 17.2, 14.3. ^{29}Si NMR (119 MHz, $CDCl_3$) δ 5.6. IR (film): $\tilde{\nu}$ 3250, 2956, 2921, 2853, 1466, 833, 760, 722, 737. HRMS (ESI) calcd. for $C_{72}H_{119}O_3Si_3$ [M−H]$^-$: 1115.84726; found: 1115.84780.

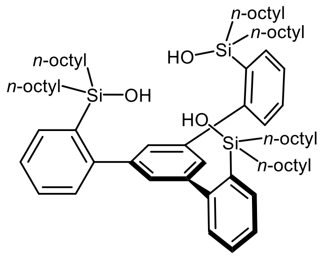

New Complexes

Complex 1f. A 100 mL Schlenk flask was equipped with a magnetic stir bar and flame dried under vacuum. The flask was filled with argon and charged with ligand **3f** (1.23 g, 2.01 mmol), which was azeotropically dried with benzene (3 x 5 mL) to remove residual water. Toluene (28 mL) was added and the mixture vigorously stirred for 10 min to obtain a clear solution. Next, a solution of complex **4a** (925 mg, 2.14 mmol)[4] in toluene (15 mL) was added dropwise and stirring was continued for 4 h at ambient temperature. The solvent was removed *in vacuo* and the crude solid was extracted with *n*-pentane (5 x 15 mL) to give a yellow/orange powder containing only the monomeric complex **1f** (1.63 g, 99%); this sample was ca. 97% pure according to NMR. ^{1}H NMR (600 MHz, [D$_8$]-toluene): δ = 7.44 (s, 3H), 7.43 – 7.39 (m, 3H), 7.26 – 7.21 (m, 3H), 7.20 – 7.14 (m, 6H), 6.80 – 6.76 (m, 2H), 6.65 (t, *J* = 7.5 Hz, 1H), 2.68 (s, 6H), 1.01 – 0.96 (m, 18H), 0.95 – 0.86 (m, 12H). ^{13}C NMR (151 MHz, [D$_8$]-toluene): δ = 305.8, 149.2, 145.0, 144.2, 137.9, 135.9, 134.2, 130.4, 128.6, 127.6, 127.0, 126.3, 126.2, 20.4, 9.1, 6.8. ^{29}Si NMR (119 MHz, [D$_8$]-toluene): δ = 11.8. ^{95}Mo NMR (26 MHz, 60°C, [D$_8$]-toluene): δ = 416.9 . IR (film): $\tilde{\nu}$ 3051, 2952, 2931, 2909, 2872, 1581, 1557, 1460, 1429, 1407, 1375, 1259, 1232, 1161, 1122, 1088, 1063, 1044, 1012, 1002, 912, 761, 725, 697, 668, 624, 584, 552, 528, 513, 479, 460, 420 cm^{-1}. HRMS-ESI (*m/z*): calculated for $C_{45}H_{54}MoO_3Si_3^+$ [M]$^+$: 824.24293; found, 824.24333. Elemental analysis (%) calcd. for $C_{45}H_{54}MoO_3Si_3$: C 65.66, H 6.61, Mo 11.66, Si 10.24; found: C 64.10, H 6.47, Mo 11.27, Si 9.87 (for the sample that is ca. 97% pure, cf. copies of spectra).

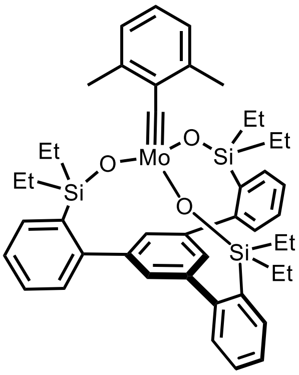

Yellow crystals suitable for single-crystal X-ray diffraction were grown from a concentrated Et_2O solution at –20°C

Complex 1g. A 100 mL Schlenk flask was charged with ligand **3g** (255 mg, 0.33 mmol), which was

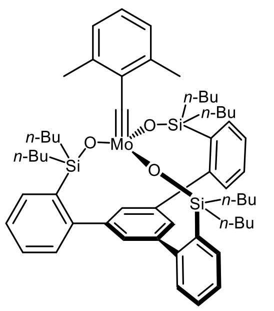

azeotropically dried with benzene (2 × 5 mL) to remove residual water. The ligand was dissolved in toluene (30 mL). A solution of the molybdenum alkylidyne complex **4a** (148 mg, 0.33 mmol)[4] in toluene (15 mL) was added dropwise and stirring of the mixture was continued for 4 h at ambient temperature. The solvent was removed *in vacuo* and the crude material was extracted with *n*-pentane (5 × 15 mL) to give the title complex as a yellow powder. In order to obtain pure material, the crude product was dissolved in *n*-pentane (1 mL) and the complex precipitated by storing the solution at −30 °C for 3 h (169 mg, 58%). ^{1}H NMR (600 MHz, [D$_8$]-toluene) δ 7.52 – 7.49 (m, 6H), 7.28 – 7.22 (m, 3H), 7.22 – 7.16 (m, 6H), 6.77 (d, J = 7.4 Hz, 2H), 6.65 (t, J = 7.5 Hz, 1H), 2.70 (s, 6H), 1.52 – 1.34 (m, 12H), 1.26 (h, J = 7.4 Hz, 12H), 1.06 – 0.95 (m, 12H), 0.72 (t, J = 7.3 Hz, 18H). ^{13}C NMR (151 MHz, [D$_8$]-toluene) δ 306.1, 149.4, 145.4, 144.6, 138.3, 137.2, 134.8, 130.7, 129.1, 128.3, 127.4, 126.6, 126.6, 26.9, 26.0, 20.9, 18.4, 13.7. ^{29}Si NMR (119 MHz, [D$_8$]-toluene) δ 9.9. ^{95}Mo NMR (26 MHz, [D$_8$]-toluene) δ 419.6. IR (film): ṽ 2953, 2920, 2869, 2854, 1461, 1408, 1123, 992, 882, 865,830, 758, 722, 698, 658, 464. HRMS (ESI) calcd. for $C_{57}H_{79}MoO_3Si_3$ [M+H]$^+$: 993.43856; found, 993.43965. Elemental analysis (%) calcd. for $C_{57}H_{78}MoO_3Si_3$: C 69.05, H 7.93, Mo 9.68; found: C 67.00, H 7.75, Mo 9.37.

Complex 1h. A 100 mL Schlenk flask was charged with ligand **3h** (271 mg, 0.35 mmol), which was

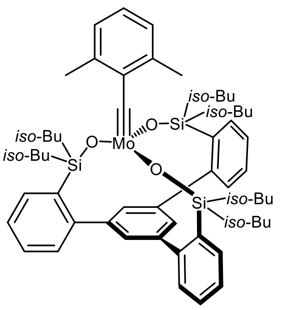

azeotropically dried with benzene (2 × 4 mL) to remove residual water. The ligand was dissolved in toluene (40 mL). A solution of the molybdenum alkylidyne complex **4a** (150 mg, 0.35 mmol)[4] in toluene (7 mL) was added dropwise and stirring of the mixture was continued for 5 h at ambient temperature. The solvent was removed *in vacuo* and the obtained brown residue was extracted with *n*-pentane (6 mL). The solution was concentrated *in vacuo* until 2 mL remained and stored at −78 °C for 2 h. The title complex precipitated as a yellow solid material, which was collected by removal of the supernatant at −78 °C and subsequent drying under high vacuum for 2 h (296 mg, 86%). ^{1}H NMR (600 MHz, [D$_8$]-toluene) δ 7.57 – 7.51 (m, 3H), 7.51 (s, 3H), 7.28 – 7.22 (m, 3H), 7.21 – 7.16 (m, 6H), 6.78 (d, *J* = 7.6 Hz, 2H), 6.65 (t, *J* = 7.6 Hz, 1H), 2.73 (s, 6H), 2.08 – 1.96 (m, *J* = 6.7 Hz, 6H), 1.03 (d, *J* = 6.9 Hz, 12H), 0.89 (d, *J* = 6.6 Hz, 18H), 0.87 (d, *J* = 6.6 Hz, 18H). ^{13}C NMR (151 MHz, [D$_8$]-toluene) δ 306.4, 149.1, 145.4, 144.7, 138.2, 137.7, 135.2, 130.7, 129.1, 127.5, 126.6, 126.5, 30.1, 26.9, 26.6, 24.7, 21.4. ^{29}Si NMR (119 MHz, [D$_8$]-toluene) δ 9.0. ^{95}Mo NMR (26 MHz, [D$_8$]-toluene) δ 432.7. IR (film): *ṽ* 2950, 2865, 1462, 1122, 1089, 1003, 914, 872, 829, 763, 741, 715, 470. HRMS: decomp. Elemental analysis (%) calcd. for $C_{57}H_{78}MoO_3Si_3$: C 69.05, H 7.93, Mo 9.68, Si 8.50; found: C 68.84, H 7.91, Mo 9.64, Si 8.51.

Complex 1i. A 250 mL Schlenk flask was charged with ligand **3i** (405 mg, 0.36 mmol), which was

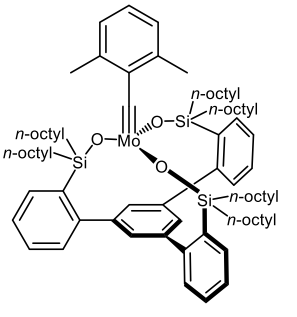

azeotropically dried with benzene (3 × 4 mL) to remove residual water. The ligand was dissolved in toluene (40 mL). A solution of the molybdenum alkylidyne complex **4a** (156 mg, 0.36 mmol)[4] in toluene (10 mL) was added dropwise and stirring of the mixture was continued for 5 h at ambient temperature. The solvent was removed *in vacuo* and the obtained brown residue was extracted with pentane (6 mL). The solution was concentrated *in vacuo* until 2 mL remained and stored at −78 °C for 3 d. The title complex precipitated as a yellow solid material, which was collected by removal of the supernatant at −78 °C and drying of the residue by three freeze-pump-thaw cycles (140 mg, 30%). ^{1}H NMR (600 MHz, [D$_8$]-toluene) δ 7.58 – 7.54 (m, 3H), 7.53 (s, 3H), 7.29 – 7.25 (m, 3H), 7.25 – 7.19 (m,

6H), 6.80 (d, *J* = 7.6 Hz, 2H), 6.67 (t, *J* = 7.6 Hz, 1H), 2.74 (s, 6H), 1.58 – 1.43 (m, 12H), 1.33 – 1.24 (m, 24H), 1.24 – 1.15 (m, 24H), 1.14 – 1.08 (m, 12H), 1.06 (t, *J* = 8.5 Hz, 12H), 0.92 (t, *J* = 7.2 Hz, 18H). ^{13}C NMR (151 MHz, [D$_8$]-toluene) δ 306.1, 149.4, 145.4, 144.6, 138.2, 137.2, 134.8, 130.7, 129.1, 128.3, 127.4, 126.7, 126.6, 34.2, 32.4, 29.9, 29.6, 24.0, 23.2, 21.1, 18.8, 14.4. ^{29}Si NMR (119 MHz, [D$_8$]-toluene) δ 9.7. ^{95}Mo NMR (26 MHz, [D$_8$]-toluene) δ 419.7. IR (film): ṽ 2955, 2920, 2852, 1464, 1122, 991, 885, 863, 832, 761, 738, 721, 703, 662, 461. HRMS: decomp. Elemental analysis (%) calcd. for $C_{81}H_{126}MoO_3Si_3$: C 73.25, H 9.56, Mo 7.23, Si 6.34; found: C 72.17, H 9.56, Mo 7.12, Si 6.51.

Complex 8. A 25 mL Schlenk flask was charged with complex **1a** (40 mg, 50 µmol)[4] and toluene (1.5 mL). 2-Butyne (8.5 µL, 0.11 mmol) was added and the mixture stirred at ambient temperature for 2 h. All volatile materials were removed *in vacuo*. The residue was washed with *n*-pentane (3 × 3 mL) and dried under high vacuum for 5 h gave the title complex as a red solid material (22 mg, 65%). ^{1}H NMR (600 MHz, [D$_8$]-toluene) δ 7.57 – 7.52 (m, 6H), 7.18 (s, 6H), 7.16 – 7.11 (m, 12H), 7.05 – 7.03 (m, 6H), 0.61 (s, 36H); ^{13}C NMR (151 MHz, [D$_8$]-toluene) δ 148.2, 143.7, 139.5, 134.9, 129.7, 129.3, 127.9, 126.9, 5.1; ^{29}Si NMR (119 MHz, [D$_8$]-toluene) δ 9.1; ^{95}Mo NMR (26 MHz, [D$_8$]-toluene) δ 2631.5; IR (film): ṽ 2967, 2906, 1408, 1248, 1126, 1091, 1069, 1028, 925, 823, 803, 783, 762, 739, 722, 693, 666, 641, 453; HRMS (ESI) calculated for $C_{60}H_{66}Mo_2O_6Si_6$ [M]$^+$: 1246.15777; found: 1246.15948; Elemental analysis (%) calculated for $C_{60}H_{66}Mo_2O_6Si_6$: C 57.94, H 5.35, Mo 15.43, Si 13.55; found: C 57.90, H 6.27, Mo 15.47, Si 13.41.

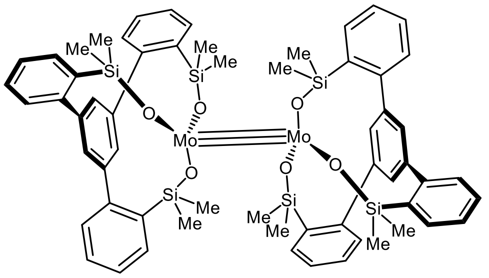

Complex 6.[3] A 500 mL Schlenk flask was equipped with a magnetic stir bar and was flame dried under vacuum. The flask was filled with argon and charged with ligand **11** (1.03 g, 1.98 mmol), which was azeotropically dried with benzene (3 x 5 mL) to remove any residual water. Toluene (148 mL) was added and the mixture was vigorously stirred for 10 min to obtain a clear solution. A solution of complex **7b** (1.03 g, 1.98 mmol) in toluene (30 mL) was then added dropwise to the vigorously stirred mixture. After stirring for 2 h at ambient temperature, the solvent was removed in vacuo to give the title complex as an orange powder (1.62 g, quant.). For the concentration-dependent equilibration with the cyclotetrameric complex **12**, see the copies of the pertinent NMR spectra. The monomeric complex analyzed as follows: ^{1}H NMR (600 MHz, [D$_8$]-toluene): δ 7.30 (dd, *J* = 7.4, 1.7 Hz, 3H), 7.29 (s, 3H), 7.15 (td, *J* = 7.4, 1.6 Hz, 3H), 7.12 (td, *J* = 7.3, 1.7 Hz, 3H), 6.99 (d, *J* = 7.5 Hz, 2H), 6.93 (dd, *J* = 7.4, 1.6 Hz, 3H), 6.65 (t, *J* = 7.5 Hz, 1H), 3.07 (s, 6H), 2.73 (s, 6H), 1.30 (s, 18H). ^{13}C NMR (151 MHz, [D$_8$]-toluene): δ = 264.0 (^1J-^{183}W-^{13}C = 292.7 Hz), 145.6 (^2J-^{183}W-^{13}C = 44.2 Hz), 144.5, 143.3, 139.5, 136.4, 133.4, 132.6, 128.3, 127.1, 127.0, 126.8, 125.2, 83.9, 49.3, 31.4, 22.1. ^{183}W NMR (17 MHz, [D$_8$]-toluene): δ = 114.2. IR (film): ṽ 3032, 2973, 2922, 1458, 1478, 1363, 1377, 1207, 1227, 1170, 1124, 1098, 972, 984, 999, 940, 896, 872, 786, 814, 757, 739, 675, 624, 639, 572, 590, 535, 558, 512, 472, 415 cm^{-1}. HRMS: decomp.; Elemental analysis (%) calcd. for $C_{45}H_{48}O_3W$: C 65.86, H 5.90, W 22.40; found C 65.63, H 5.93, W 22.19.

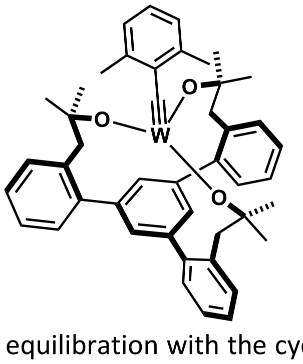

Complex 1e.[4] A 50 mL Schlenk flask was equipped with a magnetic stir bar and flame dried under vacuum. The flask was filled with argon and charged with ligand **3e** (329 mg, 0.304 mmol), which was azeotropically dried with benzene (3 x 5 mL) to remove residual water. Toluene (23 mL) was added and the resulting mixture vigorously stirred for 10 min to obtain a clear solution. A solution of ArC≡Mo(O*t*Bu)$_3$ (Ar = *p*-MeOC$_6$H$_4$-, **4b**) (132 mg, 0.304 mmol) in toluene (5 mL) was added dropwise and stirring was continued for 1 h. The solvent was removed *in vacuo* to give a yellow powder (299 mg, 76%) consisting of a mixture of monomer **1e** and oligomer [**1e**]$_n$, which was used in the next step.

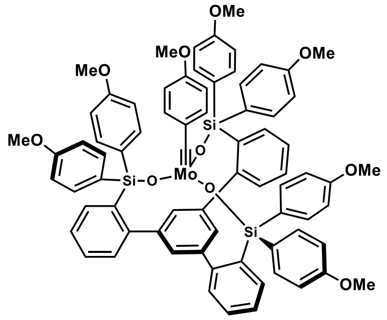

A 10 mL Schlenk flask was equipped with a magnetic stir bar and flame dried under vacuum. The flask was filled with argon and charged with the crude mixture of [**1e**]$_n$/**1e** (60.0 mg, 46.4 µmol) and [D$_8$]-toluene (1 mL). The resulting yellow suspension was vigorously stirred at 60°C for 1 h to give an orange solution containing only monomeric complex **1e** which analyzed as follows: ^{1}H NMR (400 MHz, [D$_8$]-toluene): δ = 7.85 (dd, *J* = 6.9, 1.9 Hz, 3H), 7.81 – 7.73 (m, 9H), 7.36 (s, 3H), 7.15 (dd, *J* = 7.1, 1.8 Hz, 3H), 7.13 – 7.08 (m, 3H), 6.97 – 6.93 (m, 3H), 6.73 – 6.58 (m, 12H), 6.28 – 6.23 (m, 3H), 6.20 – 6.10 (m, 3H), 3.31 (s, 18H), 3.06 (s, 3H). ^{13}C NMR (101 MHz, [D$_8$]-toluene): δ = 309.3, 161.0, 158.5, 149.3, 143.6, 140.7, 136.2, 136.1, 130.2, 130.0, 129.4, 129.1, 128.9, 125.6, 113.6, 112.0, 53.9, 53.9. ^{29}Si NMR (79 MHz, C$_6$D$_6$): δ = −9.1. ^{95}Mo NMR (26 MHz, 60°C, [D$_8$]-toluene): δ = 414.3. IR (film): $\tilde{\nu}$ 2834, 1592, 1563, 1501, 1461, 1439, 1409, 1397, 1277, 1244, 1179, 1113, 1063, 1030, 994, 868, 820, 796, 759, 731, 692, 647, 622, 530, 502, 464, 426, 408 cm^{-1}. HRMS-APPI (*m/z*): calculated for $C_{74}H_{64}MoO_{10}Si_3^+$ [M+H]$^+$, 1294.28559; found, 1294.28623. Elemental analysis (%) calculated for $C_{74}H_{64}MoO_{10}Si_3$: C 68.71, H 4.99, Mo 7.42, Si 6.51; found: C 68.37, H 5.12, Mo 7.33, Si 6.41.

Table S3. Measured (DOSY) and predicted diffusion coefficients (*D*) of molybdenum alkylidyne complexes.

Complex	MW (g·mol^{-1})	$D_{predicted}$ [m^2·s^{-1}]	$D_{exp.}$ [m^2·s^{-1}] [a]	Δ
**1e**	1293.48	5.20·10^{-10} ± 1.5·10^{-10}	4.758·10^{-10}	−8.47%
[**1e**]$_2$	2586.97	3.86·10^{-10} ± 1.1·10^{-10}	2.959·10^{-10}	−23.36%
[**1e**]$_3$	3880.45	3.27·10^{-10} ± 1.1·10^{-10}	2.959·10^{-10}	−9.42%
[**1e**]$_4$	5173.92	2.91·10^{-10} ± 1.1·10^{-10}	2.959·10^{-10}	−1.73%

As can be seen from Table S3, the best match between the recorded and the predicted data is reached for a supramolecular tetramer; it cannot be excluded, however, that the recorded data average over different aggregation states present in solution

The HRMS (ESI) spectra recorded from solutions of the complex in either THF, MeCN or toluene show only the monomeric unit; this result is taken as an additional indication that the complex [**1e**]$_n$ is a supramolecular aggregate rather than a covalently linked entity.

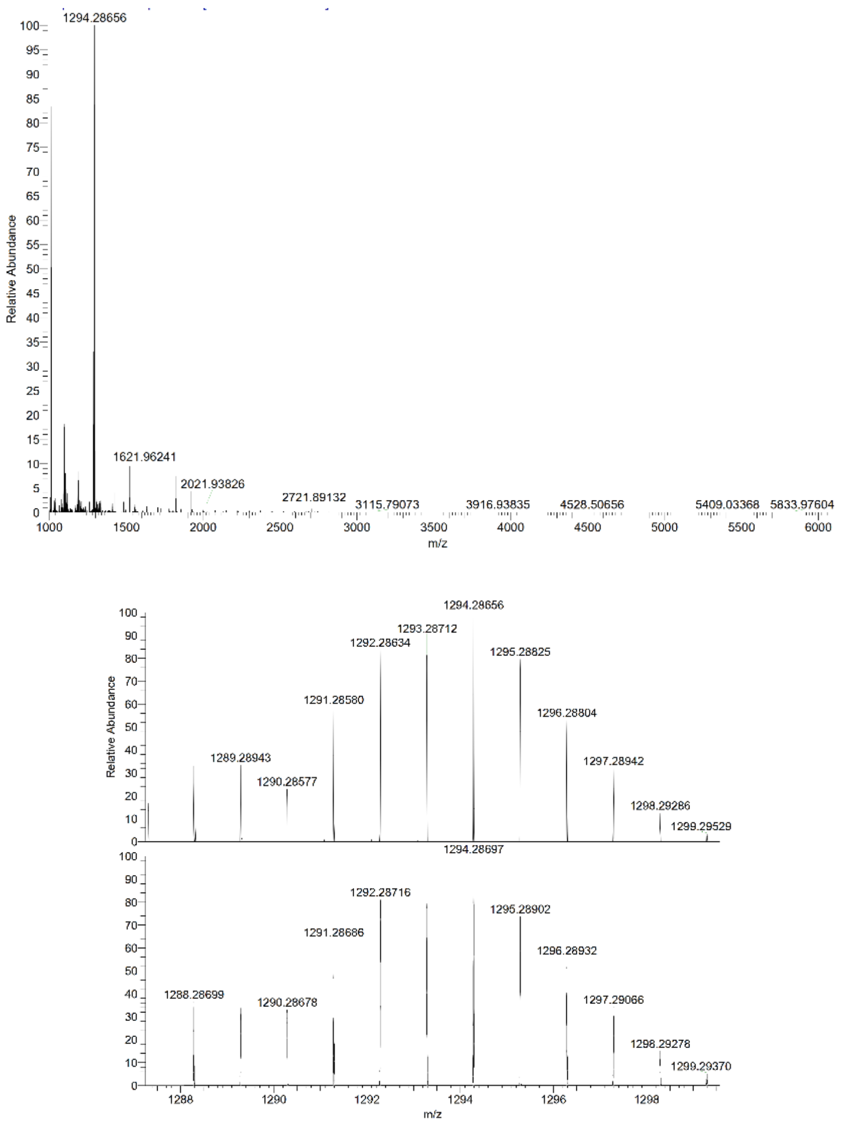

Figure S15. High resolution mass spectrometry ESI spectra of a solution of [**1e**]$_n$ in THF; the spectra correspond to the monomeric unit **1e**

Addition of pyridine (1-4 equiv.) to a solution of [**1e**]$_2$ in [D_8]-toluene afforded the expected adduct [**1e**·pyridine], which, however, could not be isolated in pure form. The spectra show a strong temperature-dependence and 2D EASY-ROESY shows that free and coordinated pyridine do exchange with each other even at low temperatures. The spectra of the adduct recorded at –40°C are sufficiently well resolved to allow for full assignment of all signals and hence confident assignment of the structure.

Table S4. NMR assignment of [**1e**·pyridine] at -40°C in [D_8]-toluene. The sample was prepared by mixing 1 eq of the complex with 4 eq of pyridine. Arbitrary numbering scheme as shown in the Insert

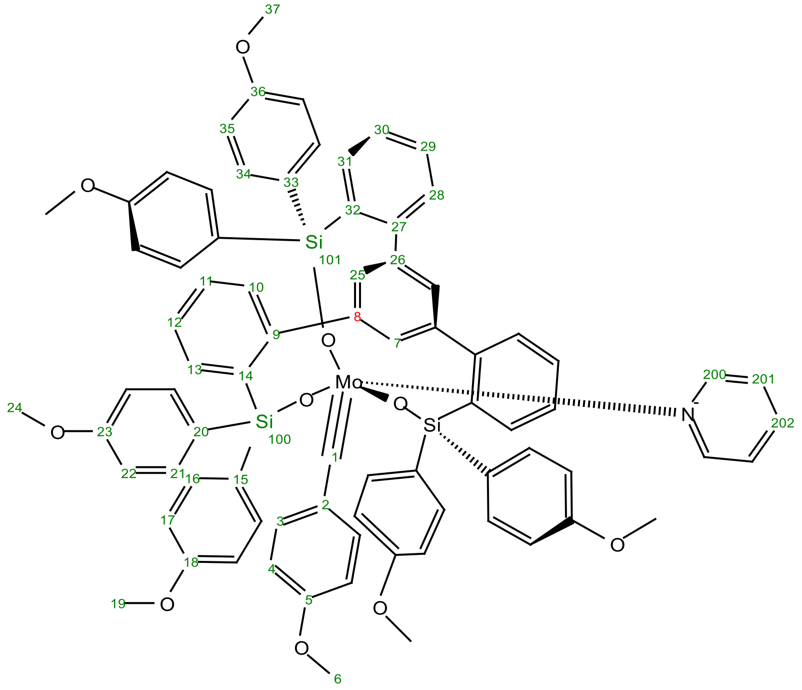

Atom	δ (ppm)	COSY	HSQC	HMBC	NOESY	Atom	δ (ppm)	COSY	HSQC	HMBC	NOESY	Atom	δ (ppm)	COSY	HSQC	HMBC	NOESY
1 C	304.141			3		16 C	137.33		16	16		30 C	126.064		30	28	
2 C	138.627			4		H	8.024	17	16	16, 18, 100	17	H	7.283	29, 31	30	28, 32	31
3 C	131.846		3	3		17 C	113.292		17	17		31 C	135.983		31	29	
H	6.665	4	3	1, 3, 5		H	6.663	16	17	15, 17, 18	16, 19	H	8.054	30	31	27, 29, 32, 101	25, 30
4 C	112.426		4	4		18 C	160.75			16, 17, 19		32 C	139.69			28, 30, 31	
H	6.335	3	4	2, 4, 5	6	19 C	54.118		19			33 C	130.579			35	
5 C	158.754			3, 4, 6		H3	3.277		19	18	17	34 C	137.46		34	34	
6 C	54.288		6			20 C	130.579			22		H	8.047	35	34	34, 35, 36, 101	35
H3	3.156		6	5	4	21 C	137.173		21			35 C	113.371		35	34, 35	
7 C	127.43		7	25		H	6.866	22	21			H	6.465	34	35	33, 35, 36	34, 37
H	7.999	25	7	9, 25	10	22 C	113.069		22	22		36 C	160.607			34, 35, 37	
8 C						H	6.379	21	22	20, 22, 23	24	37 C	54.005		37		
9 C	149.758			7, 11, 25		23 C	160.331			22, 24		H3	3.171		37	36	35
10 C	131.208		10	12		24 C	54.139		24			100 Si	-17.024			16	
H	7.319	11	10		7, 25	H3	3.209		24	23	22	101 Si	-14.833			31, 34	
11 C	129.42		11			25 C	130.553		25	7, 25		200 C	151.77			202	
H	7.192	10	11	9, 13		H	7.596	7	25	7, 9, 25	10, 28, 31	H	8.134				
12 C	125.83		12			26 C	146.57			28		201 C	123.69		201		
H	7.098	12, 13	12	10, 14	13	27 C	149.999			29, 31		H	5.868	202	201		
13 C	137.63		13	11		28 C	128.47		28	30		202 C	136.67		202		
H	7.799	12	13		12	H	7.155	29	28	26, 30, 32	25	H	6.407	201	202	200	
14 C	137.6			12		29 C	129.183		29	31							
15 C	130.38			17		H	7.224	28, 30	29	27, 31							

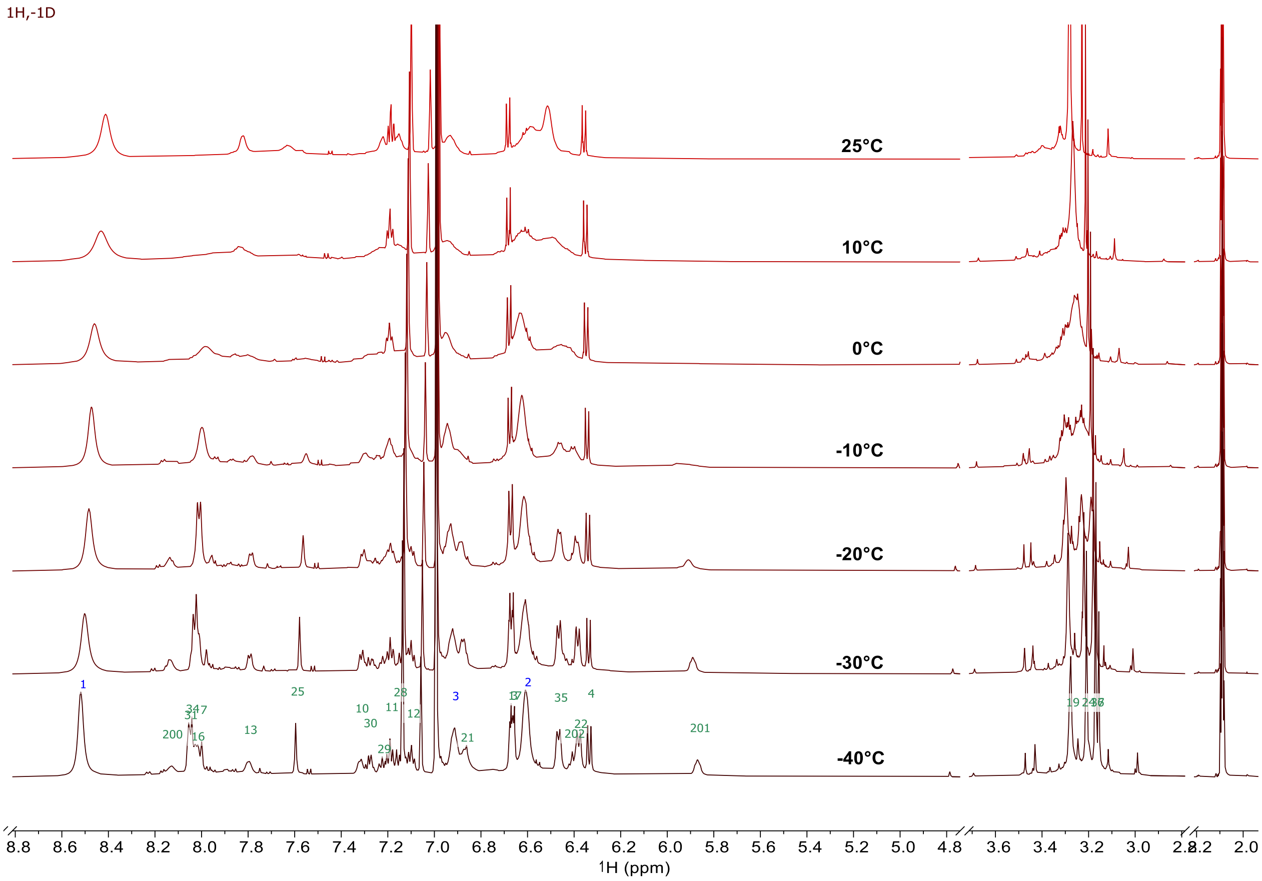

Figure S16. ^{1}H NMR spectra at different temperatures from 25°C to –40°C. The broadening of the signals at higher temperatures is caused by exchange between the adduct [**1e**·pyridine] and free pyridine.

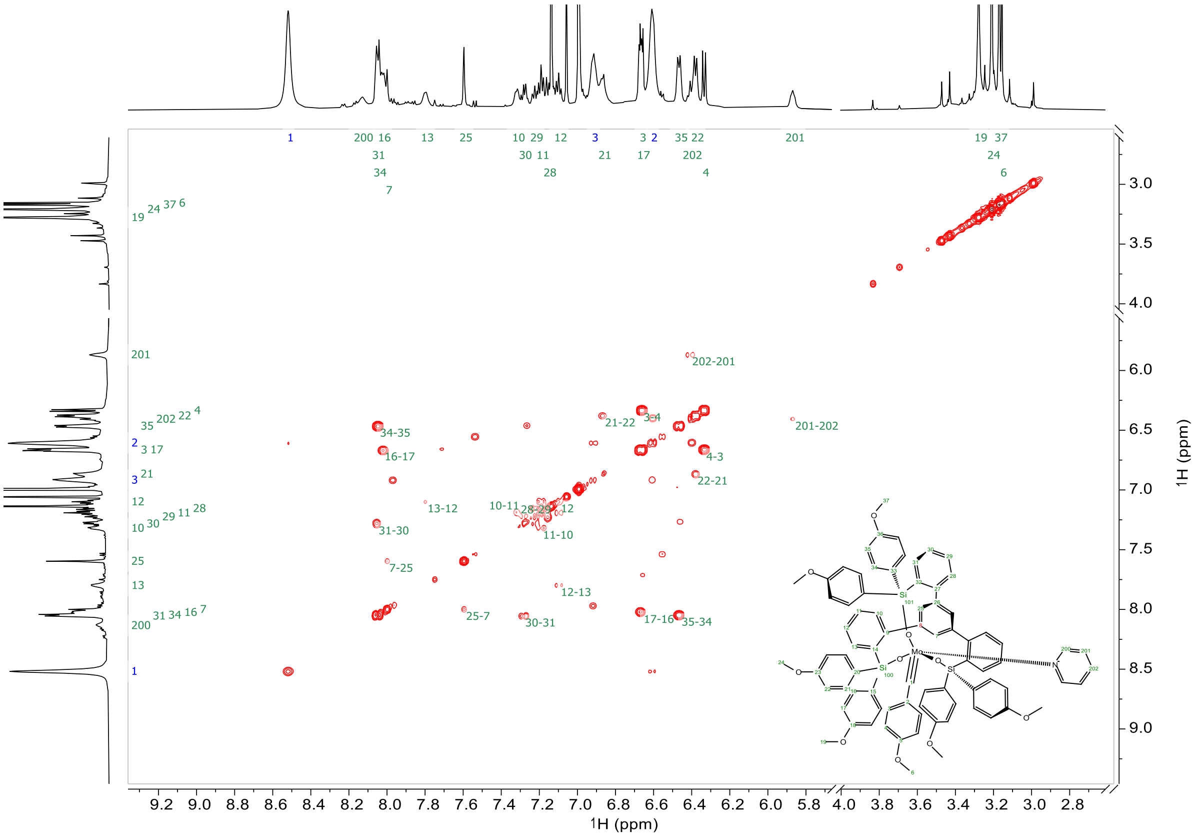

Figure S17. 2D COSY spectrum of [**1e**·pyridine] at –40°C in [D_8]-toluene

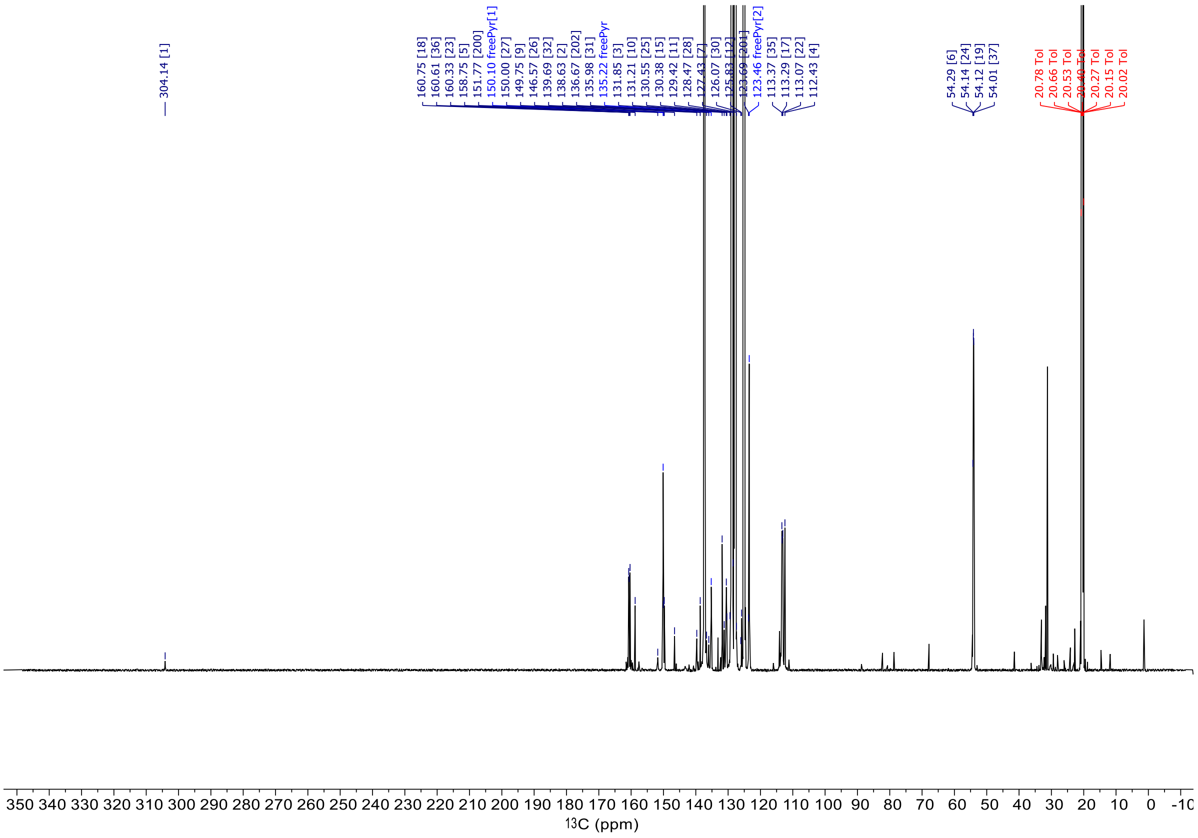

Figure S18. ^{13}C NMR spectrum of [**1e**·pyridine] at –40°C in [D_8]-toluene

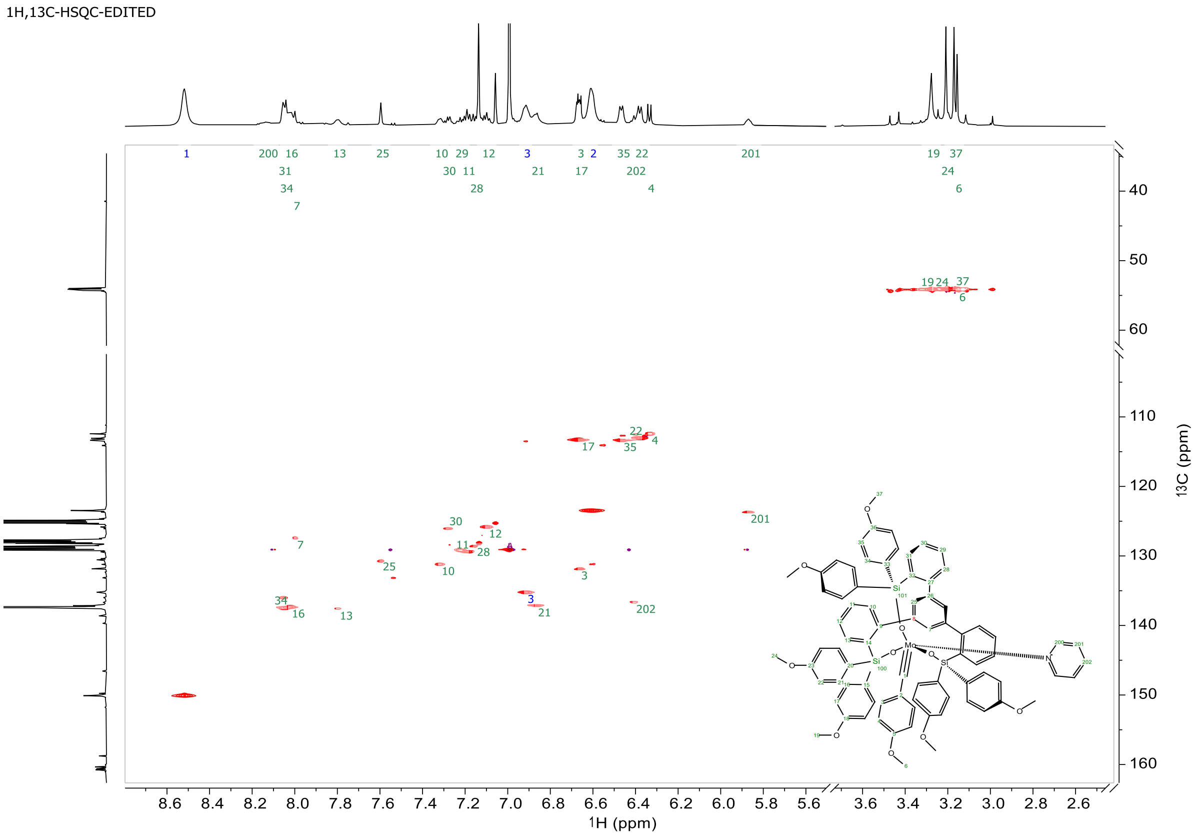

Figure S19. 2D edited ^{1}H-^{13}C-HSQC of [**1e**·pyridine] at –40°C in [D$_8$]-toluene.

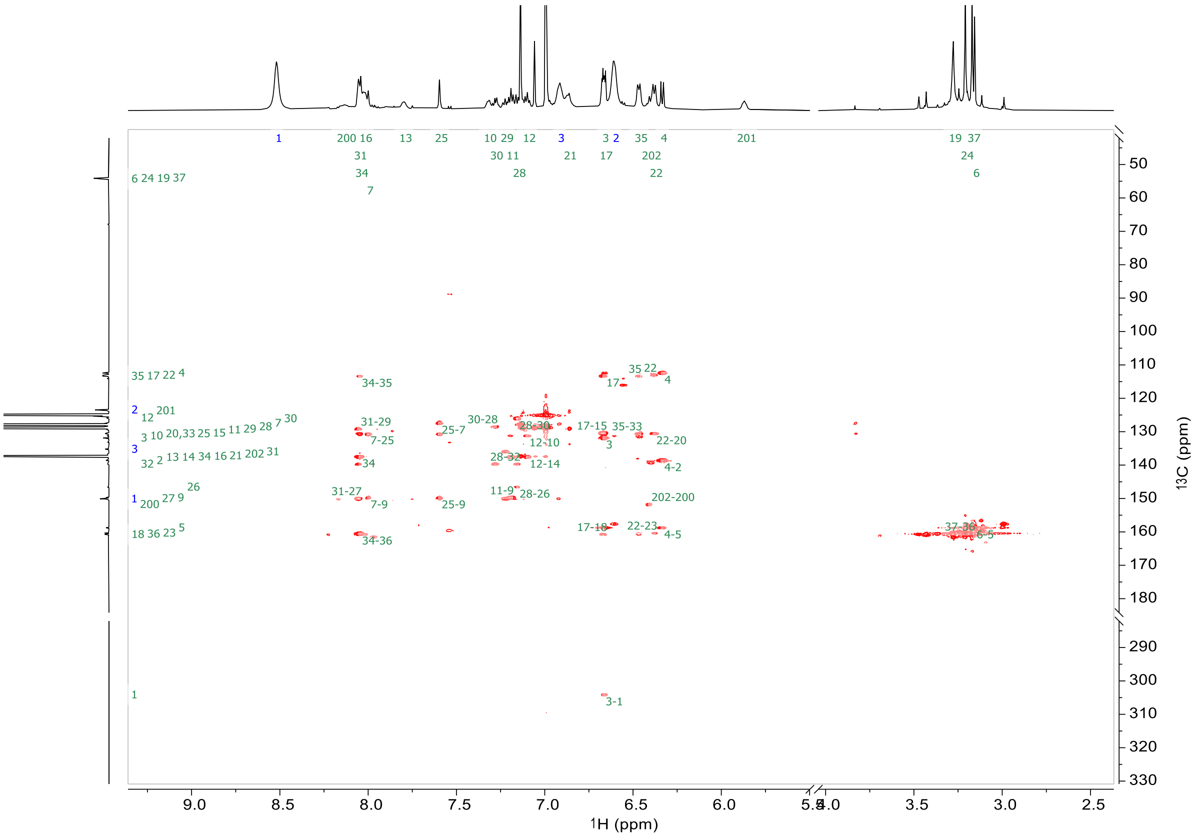

Figure S20. 2D ^{1}H-^{13}C-HMBC of [**1e**·pyridine] at –40°C in [D$_8$]-toluene.

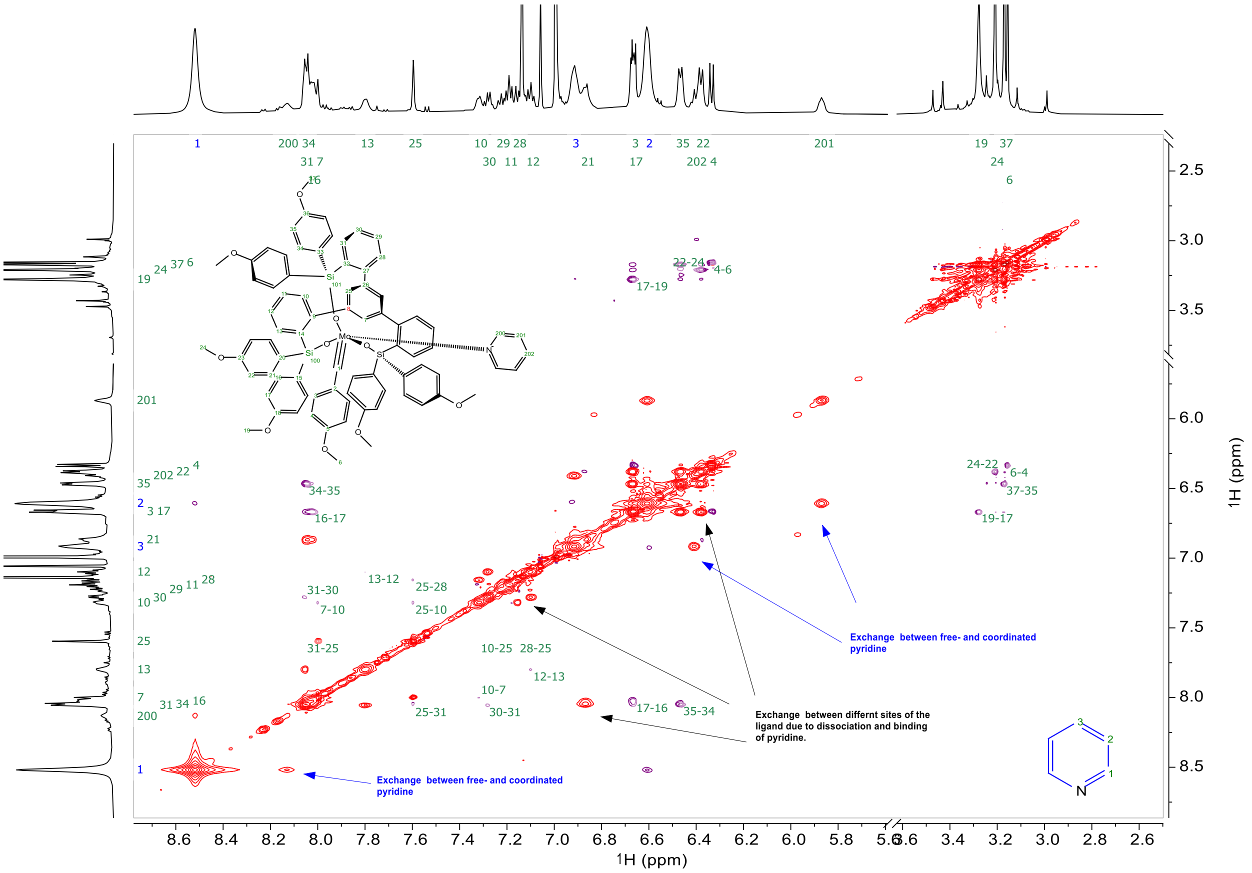

Figure S21. 2D EASY-ROESY spectrum of [**1e**·pyridine] at –40°C in [D_8]-toluene. Blue cross peaks give distance information (due to ROE) and the red EXSY cross peaks give information about chemical exchange of different species.

Complex 10. A 10 mL Schlenk flask was equipped with a magnetic stir bar and flame dried under vacuum. The flask was filled with argon and charged with [**1e**]$_n$ (36.0 mg, 27.8 µmol) and pyridine (3 mL) to give a purple solution. The mixture was vigorously stirred for 1 h at ambient temperature before the solvent was removed *in vacuo* to give the title complex as a purple solid (quant.). ^{1}H NMR (400 MHz, [D_8]-toluene): δ = broad and fairly featureless signals, see the attached copy. IR (film): $\tilde{\nu}$ 1591, 1562, 1500, 1438, 1243, 1275, 1179, 1107, 1030, 987, 886, 822, 796, 757, 719, 689, 649, 625, 500, 532, 460 cm^{-1}. HRMS-ESI (*m/z*): calculated for $C_{296}H_{258}Mo_4O_{40}Si_{12}^+$ [M–4·(C_5H_5N)]$^+$, 2589.57955; found, 2589.58989. Elemental analysis (%) calculated for $C_{316}H_{276}Mo_4N_4O_{40}Si_{12}$: C 69.13, H 5.07, N 1.02, Mo 6.99, Si 6.14; found: C 68.71, H 4.92, N 0.98, Mo 6.91, Si 6.01.

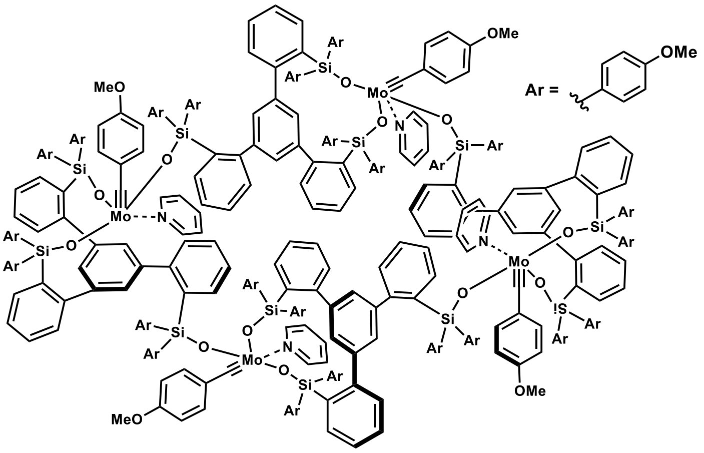

Purple/violet crystals suitable for single-crystal X-ray diffraction were grown from a solution of [**1e**]$_n$ (18.0 mg, 13.9 µmol) in pyridine (2.5 mL) at ambient temperatures that was layered with *n*-pentane).

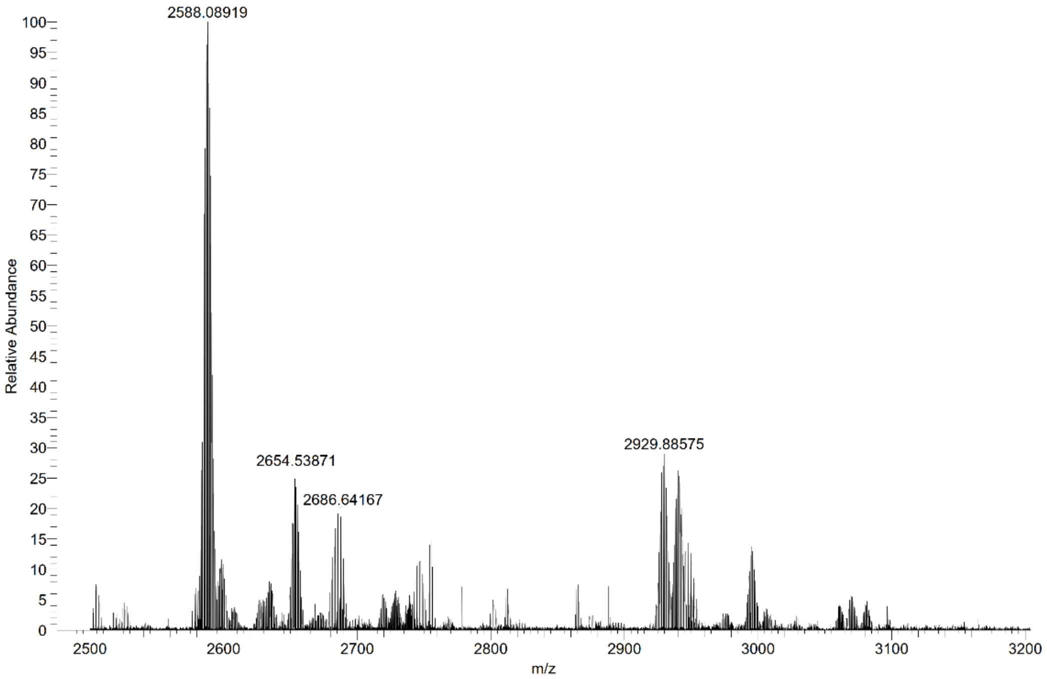

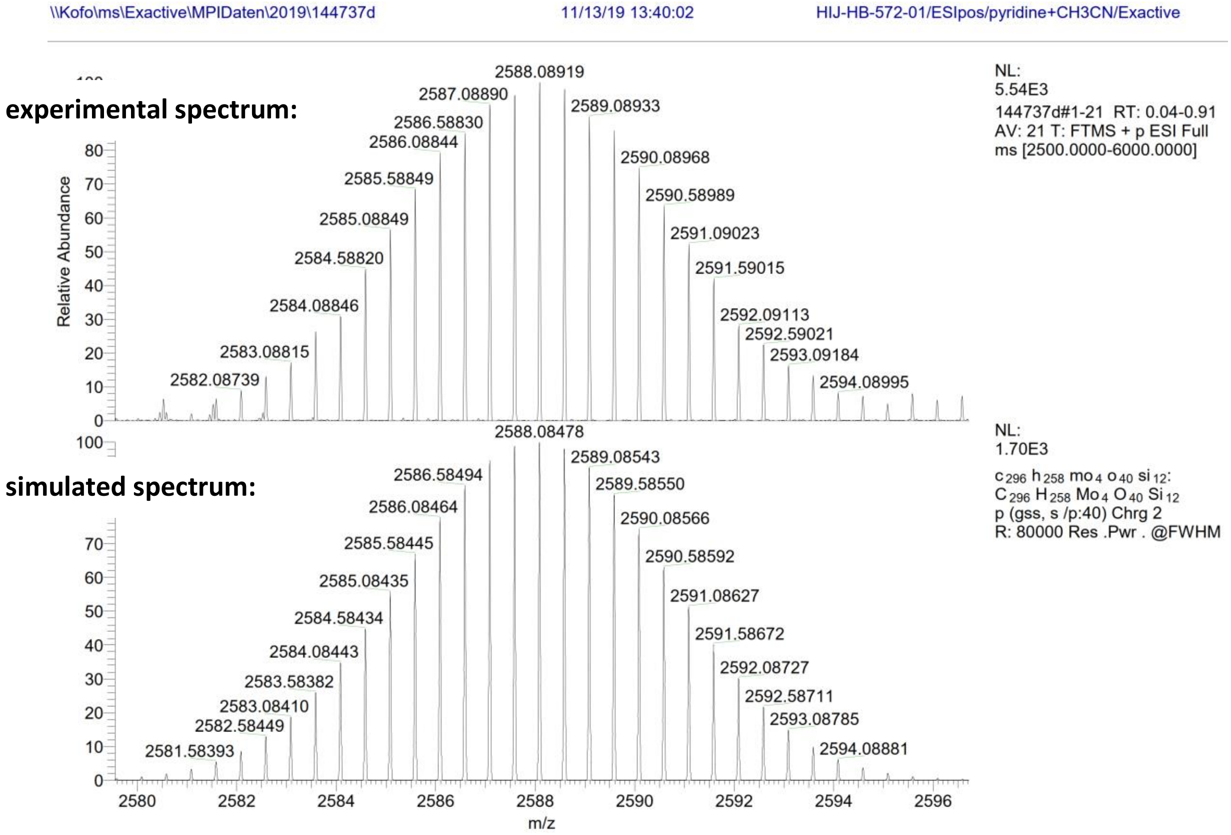

Figure S22. High resolution mass spectra (ESI) of the tetrameric adduct **10** ($C_{296}H_{258}Mo_4O_4Si_{12}$); the data correspond to the di-cationic species (2589 = $[5493 - 4{\cdot}(py) + 2{\cdot}H]^{2+}$) and hence represent the tetrameric structure **10** upon loss of the four molecules of pyridine coordinated to the four molybdenum centers

^{1}H NMR of Silane S1, 400 MHz, $CDCl_3$, 25ºC

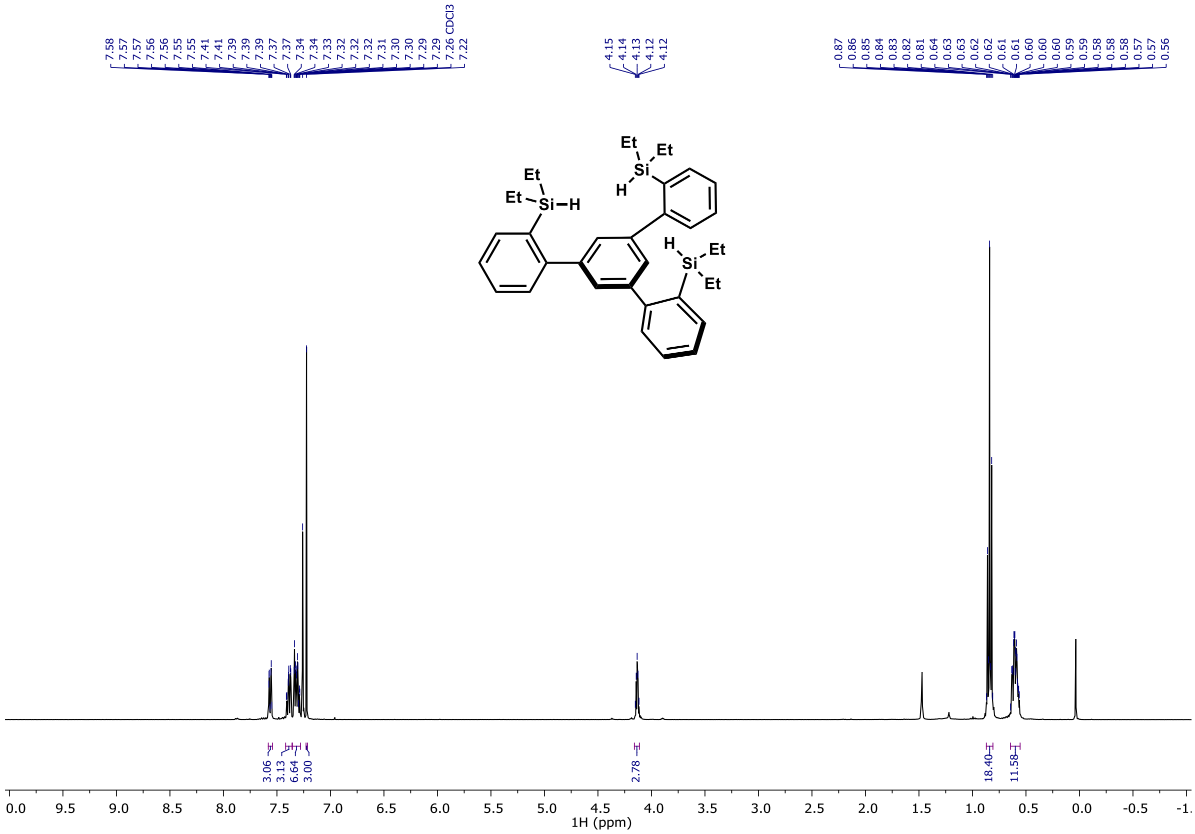

^{13}C NMR of Silane S1, 101 MHz, $CDCl_3$, 25ºC

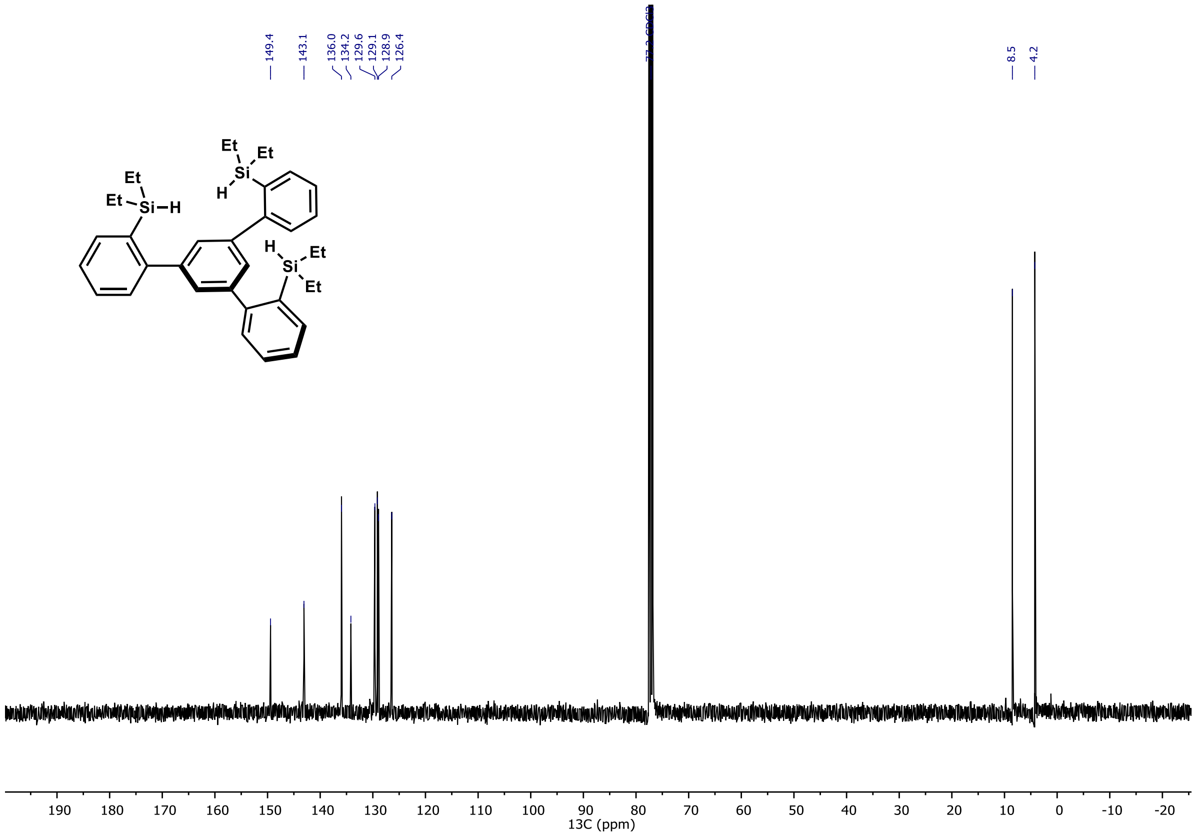

^{1}H NMR of Ligand 3f, 400 MHz, $CDCl_3$, 25°C

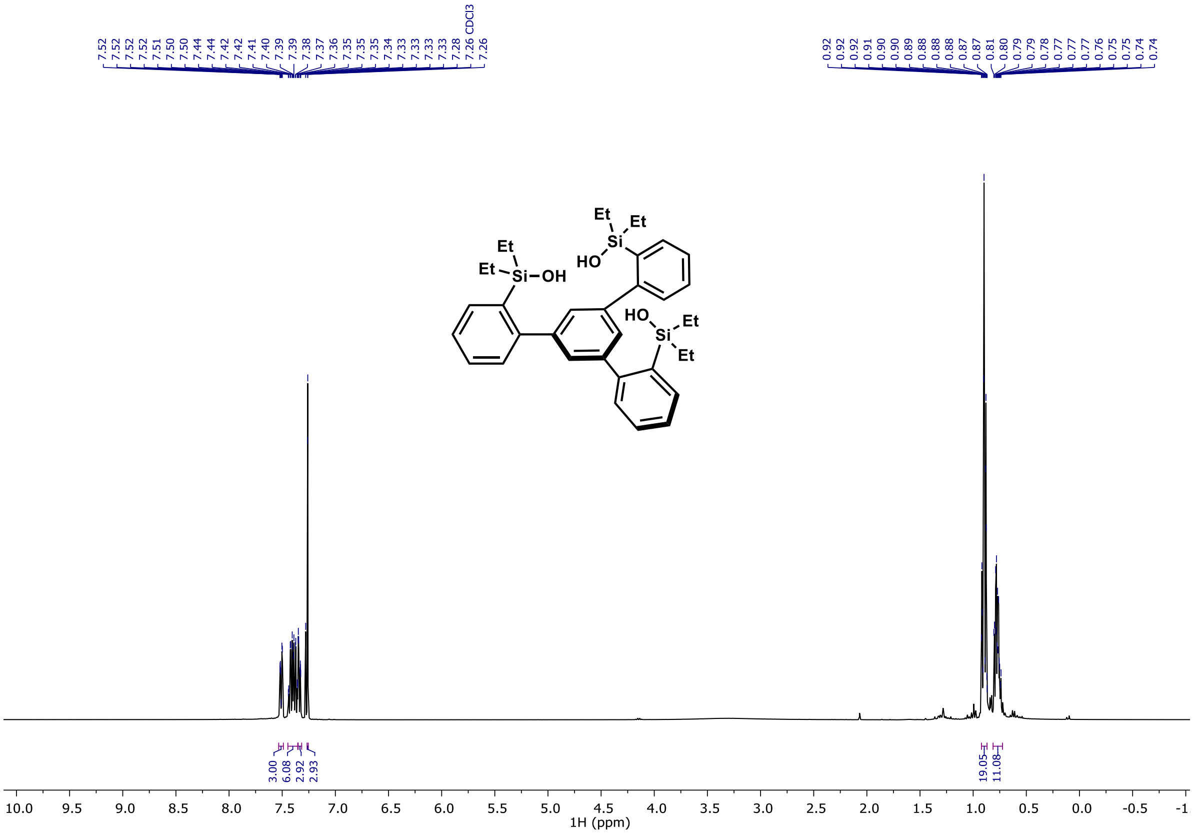

^{13}C NMR of Ligand 3f, 101 MHz, $CDCl_3$, 25°C

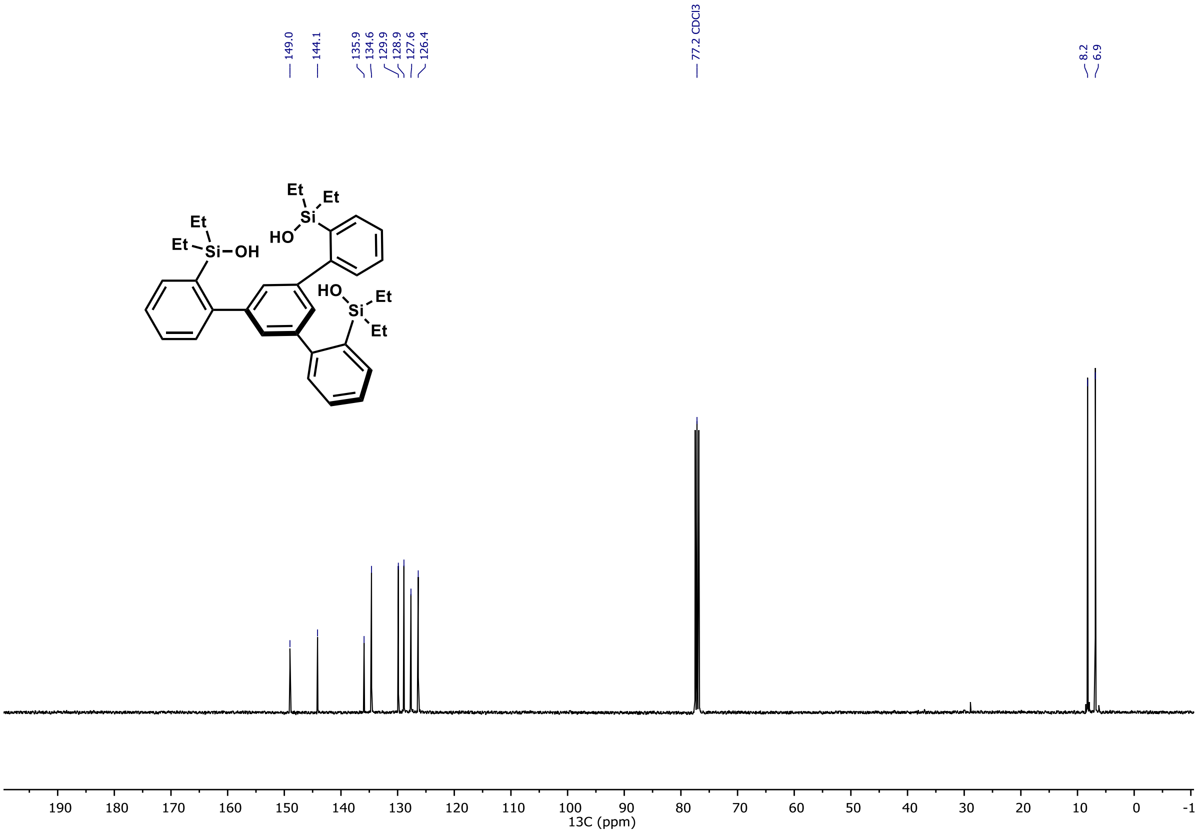

^{29}Si NMR of Ligand 3f, 119 MHz, $CDCl_3$, 25°C

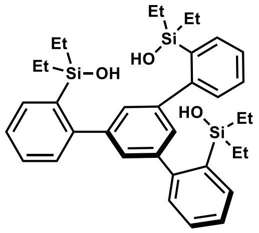

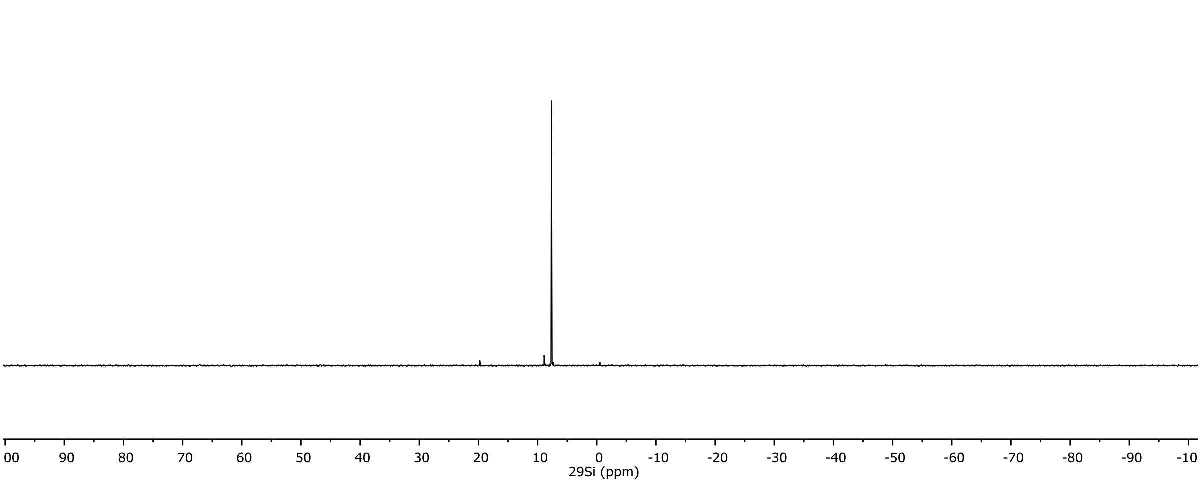

^{1}H NMR of Silane S2, 500 MHz, $CDCl_3$, 25 °C

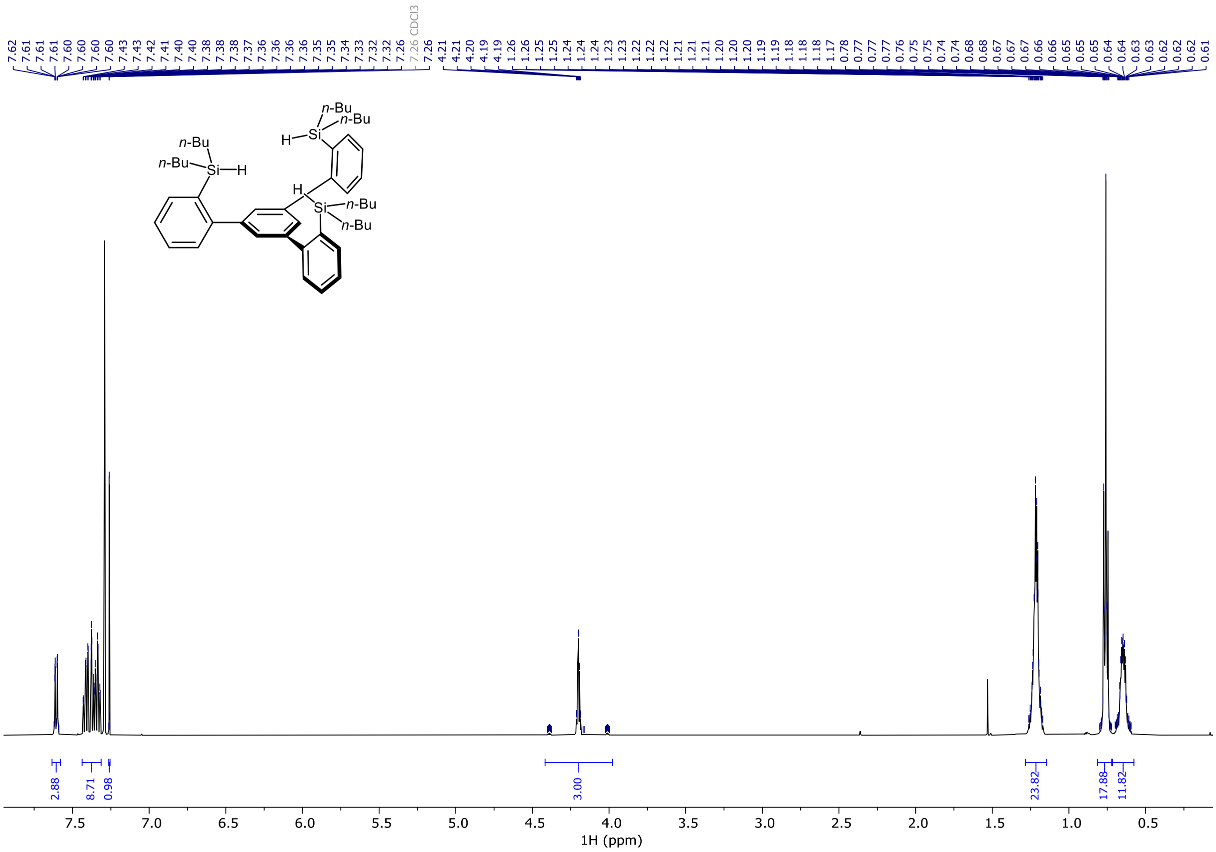

^{13}C NMR of Silane S2, 126 MHz, $CDCl_3$, 25 °C

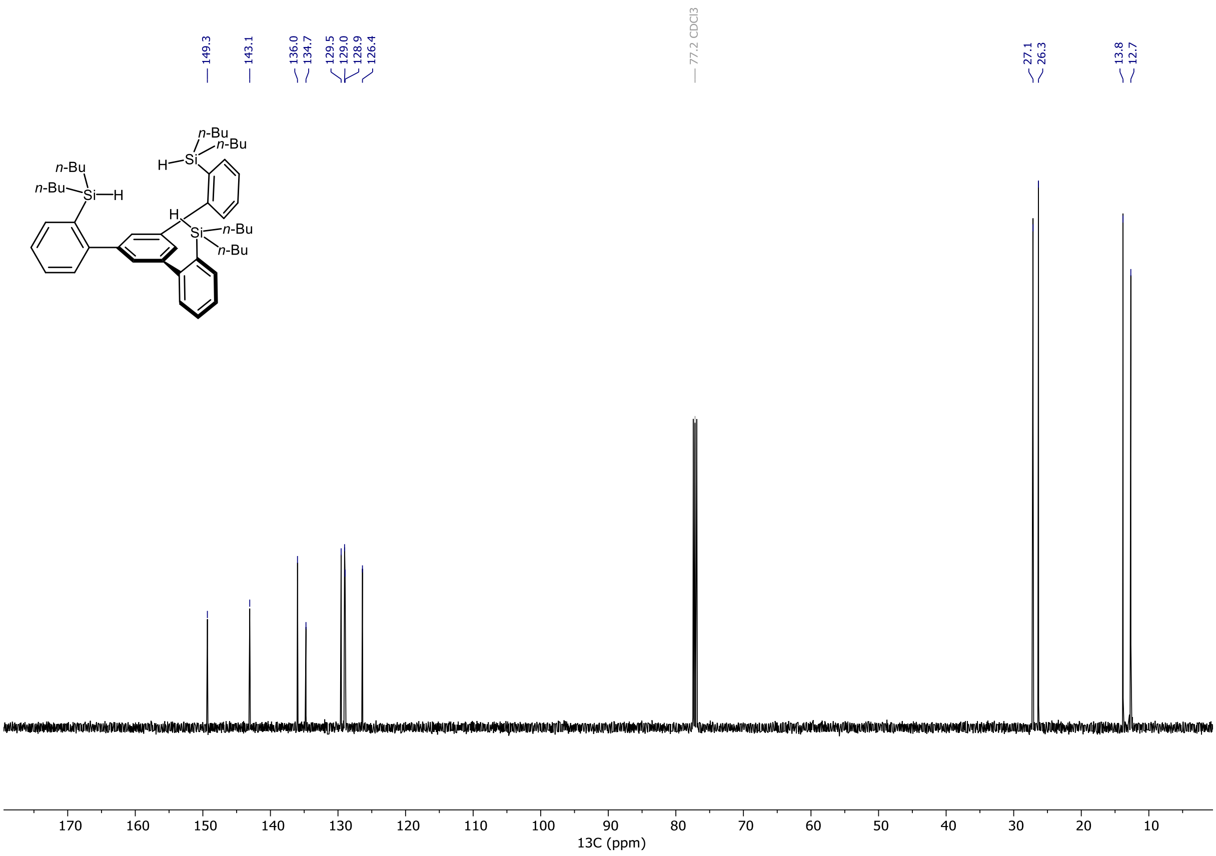

^{29}Si NMR of Silane S2, 99 MHz, $CDCl_3$, 25°C

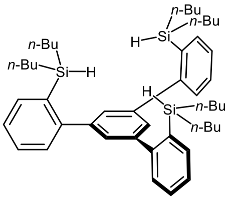

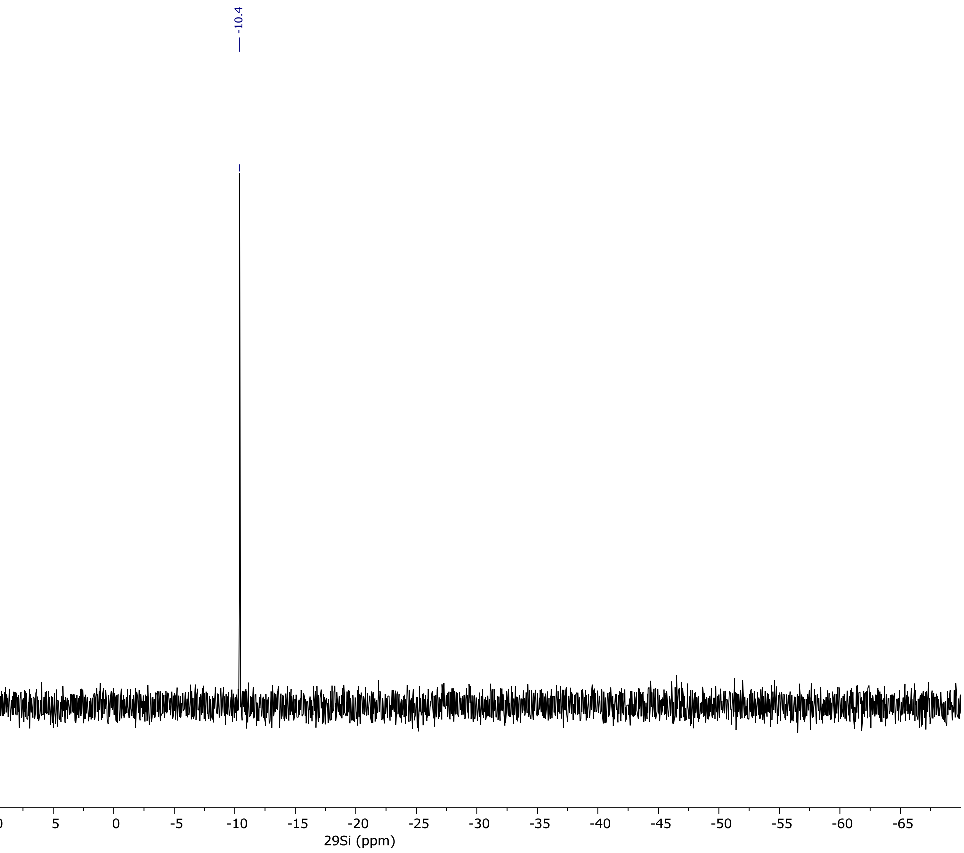

^{1}H NMR of Ligand 3g, 500 MHz, $CDCl_3$, 25°C

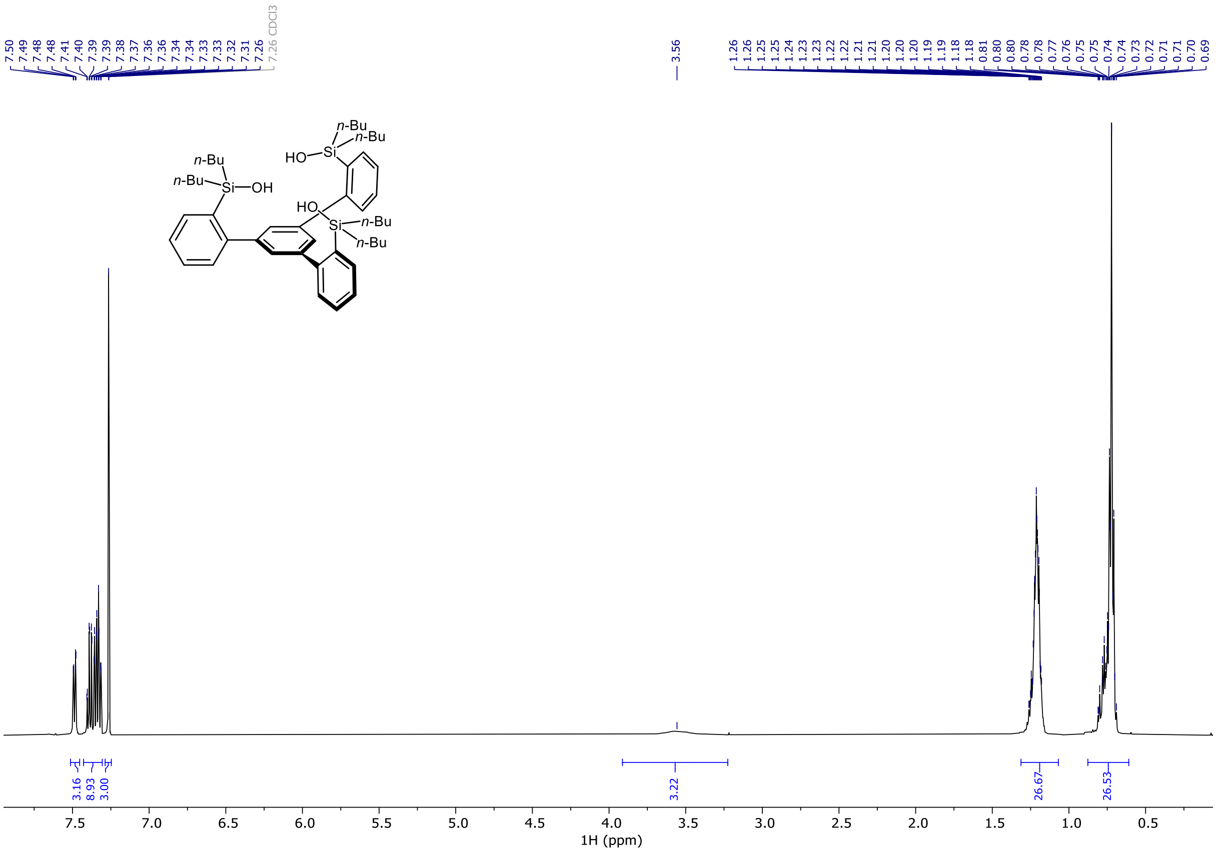

^{13}C NMR of Ligand 3g, 126 MHz, $CDCl_3$, 25°C

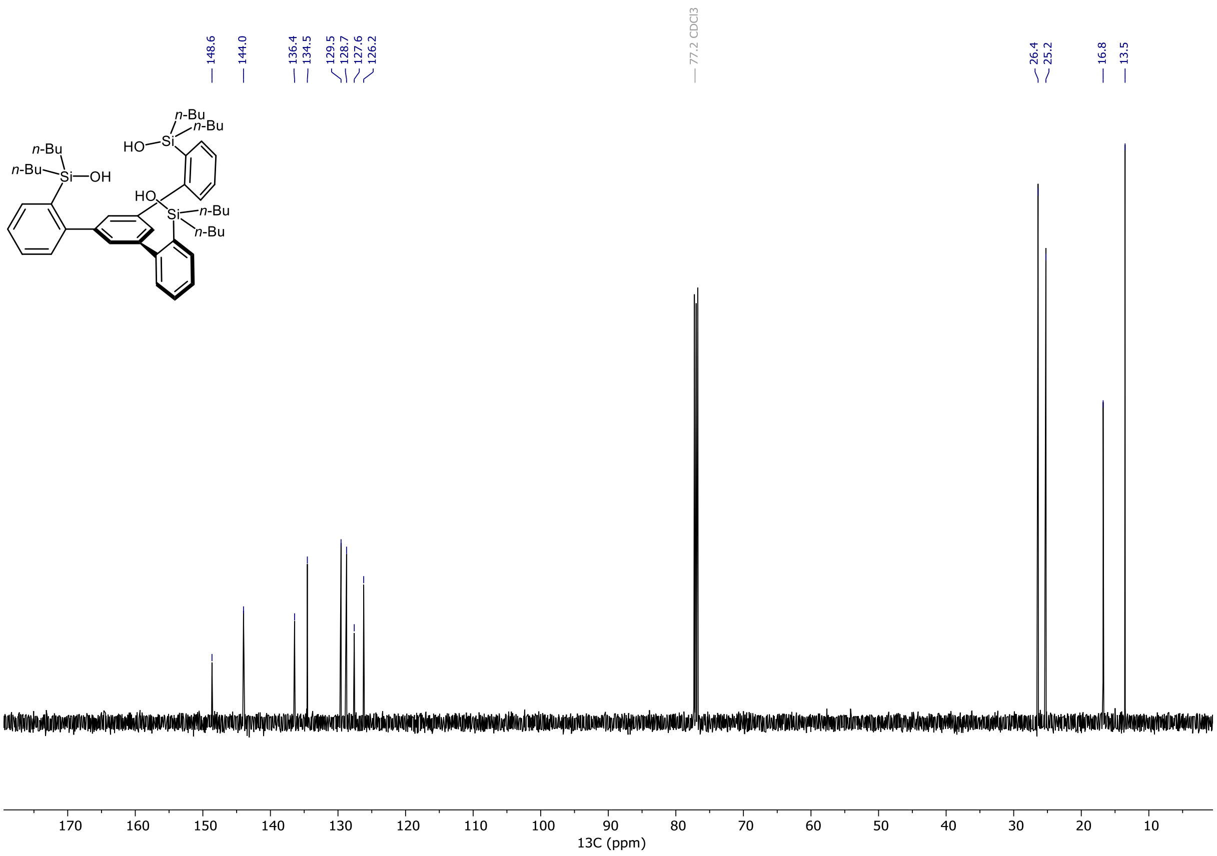

^{29}Si NMR of Ligand 3g, 99 MHz, $CDCl_3$, 25°C

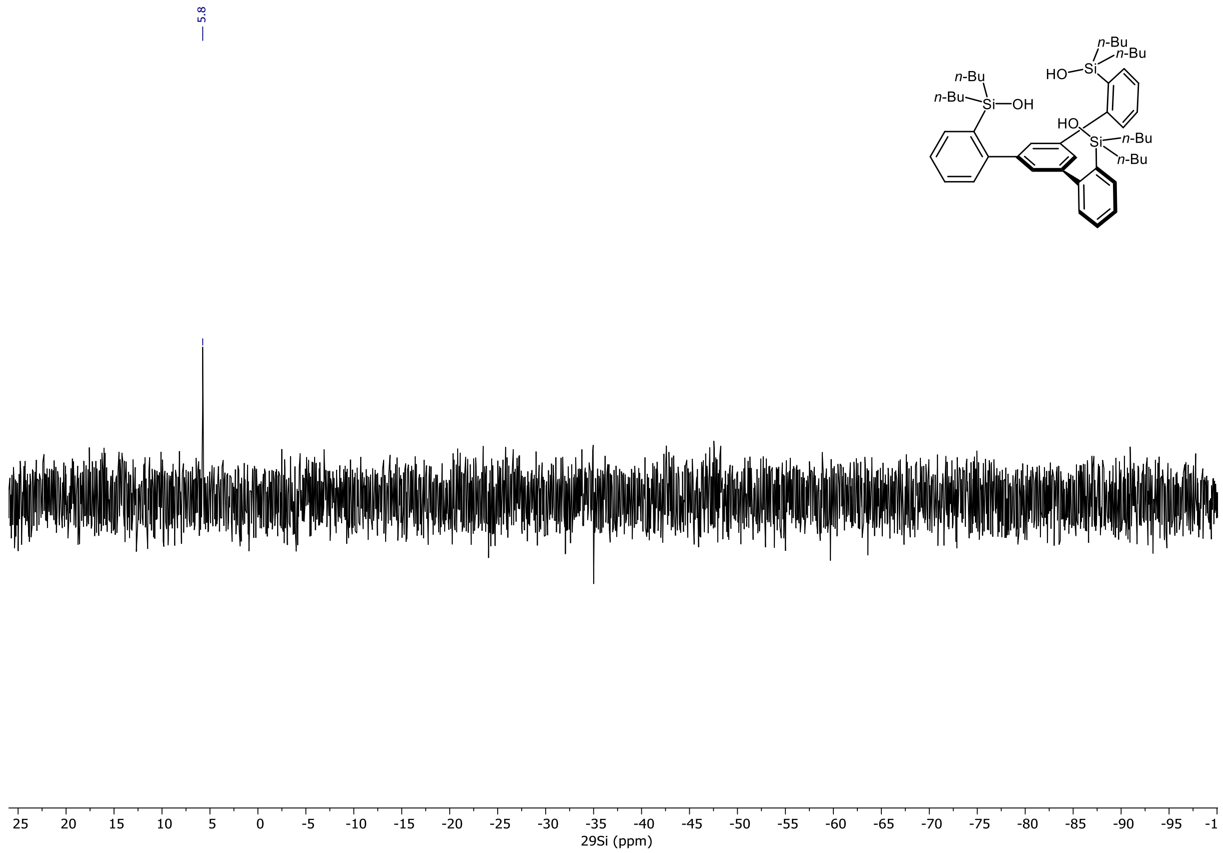

¹H NMR of Silane S3, 600 MHz, $CDCl_3$, 25ºC

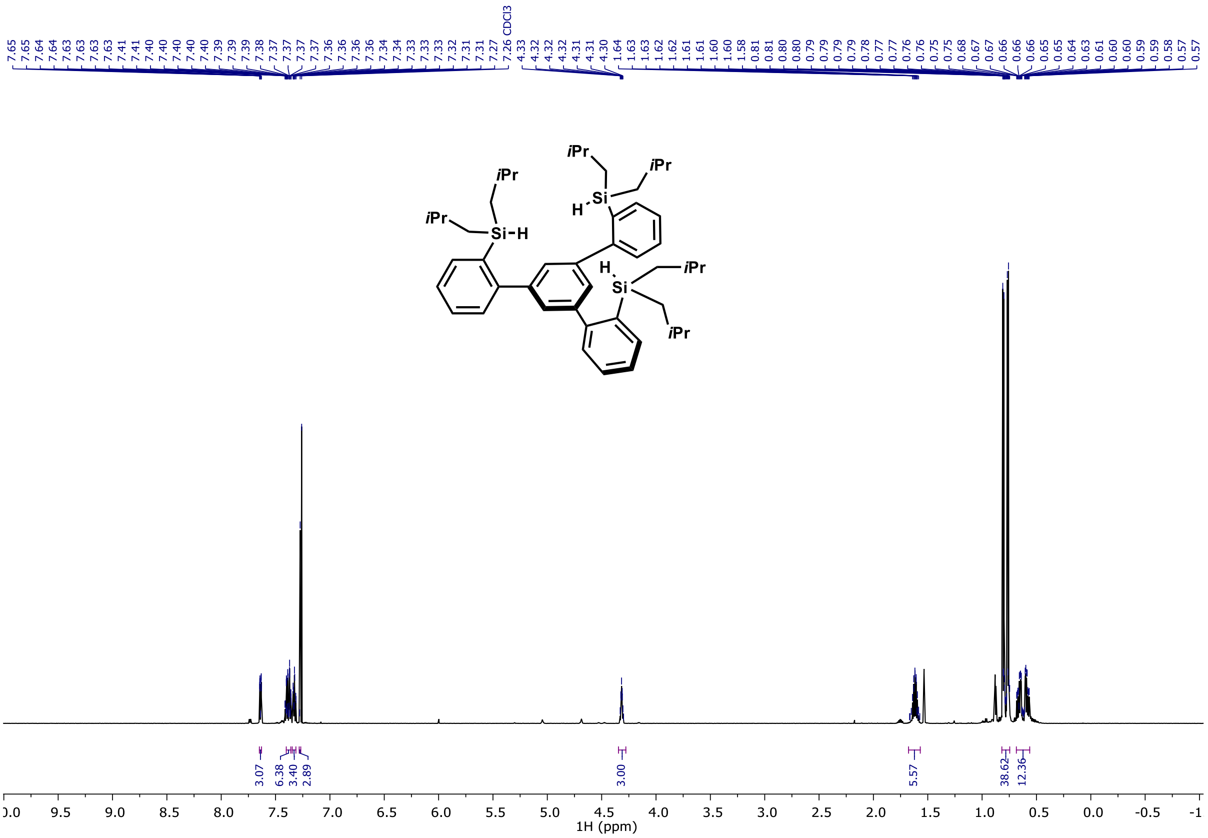

¹³C NMR of Silane S3, 151 MHz, $CDCl_3$, 25ºC

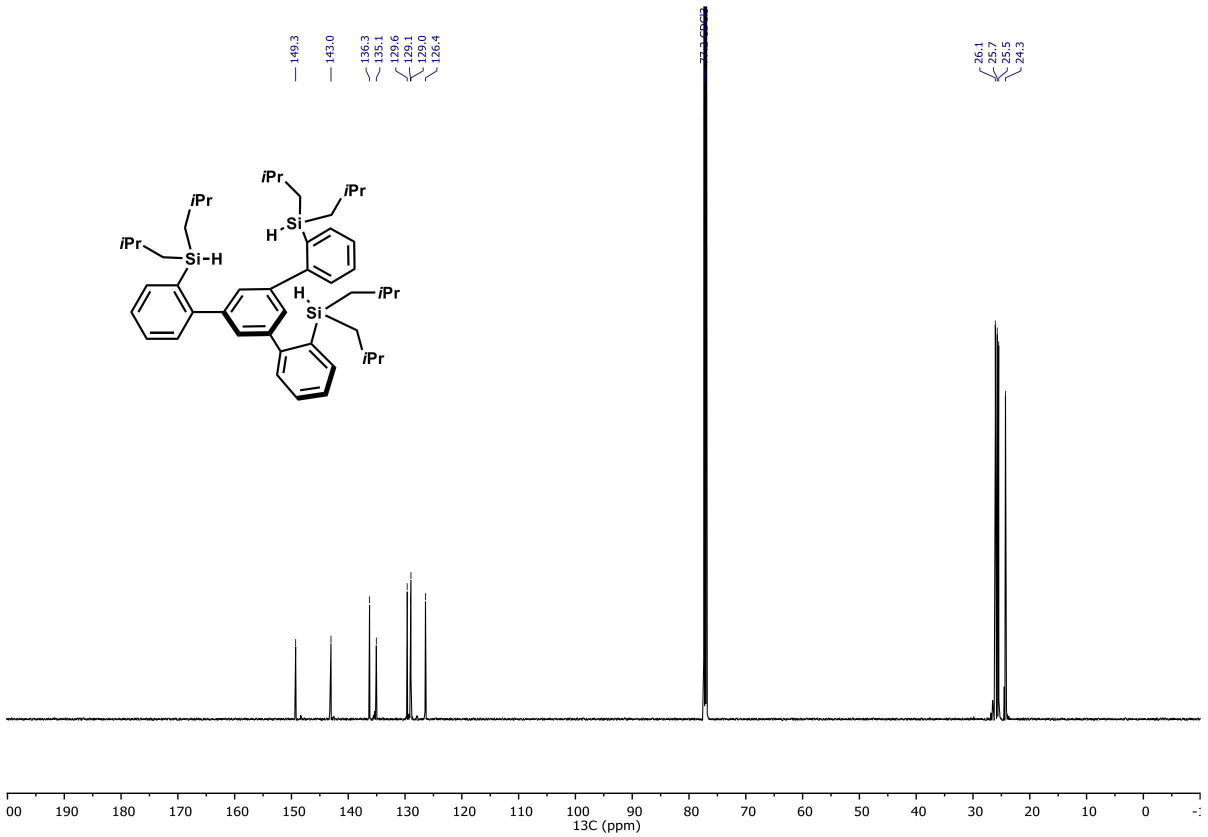

29Si NMR of Silane S3, 119 MHz, $CDCl_3$, 25ºC

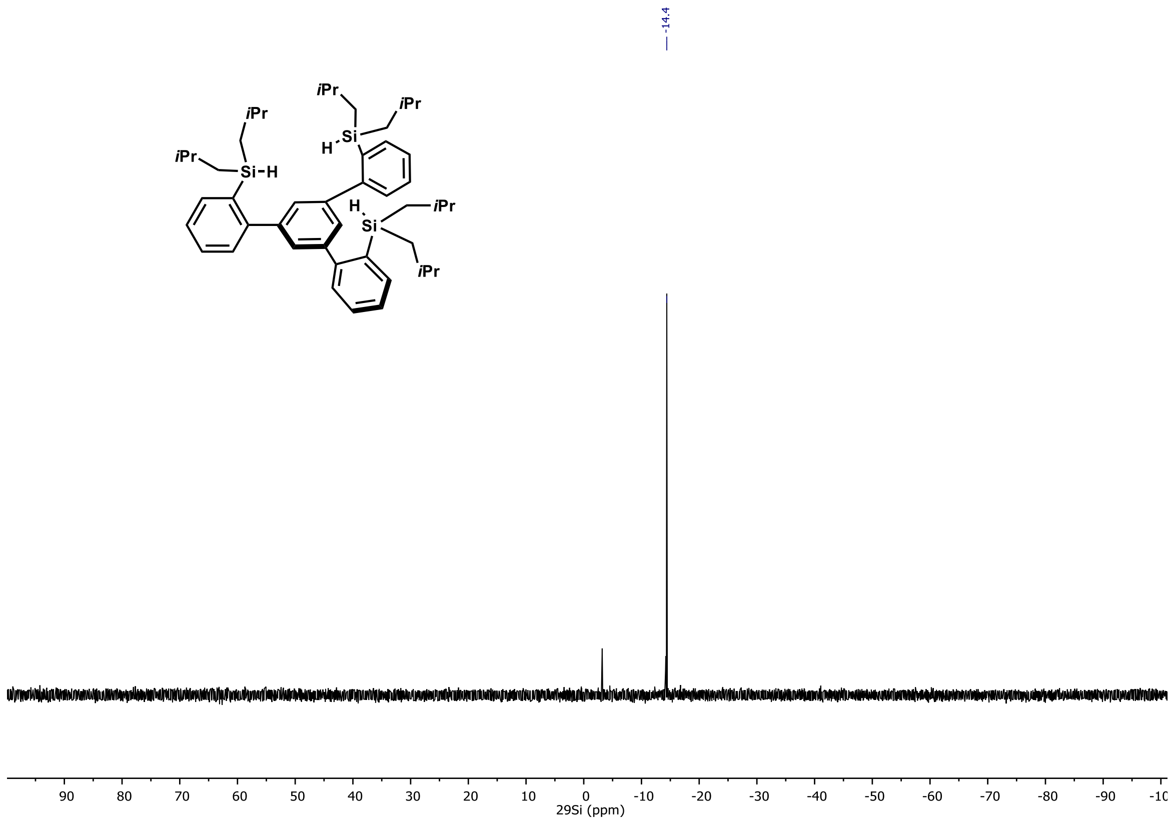

^{1}H NMR of Ligand 3h, 600 MHz, $CDCl_3$, 25ºC

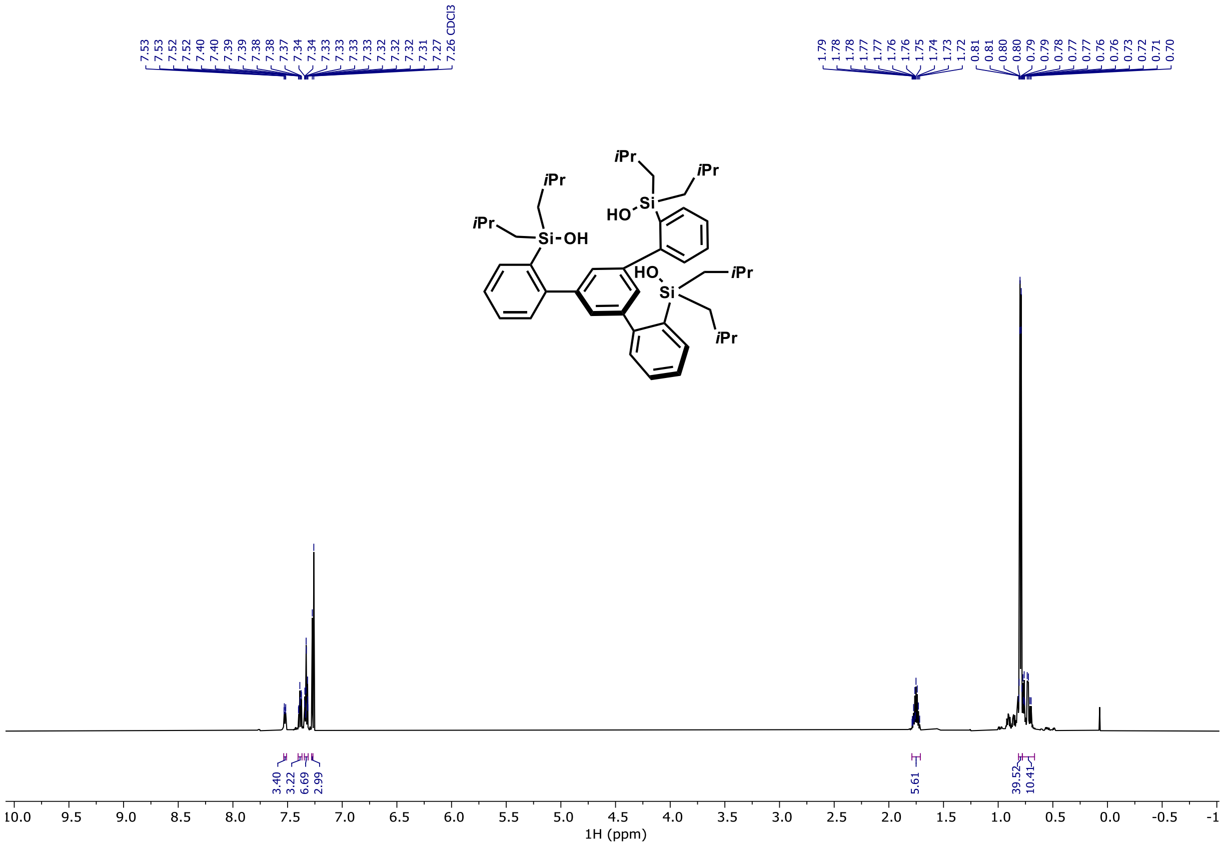

^{13}C NMR of Ligand 3h, 151 MHz, $CDCl_3$, 25ºC

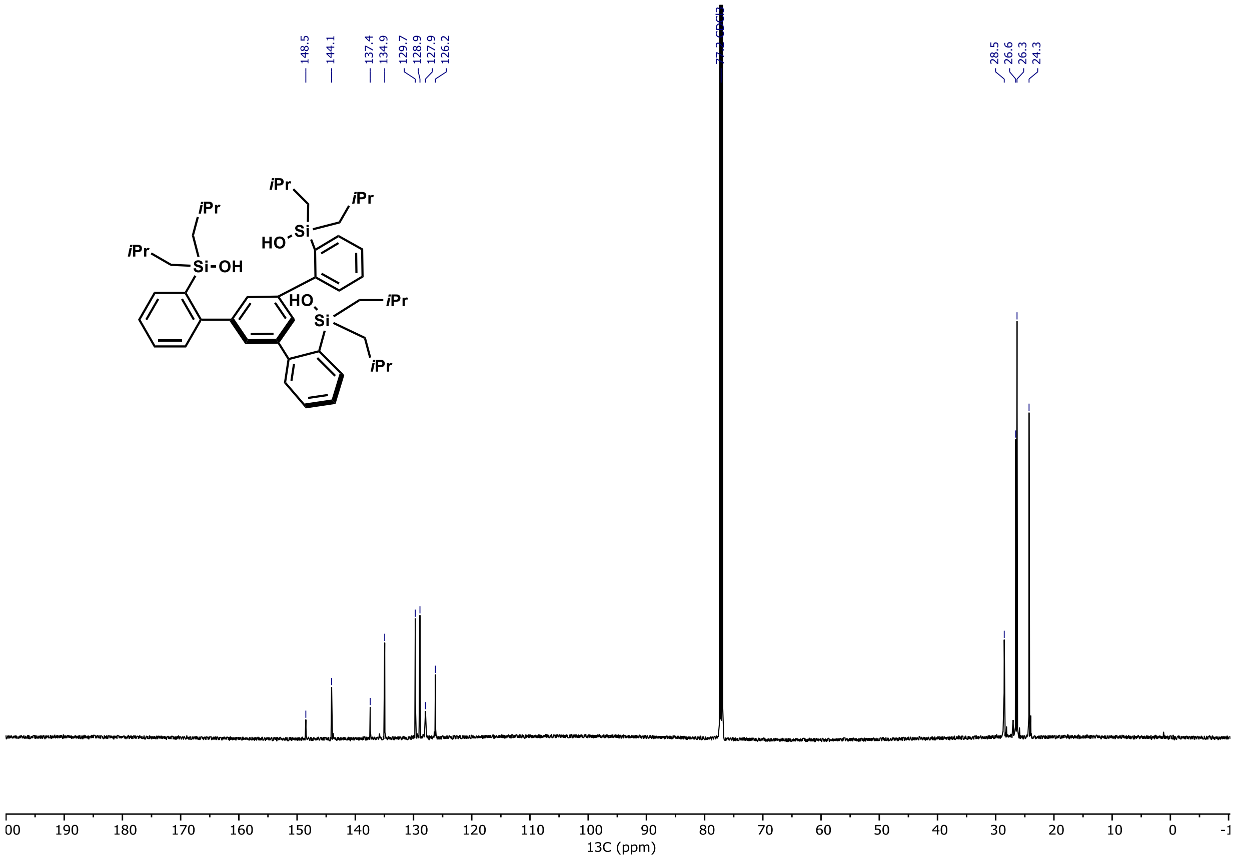

^{29}Si NMR of Ligand 3h, 119 MHz, $CDCl_3$, 25ºC

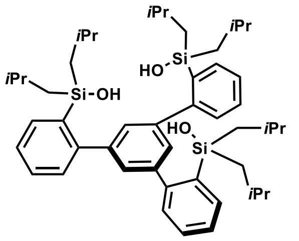

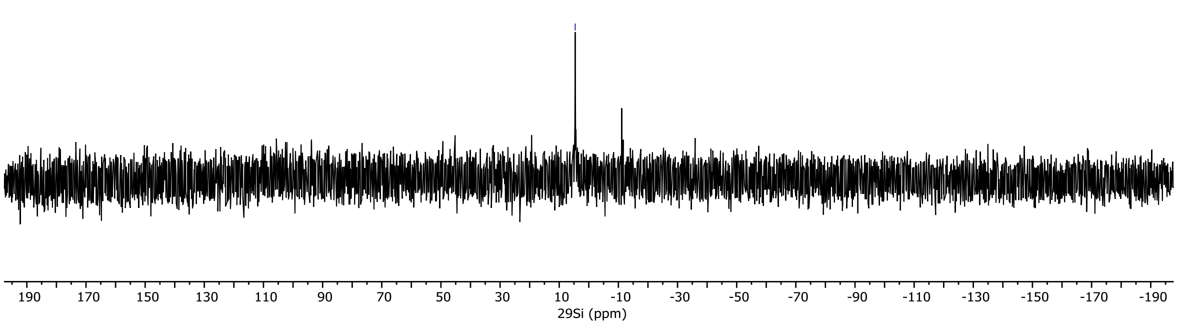
4.6
190
170
150
130
110
90
70
50
30
10
-10
-30
-50
-70
-90
-110
-130
-150
-170
-190
29Si (ppm)

^{1}H NMR of Silane S4, 500 MHz, $CDCl_3$, 25°C

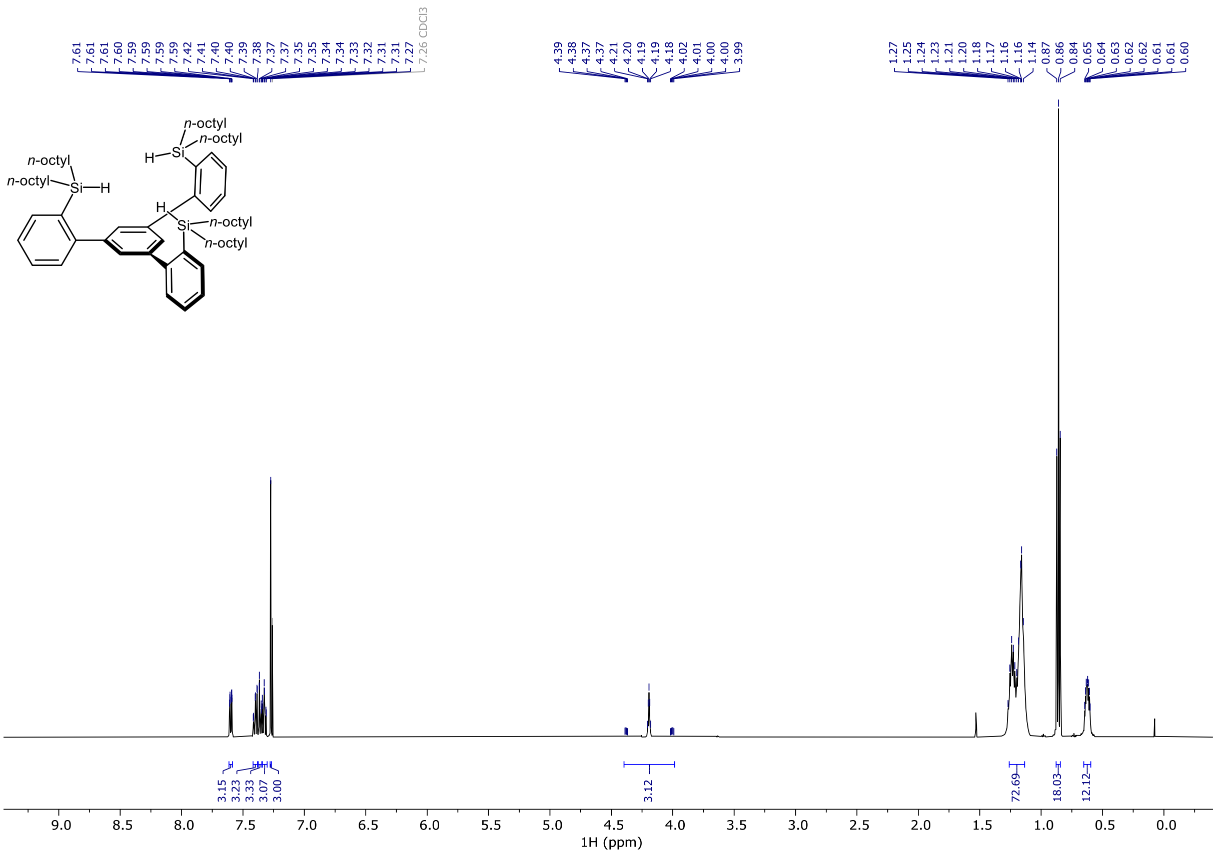

^{13}C NMR of Silane S4, 126 MHz, $CDCl_3$, 25°C

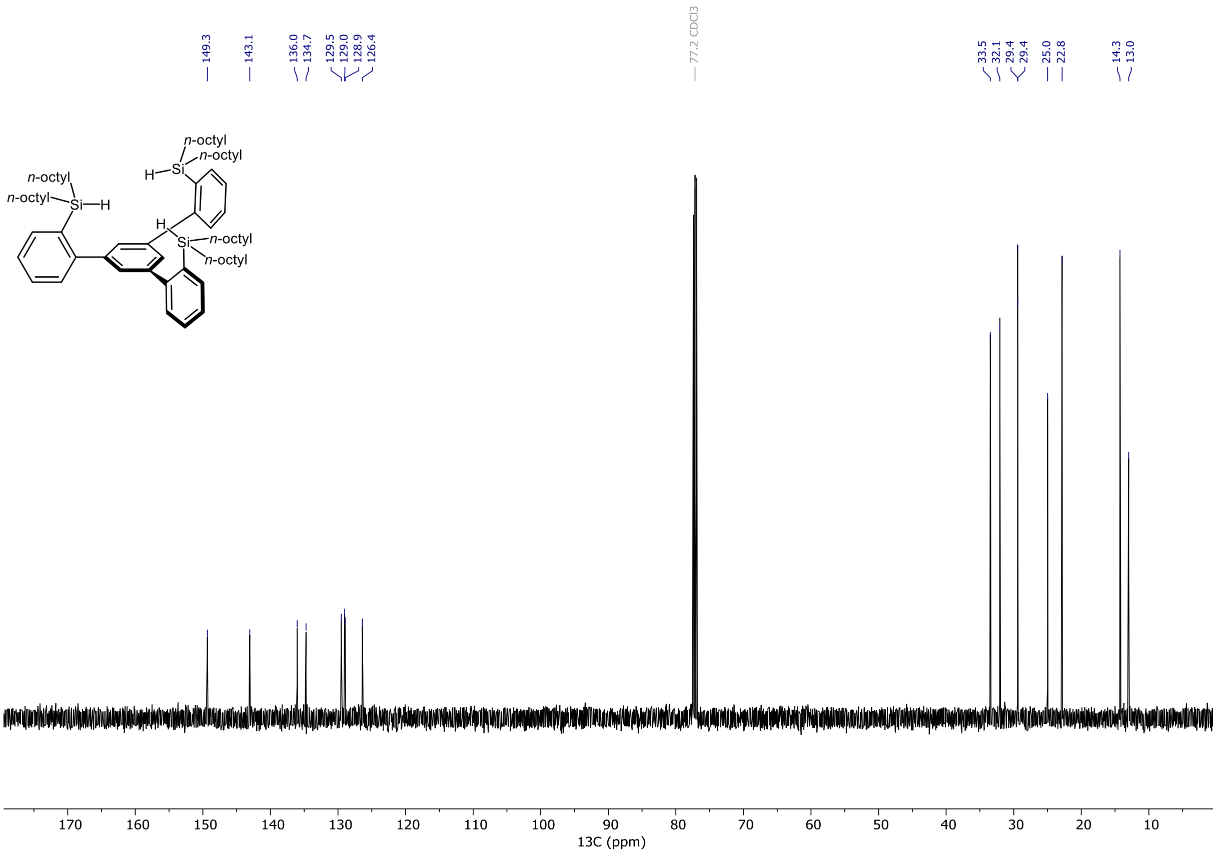

^{29}Si NMR of Silane S4, 99 MHz, $CDCl_3$, 25°C

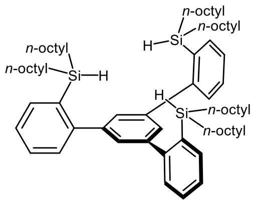

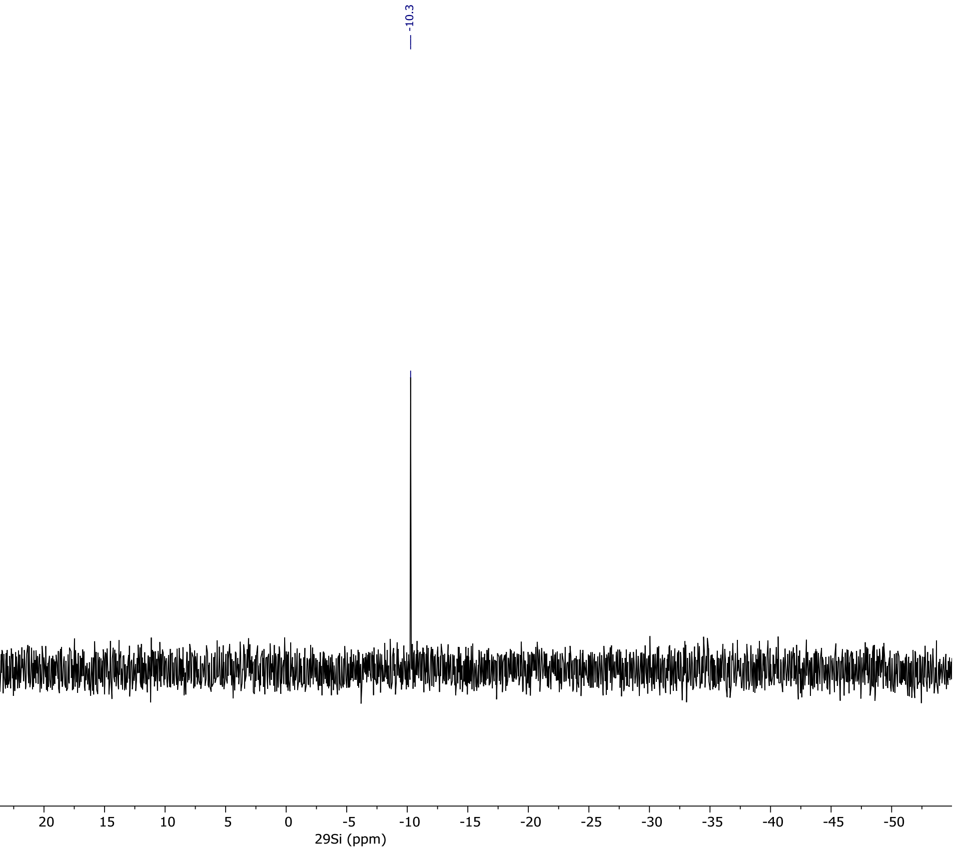

^{1}H NMR of Ligand 3i, 600 MHz, $CDCl_3$, 25°C

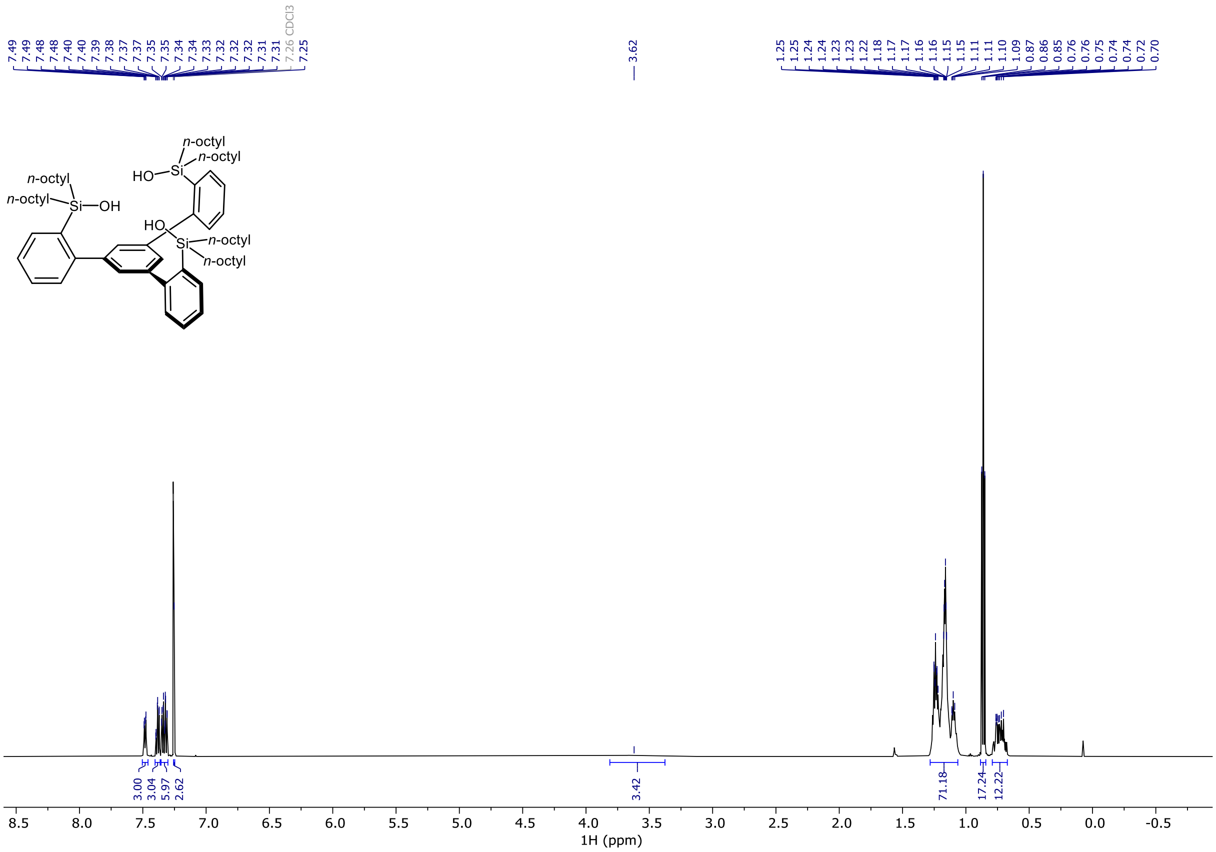

^{13}C NMR of Ligand 3i, 151 MHz, $CDCl_3$, 25°C

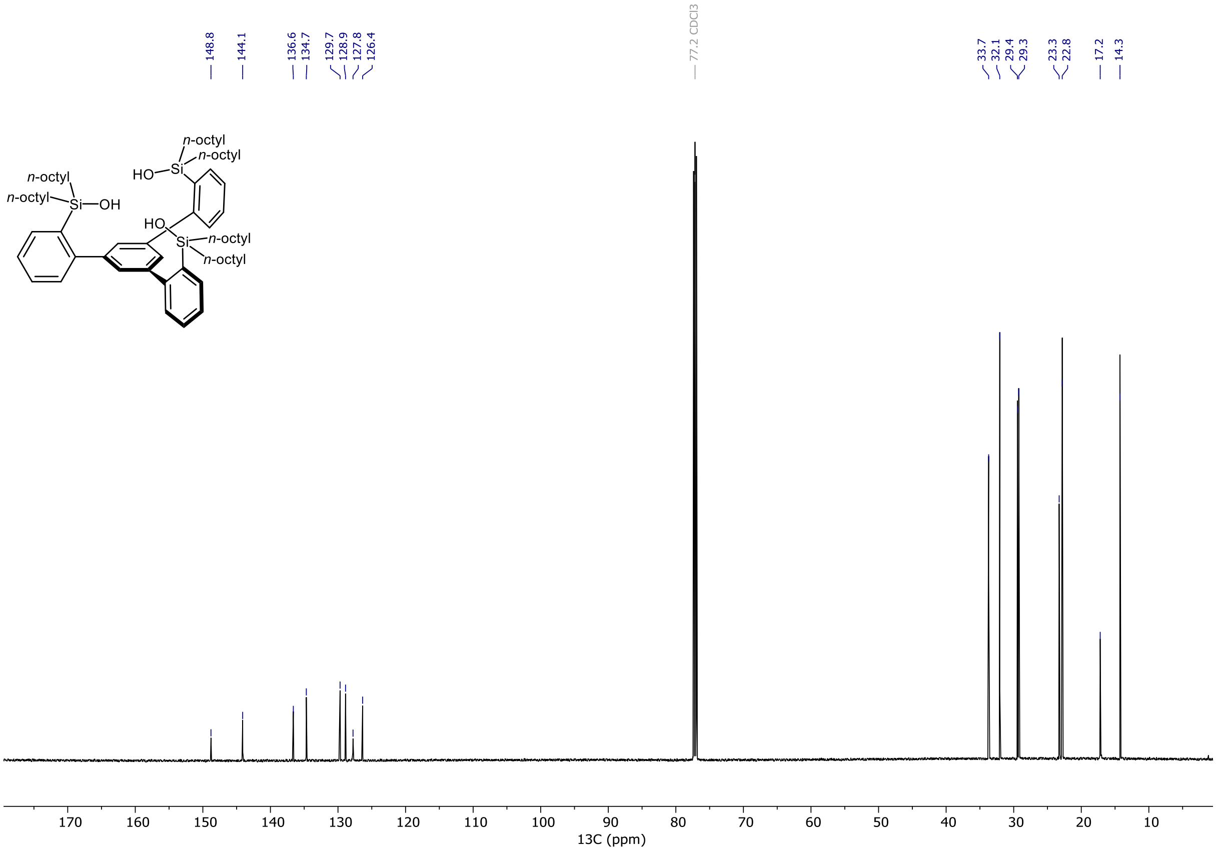

^{29}Si NMR of Ligand 3i, 119 MHz, $CDCl_3$, 25°C

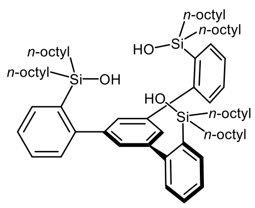

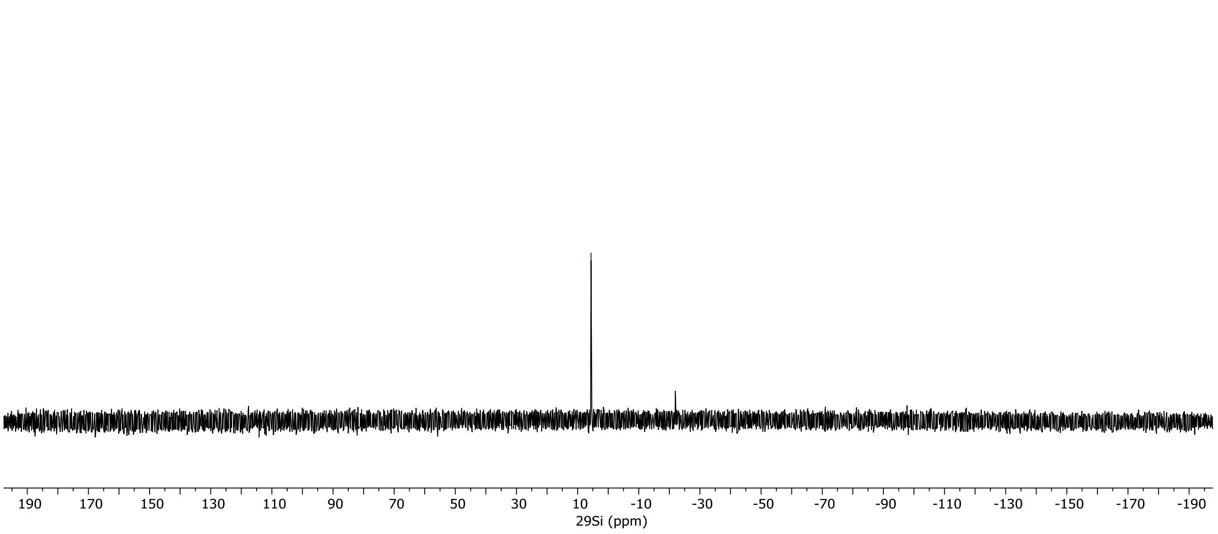

^{1}H NMR of Complex 1f, [D$_8$]-toluene, 25°C

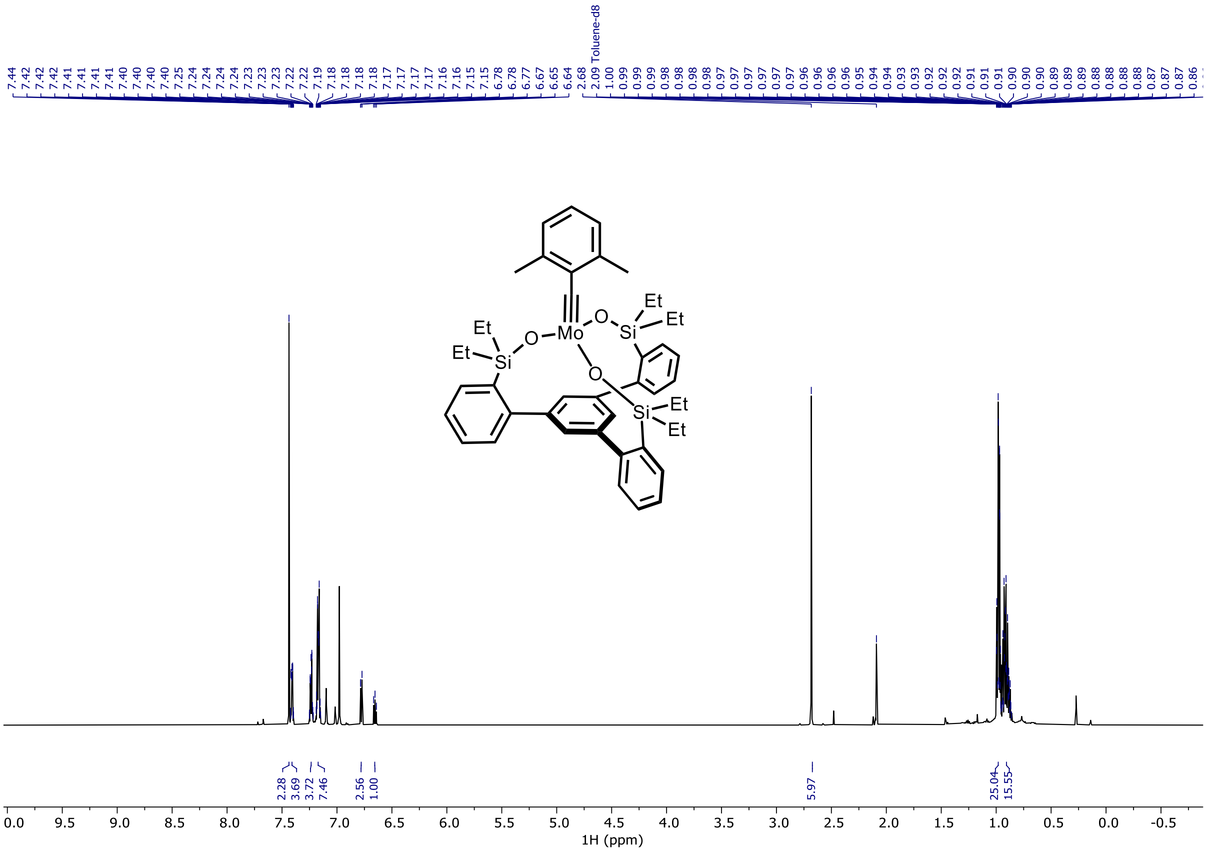

^{13}C NMR of Complex 1f, [D$_8$]-toluene, 25°C

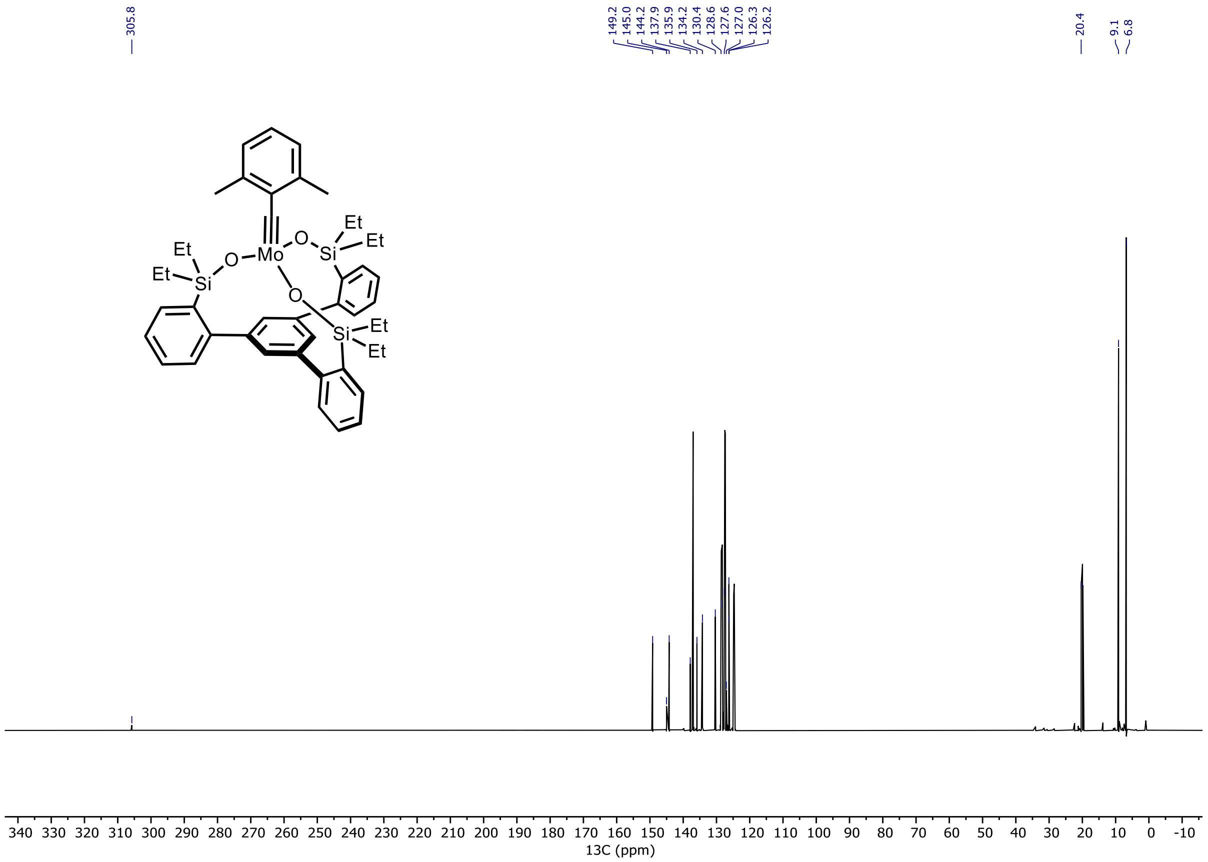

^{29}Si NMR of Complex 1f, [D$_8$]-toluene, 25°C

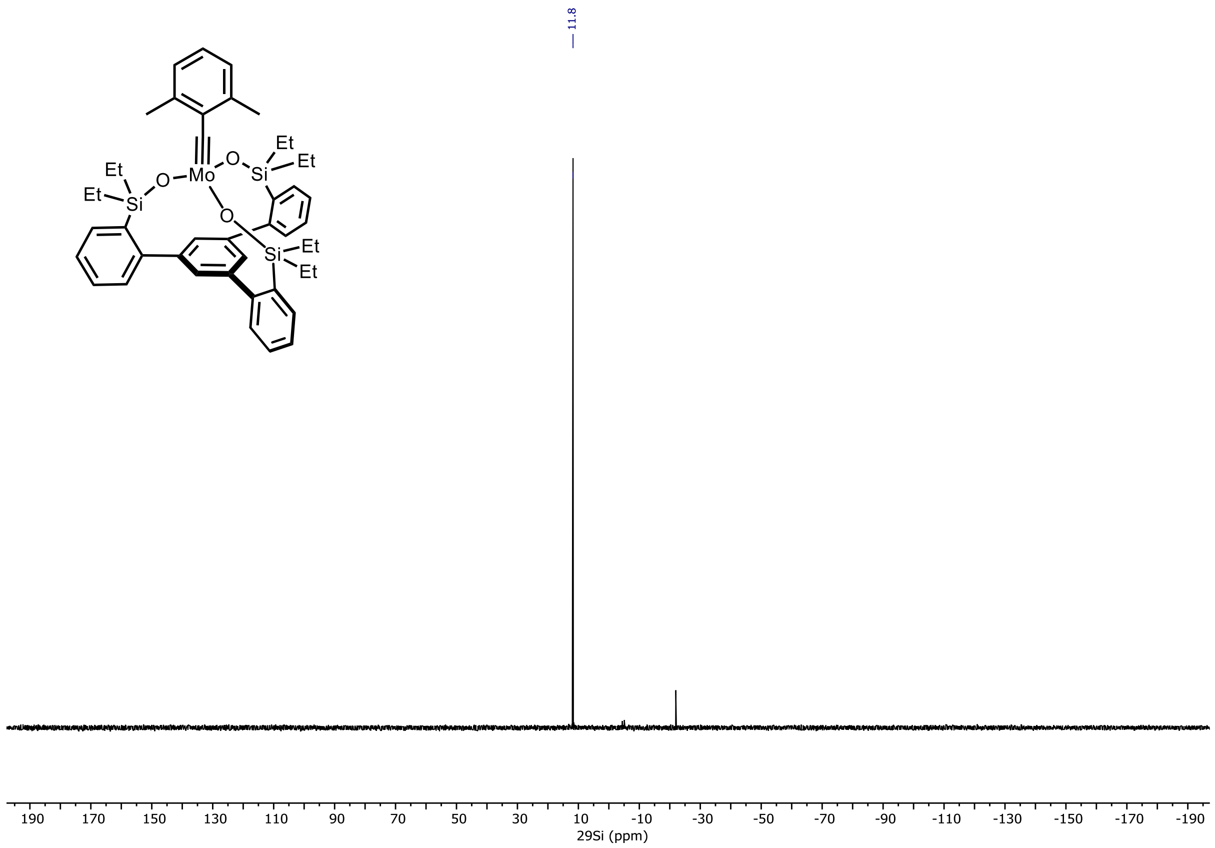

^{95}Mo NMR of Complex 1f, [D$_8$]-toluene, 60°C

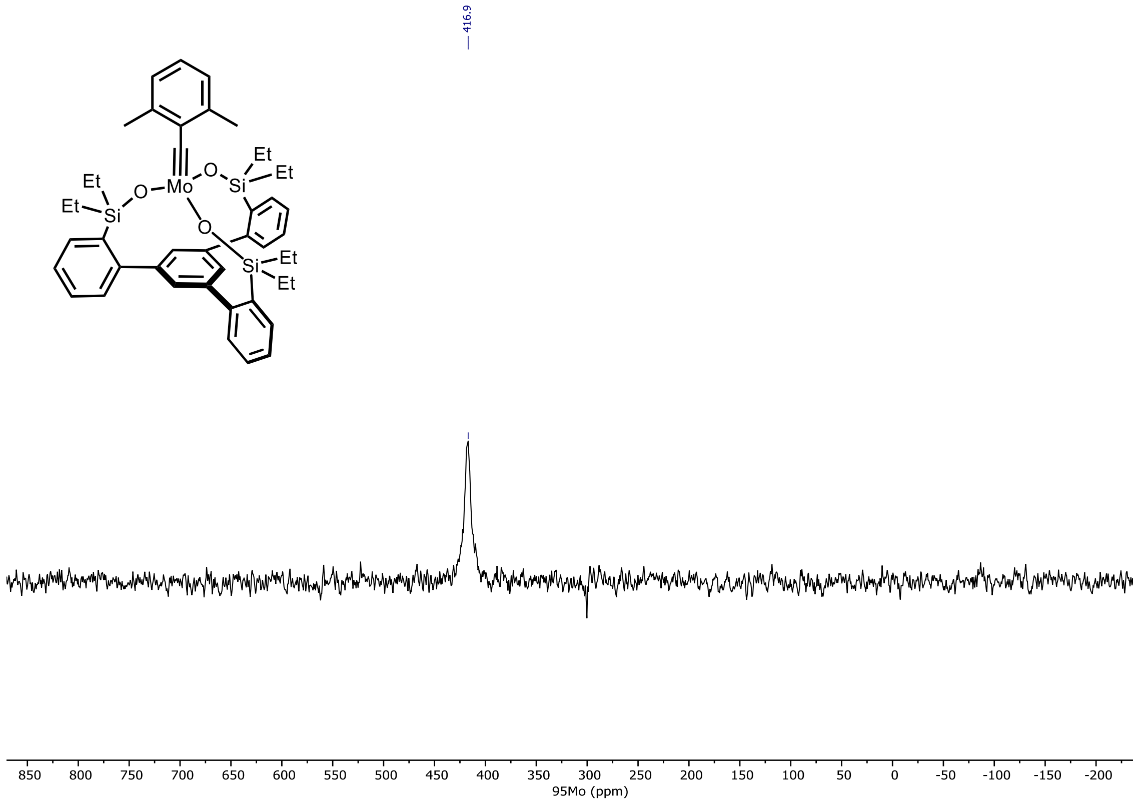

^{1}H NMR of Complex 1g, 600 MHz, [D$_8$]-toluene, 25°C

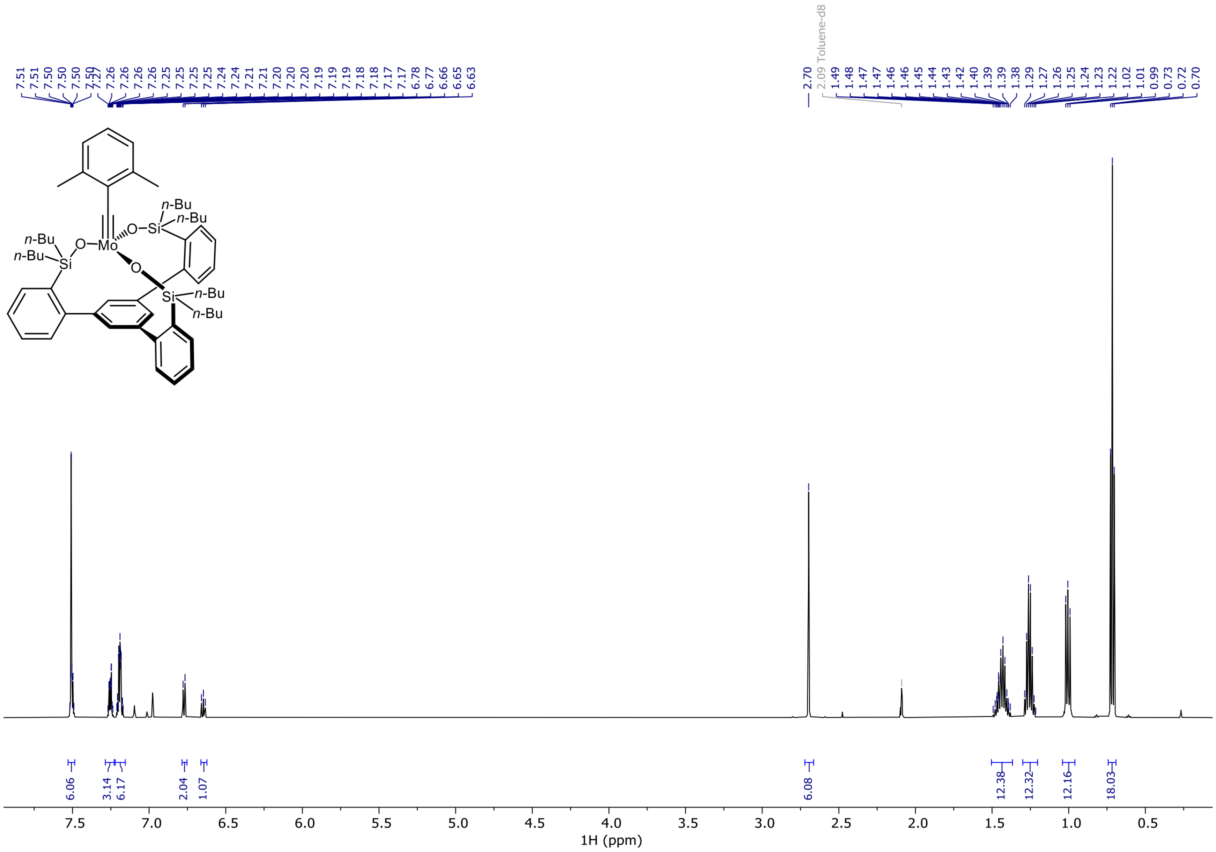

^{13}C NMR of Complex 1g, 151 MHz, [D$_8$]-toluene, 25°C

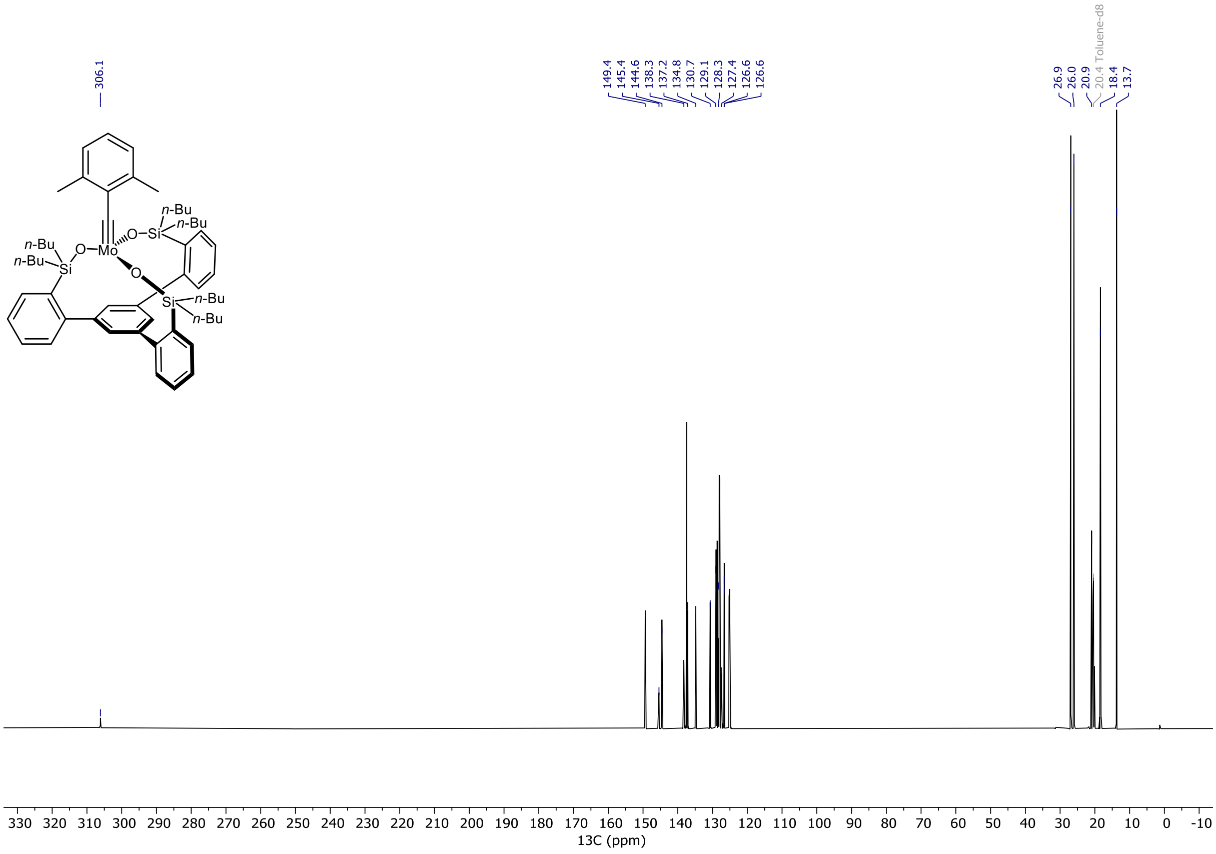

^{29}Si NMR of Complex 1g, 119 MHz, [D8]-toluene, 25°C

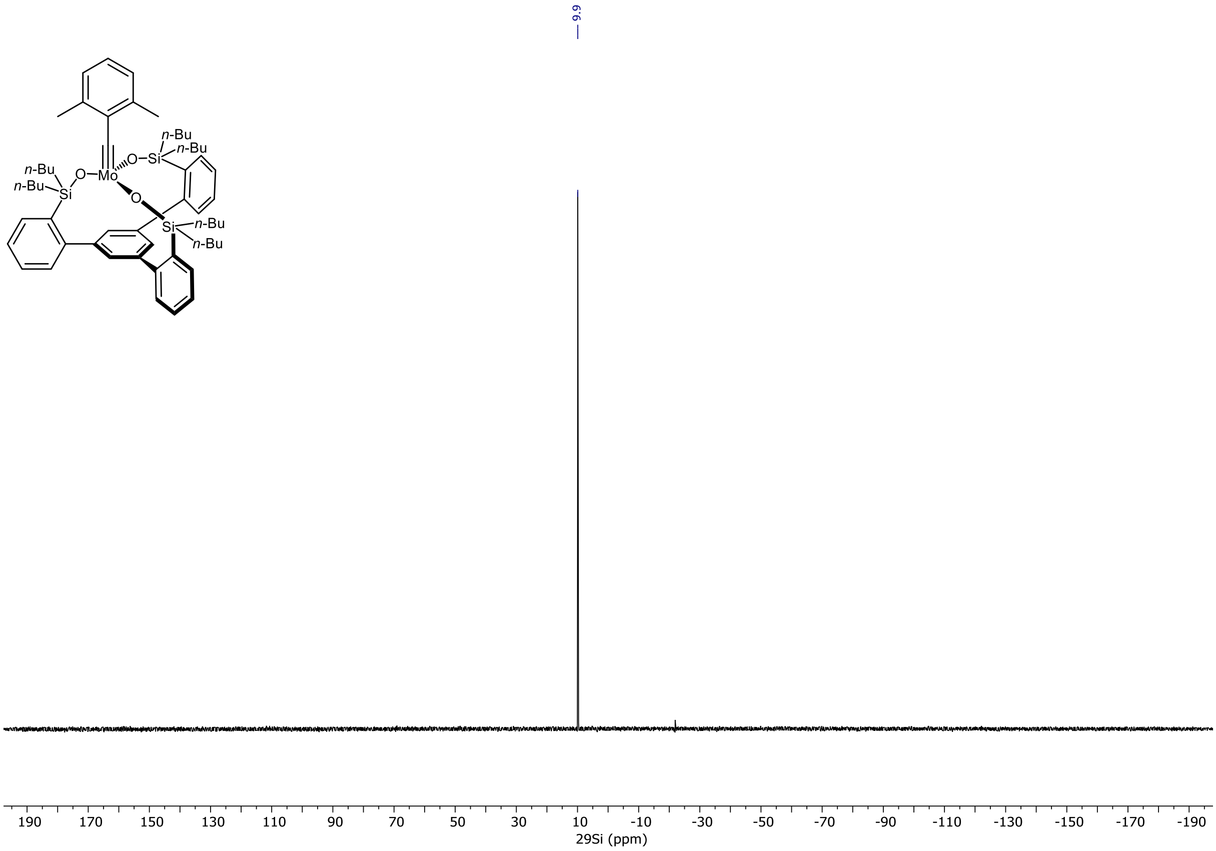

^{95}Mo NMR of Complex 1g, 26 MHz, [D8]-toluene, 60°C

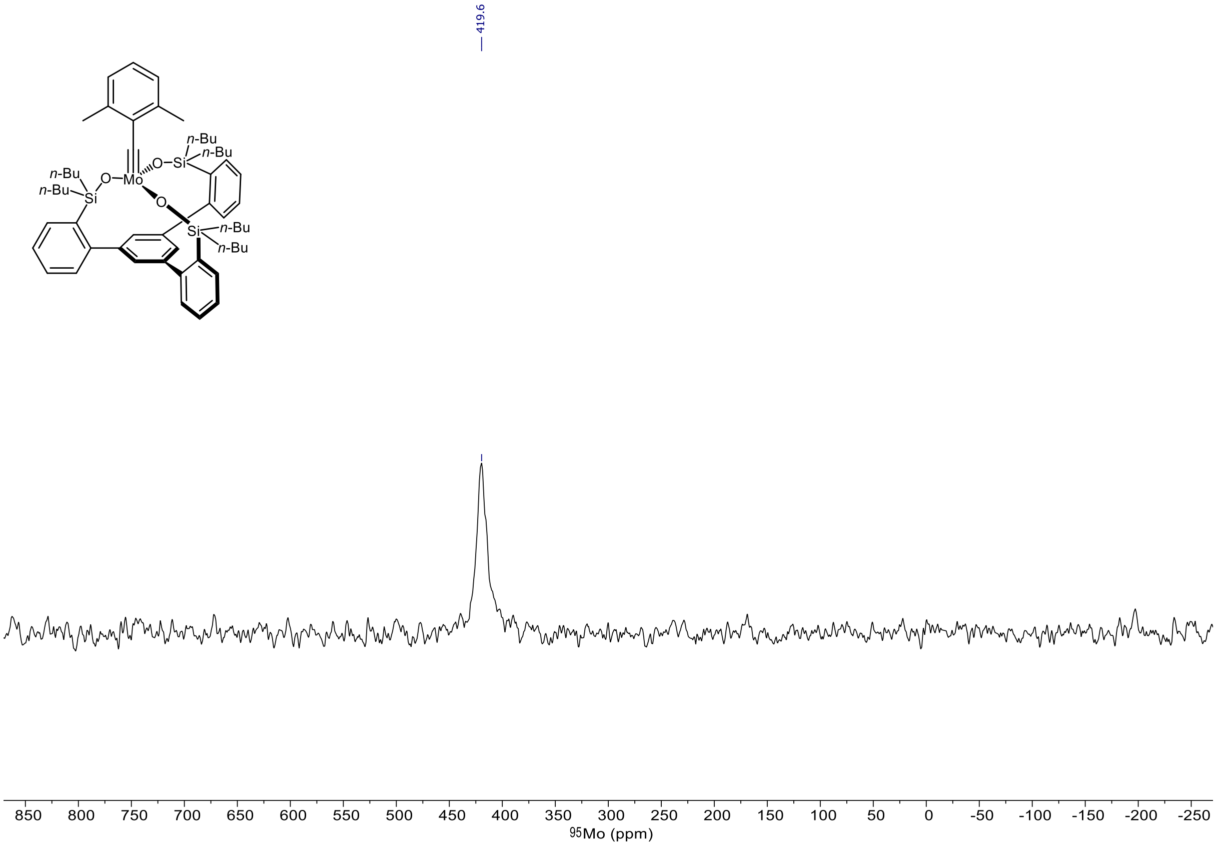

^{1}H NMR of Complex 1h, 600 MHz, [D$_8$]-toluene, 25°C

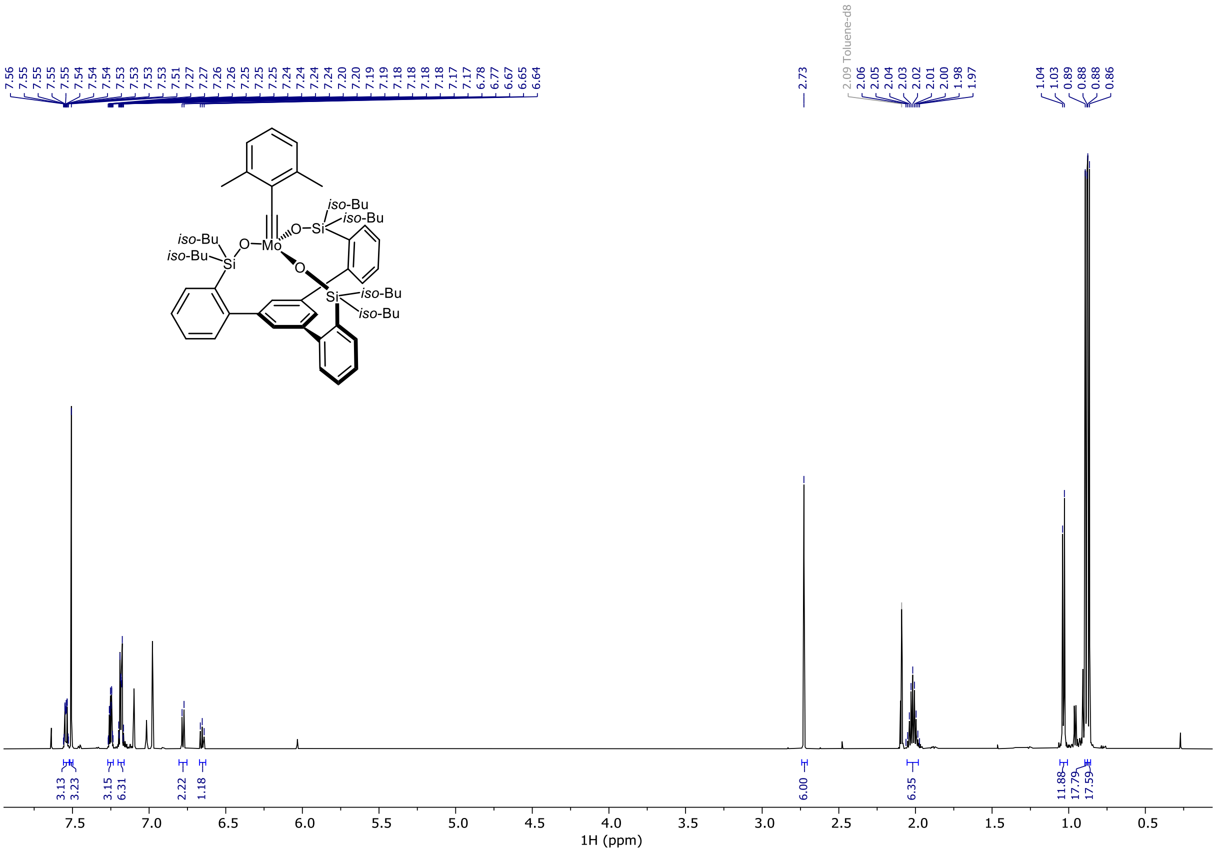

^{13}C NMR of Complex 1h, 151 MHz, [D$_8$]-toluene, 25°C

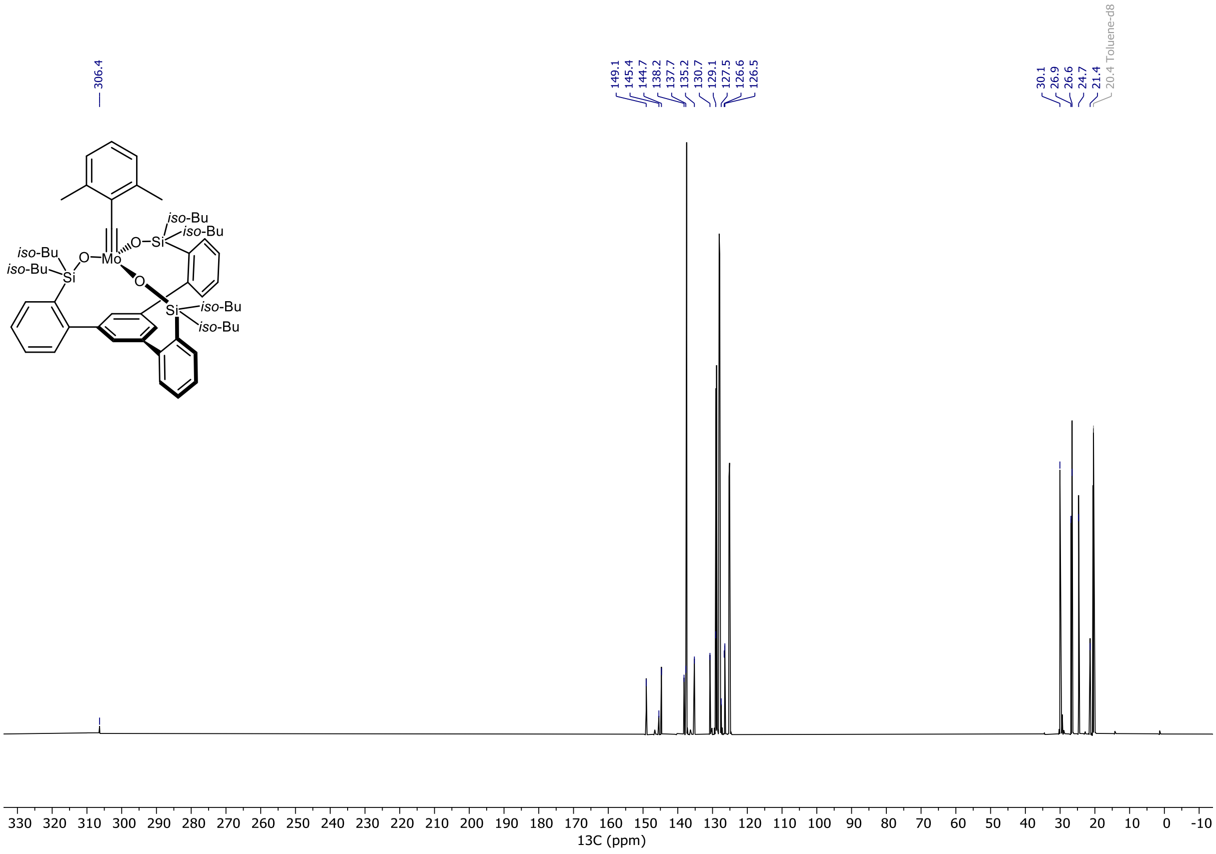

^{29}Si NMR of Complex 1h, 119 MHz, [D_8]-toluene, 25°C

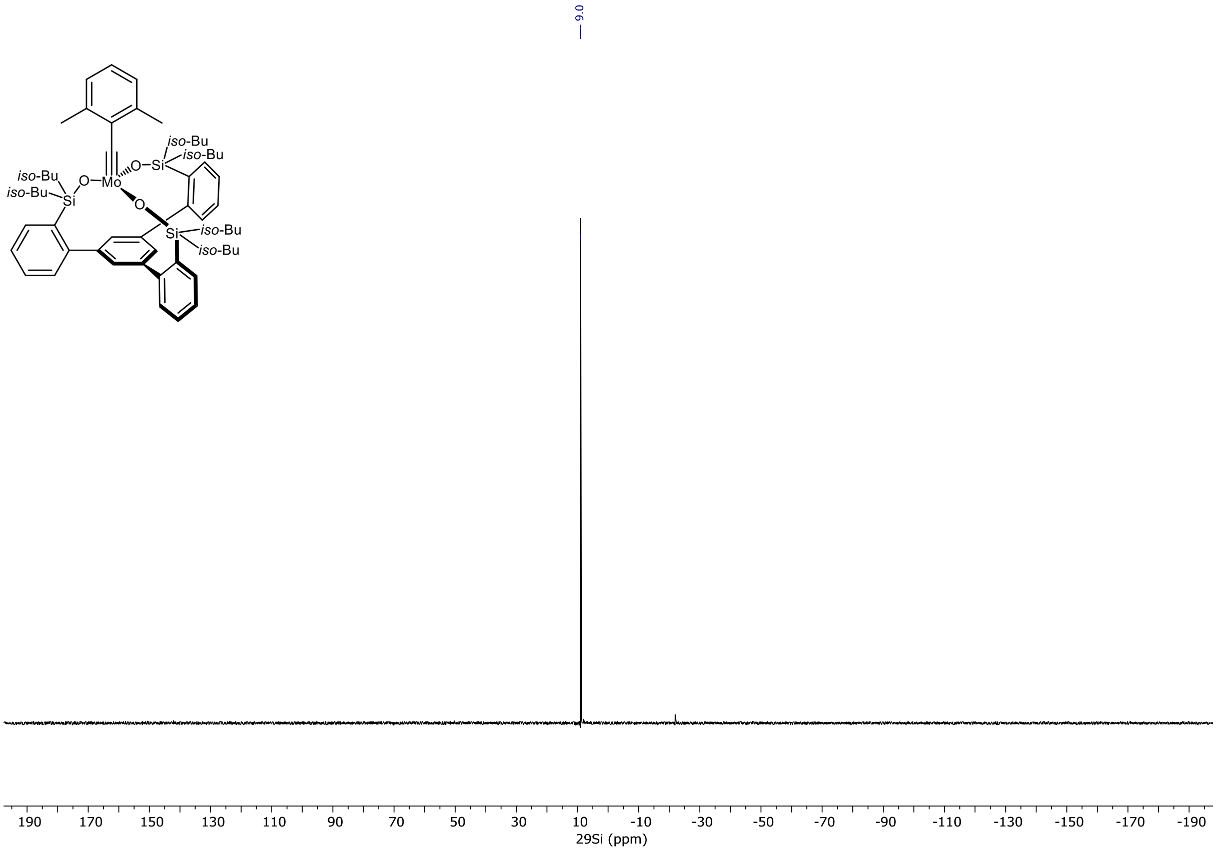

^{95}Mo NMR of Complex 1h, 26 MHz, [D_8]-toluene, 60°C

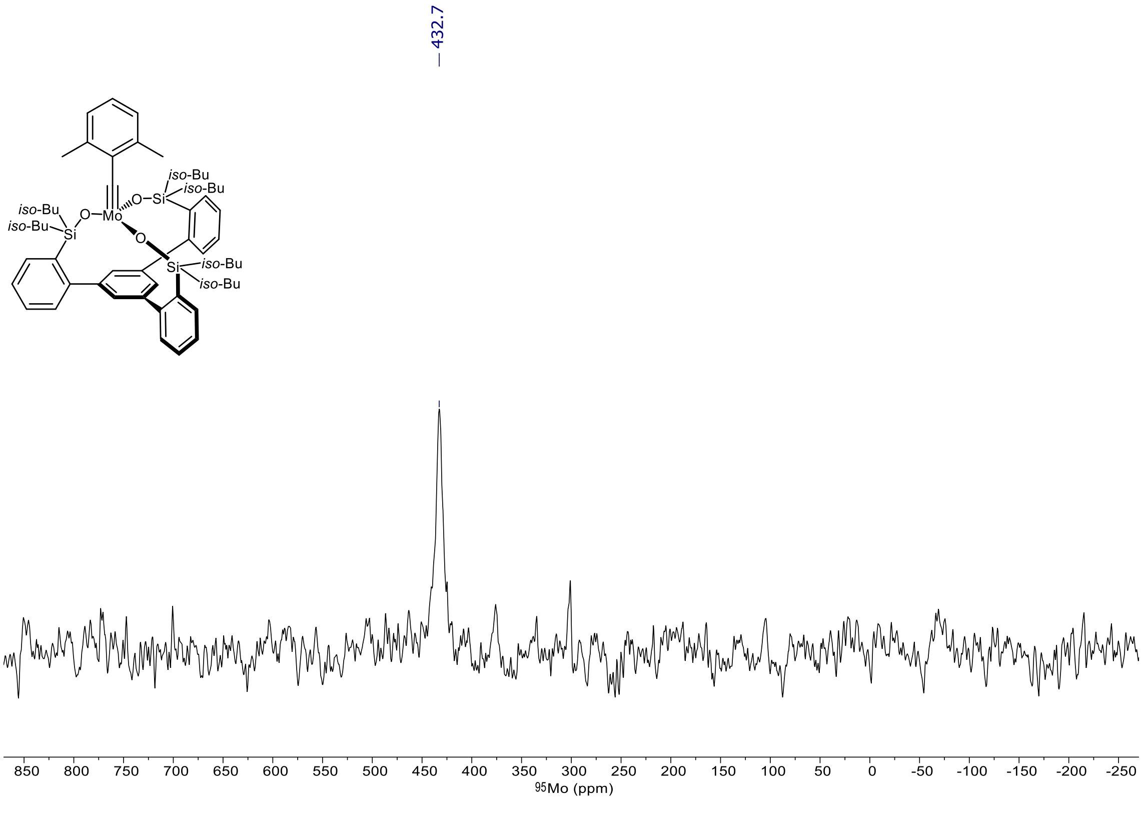

^{1}H NMR of Complex 1i, 600 MHz, [D$_8$]-toluene, 25°C

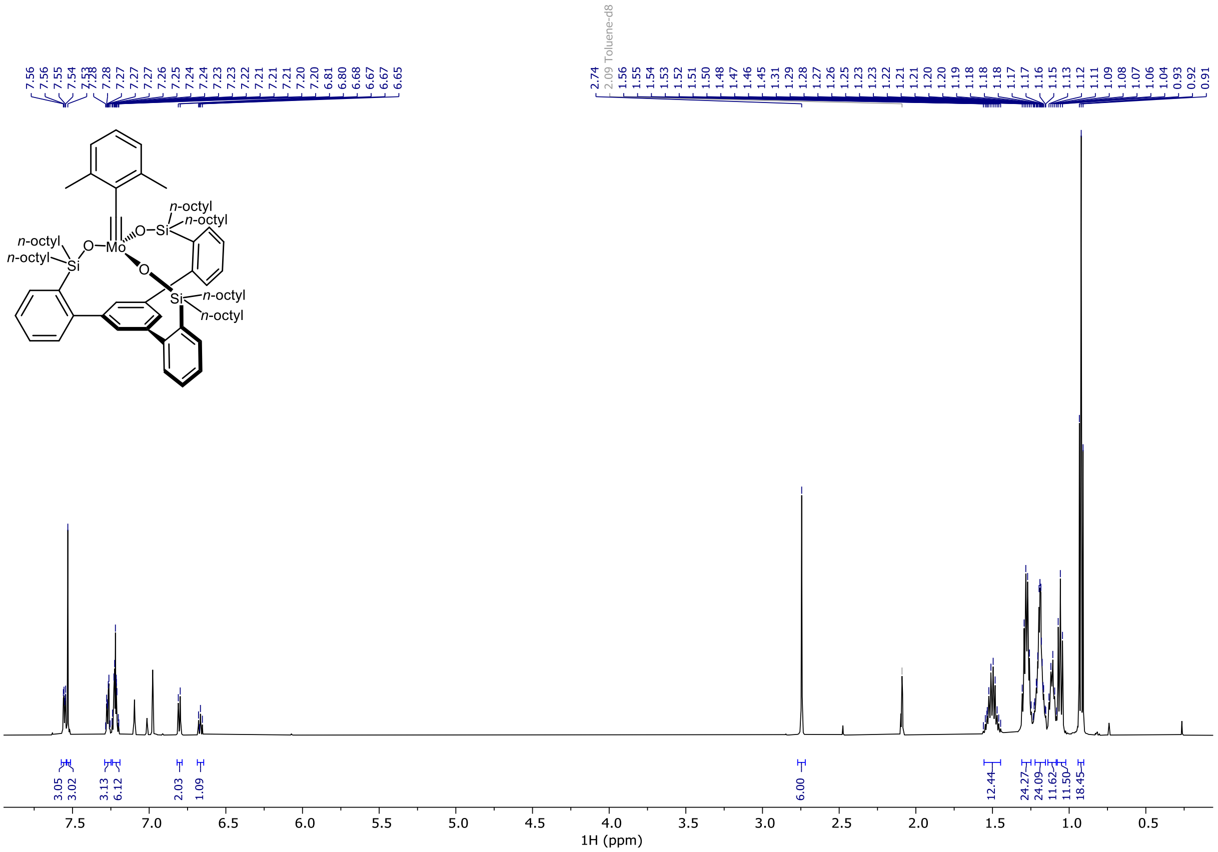

^{13}C NMR of Complex 1i, 151 MHz, [D$_8$]-toluene, 25°C

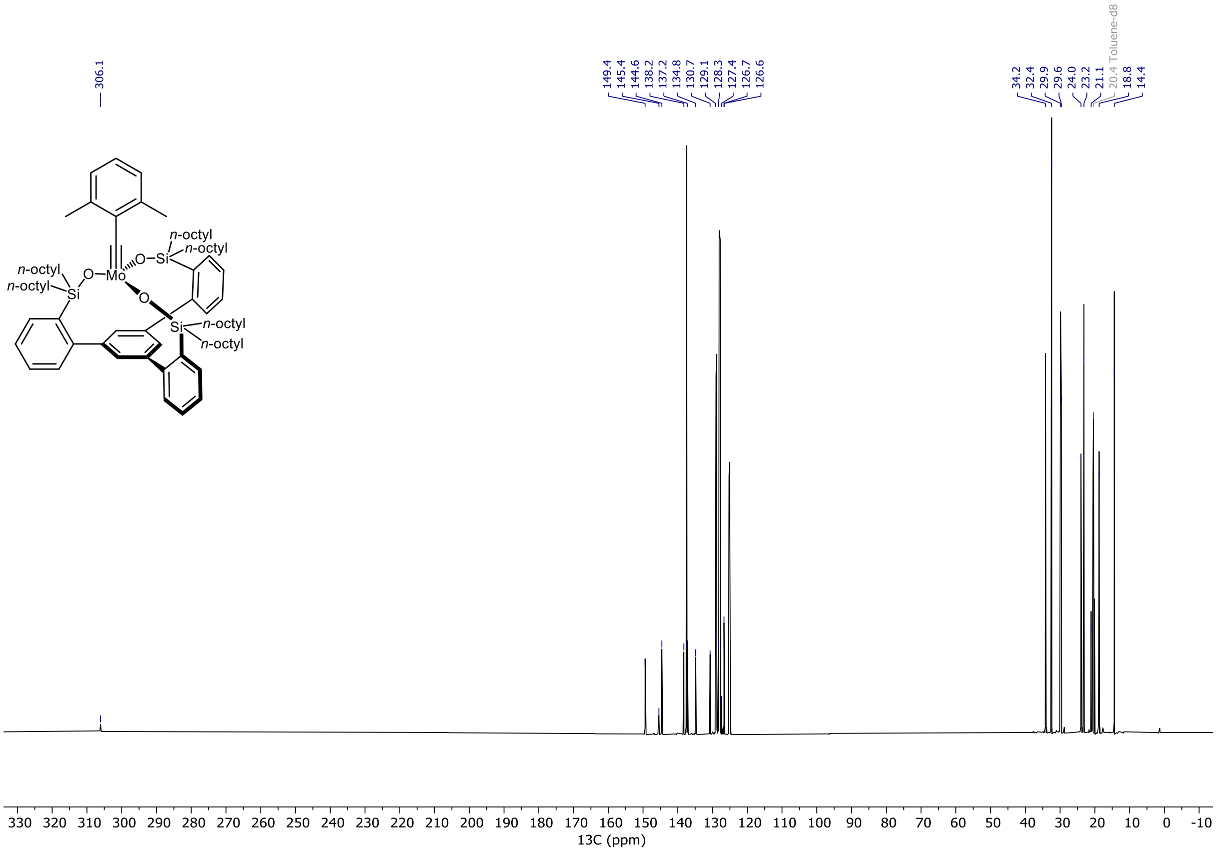

^{29}Si NMR of Complex 1i, 119 MHz, [D8]-toluene, 25°C

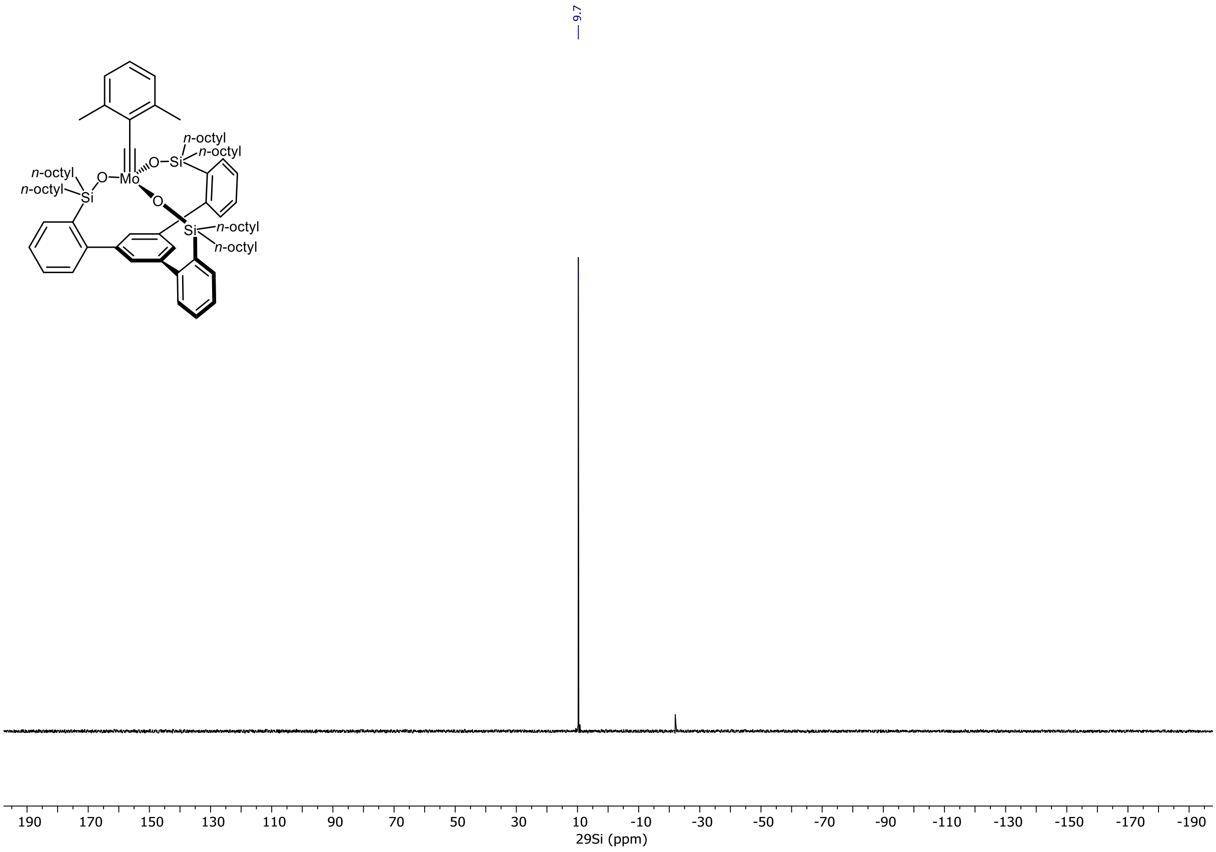

^{95}Mo NMR of Complex 1i, 26 MHz, [D8]-toluene, 60°C

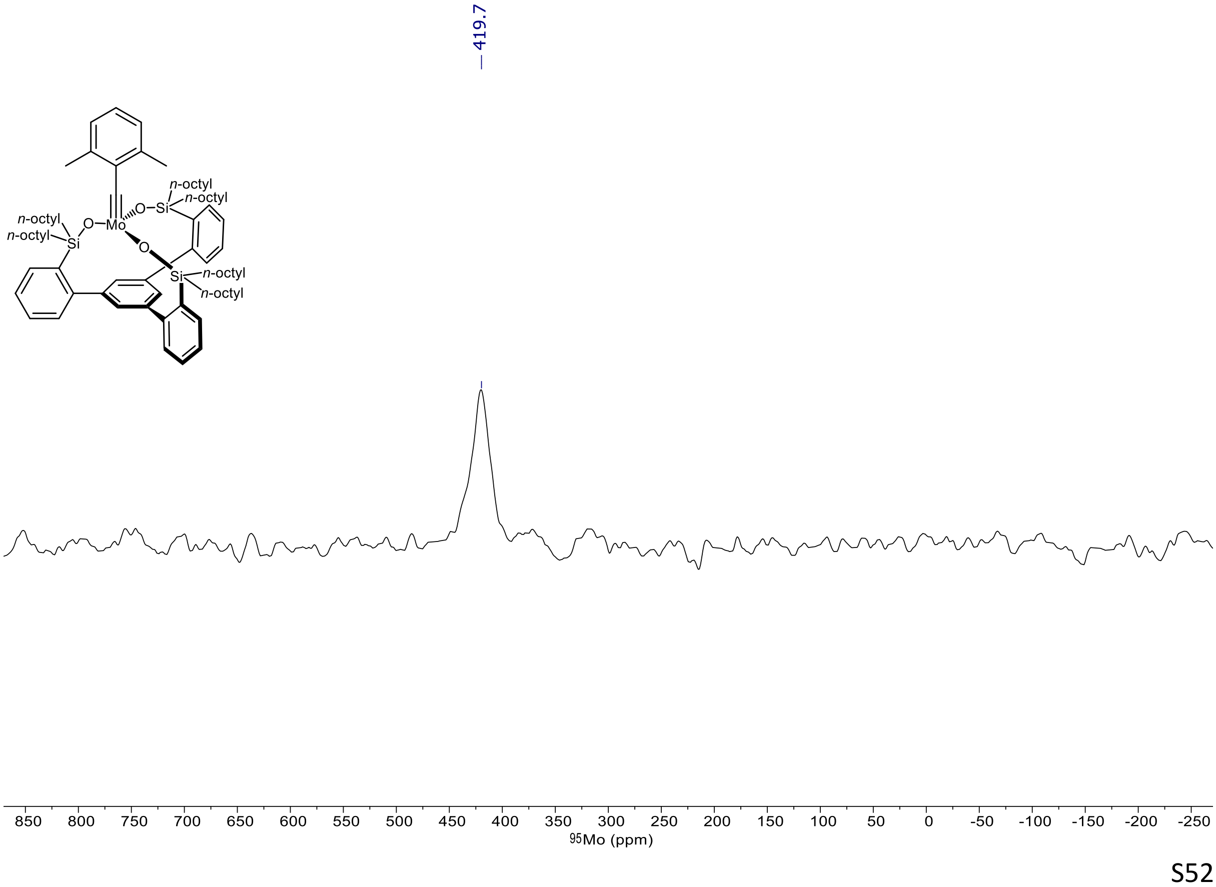

^{1}H NMR spectrum (600 MHz, [D$_8$]-toluene) of the dinuclear complex **8**

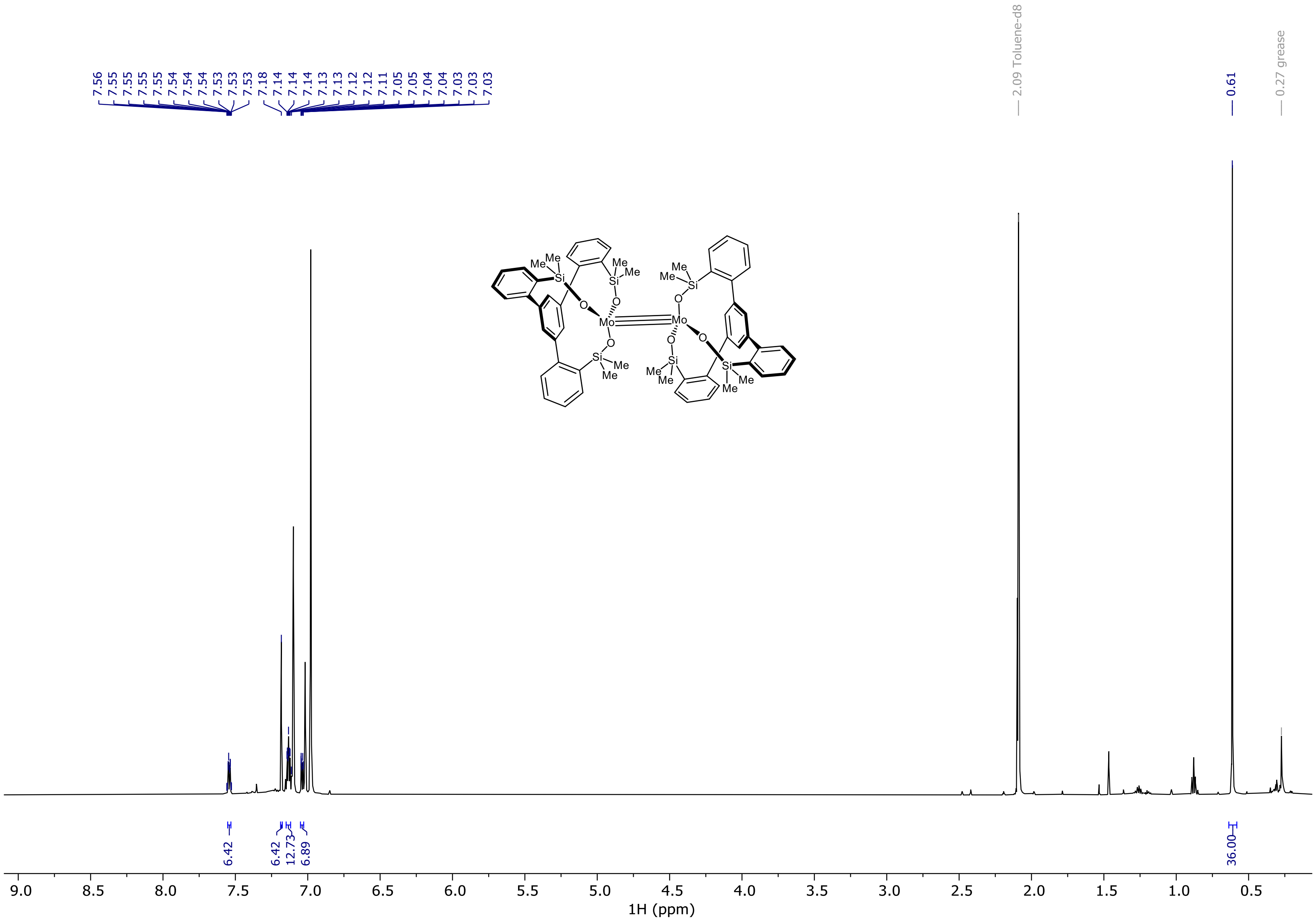

^{13}C NMR spectrum (151 MHz, $[D_8]$-toluene) of the dinuclear complex **8**

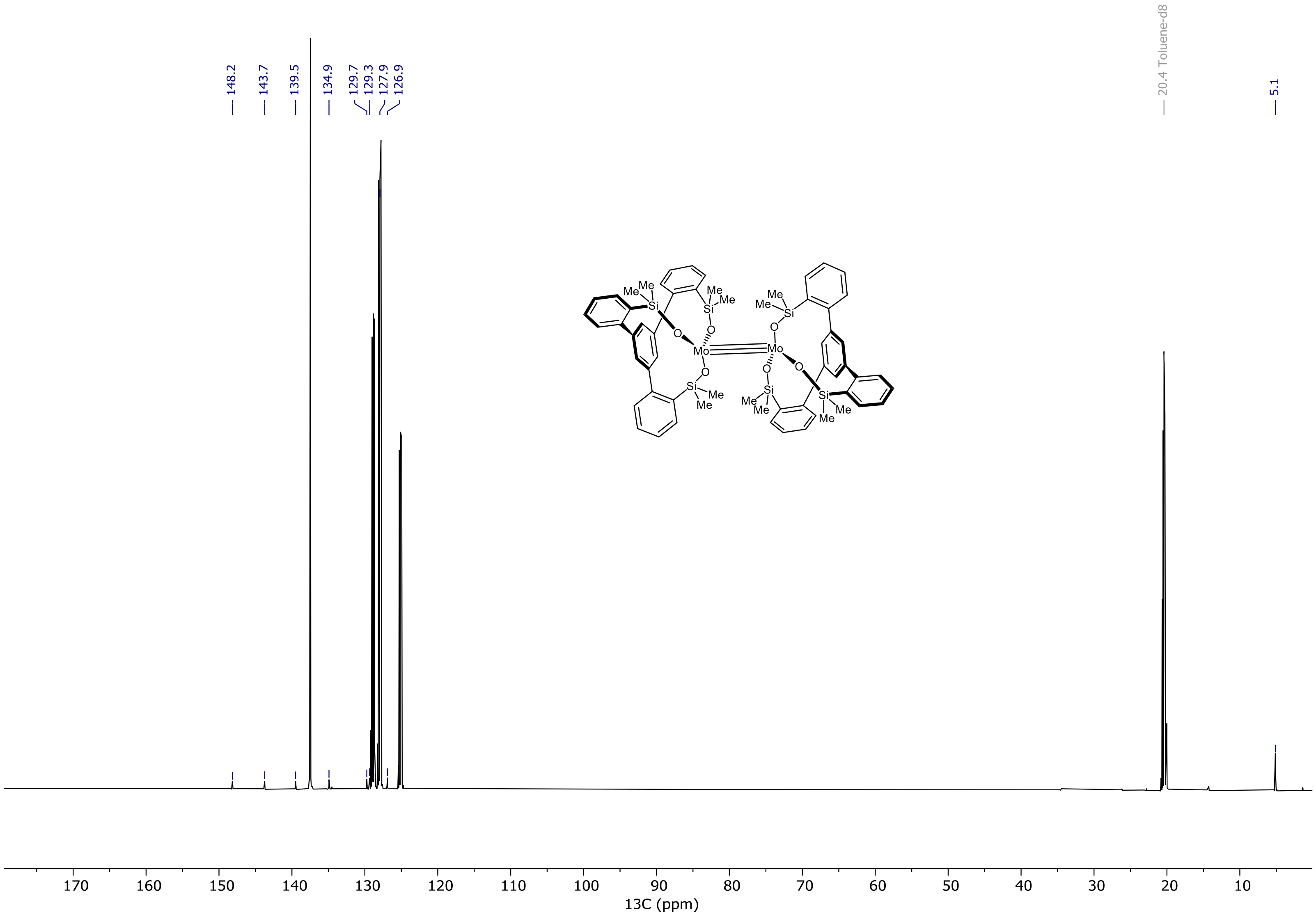

^{29}Si NMR spectrum (119 MHz, [D$_8$]-toluene) of the dinuclear complex **8**

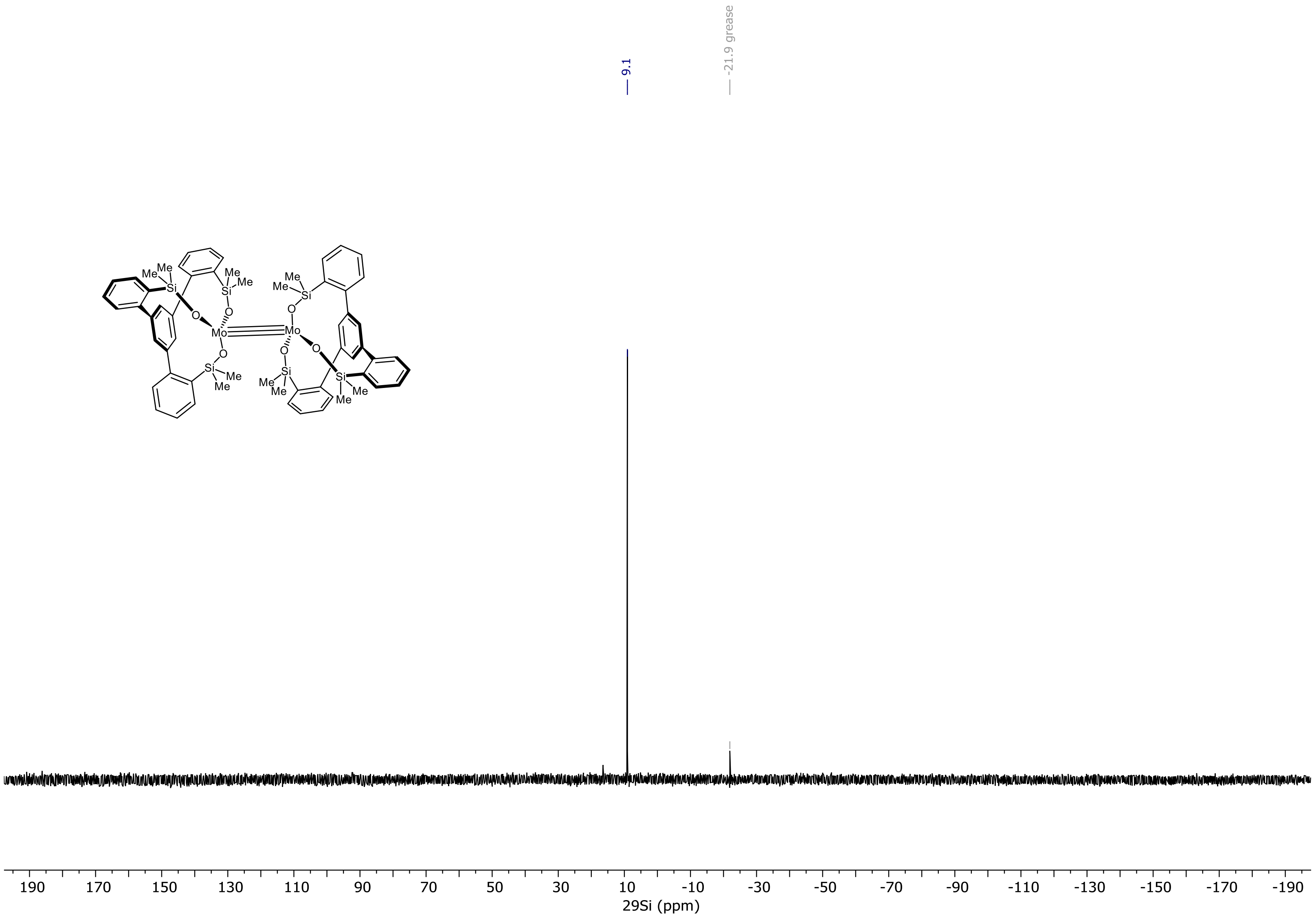

^{95}Mo NMR spectrum (25 MHz, [D_8]-toluene) of the dinuclear complex **8** at 25°C (top) and 60°C (bottom)

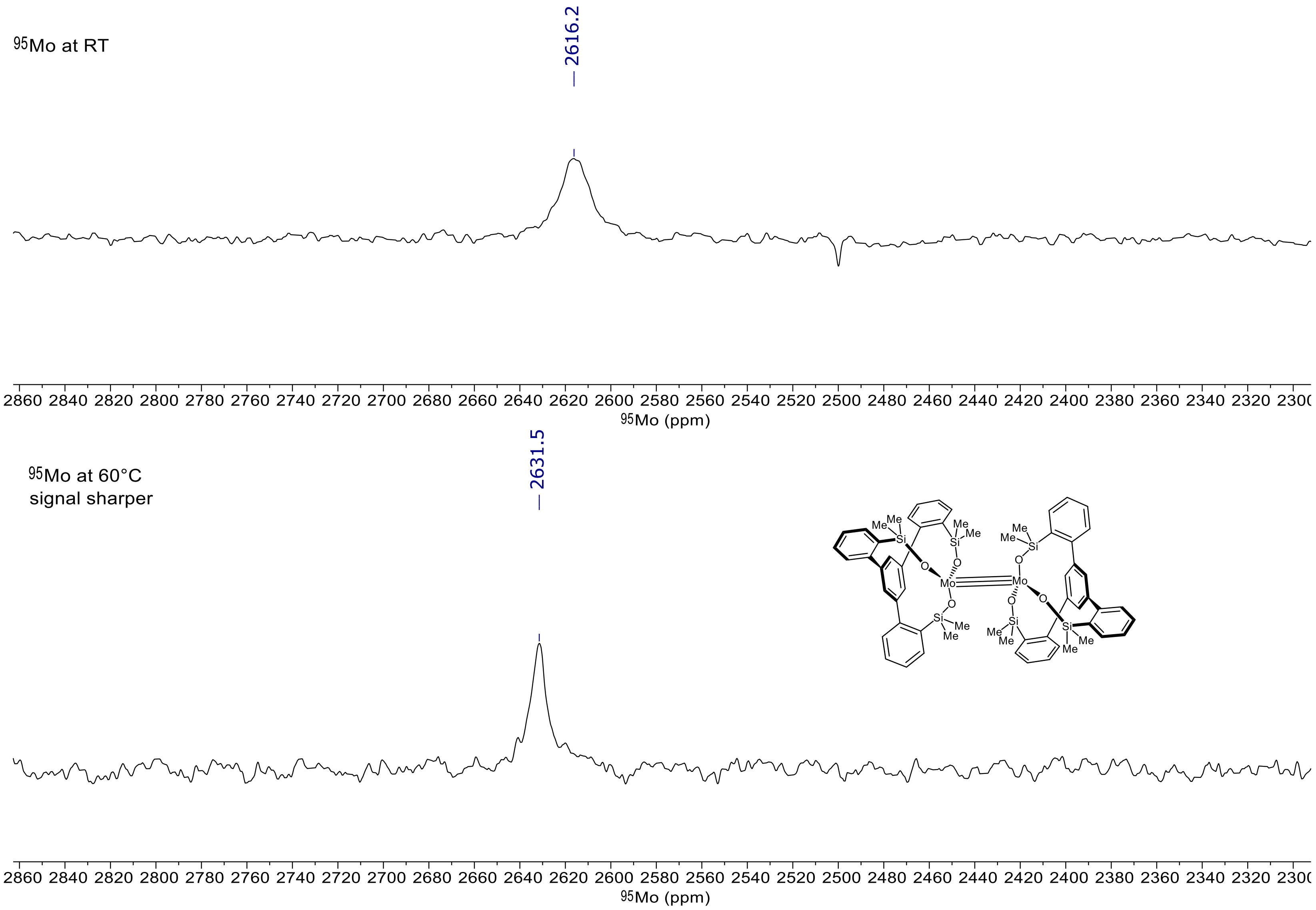

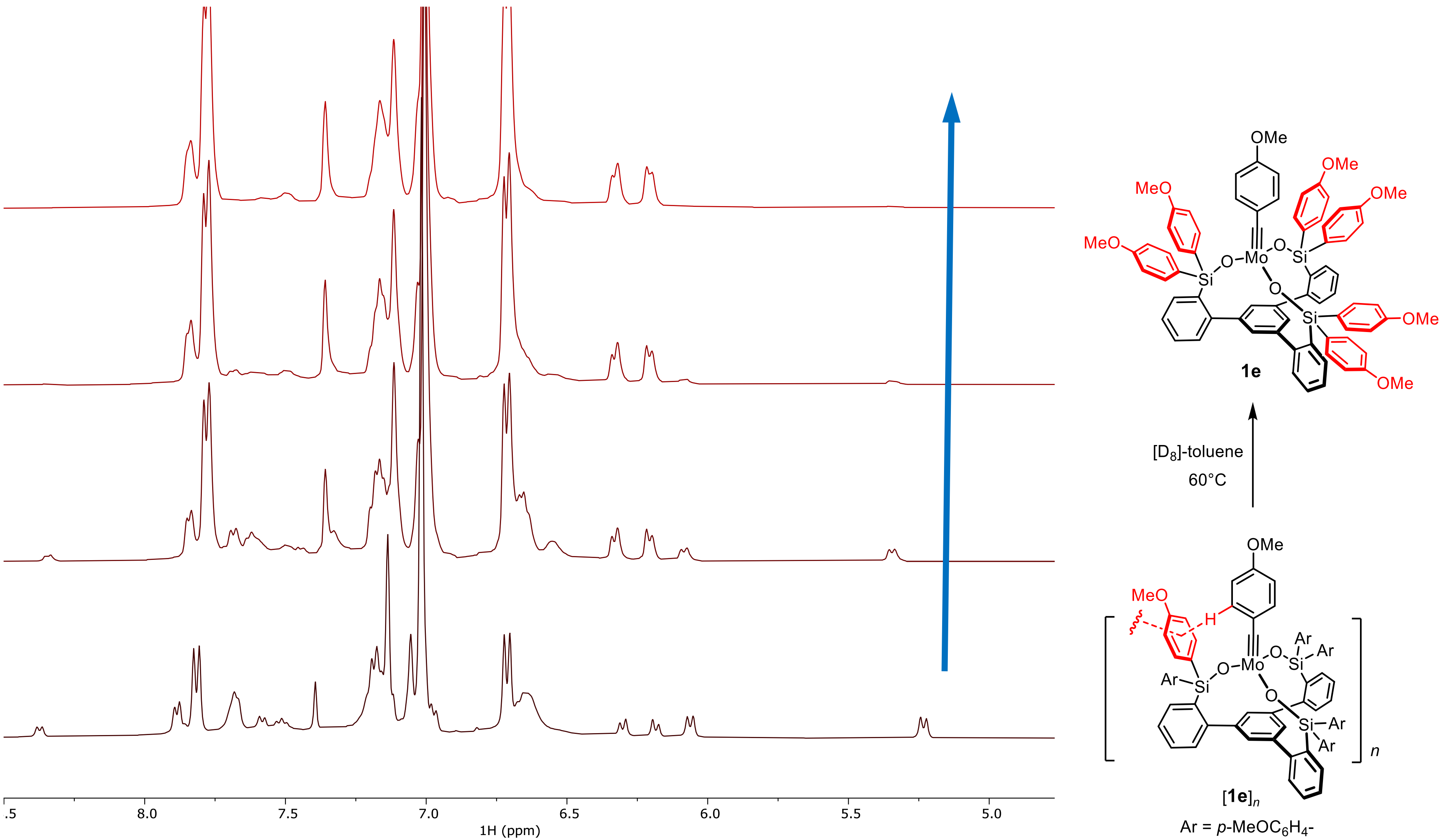

^{1}H NMR study of the conversion of [**1e**]$_n$ to monomeric [**1e**] in [D_8]-toluene at 60°C over the course of 1 h

^{1}H NMR spectrum ([D8]-toluene) of the monomeric complex **1e**

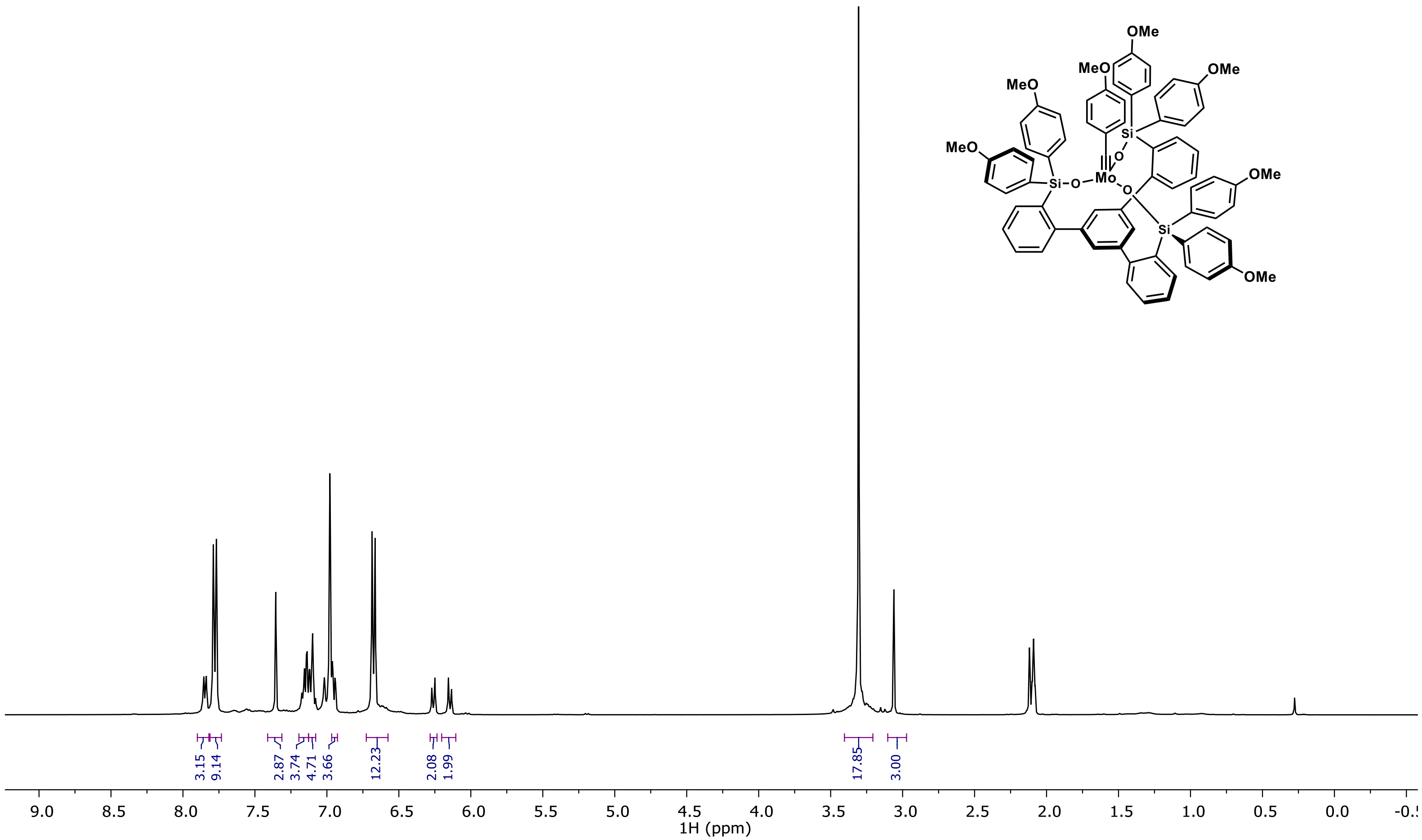

^{13}C NMR spectrum ([D_8]-toluene) of the monomeric complex **1e**

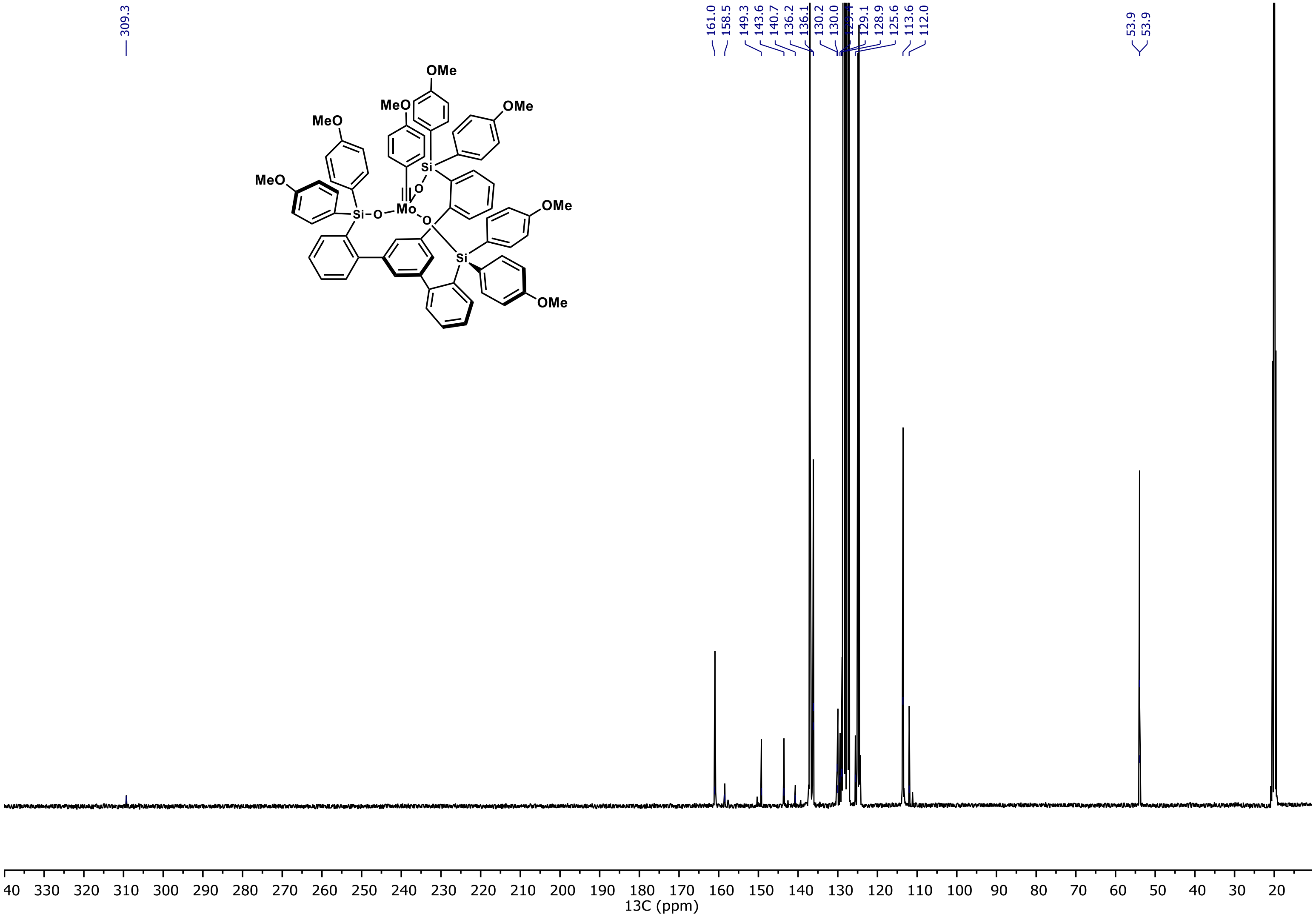

^{29}Si NMR spectrum ([D8]-toluene) of the monomeric complex **1e**

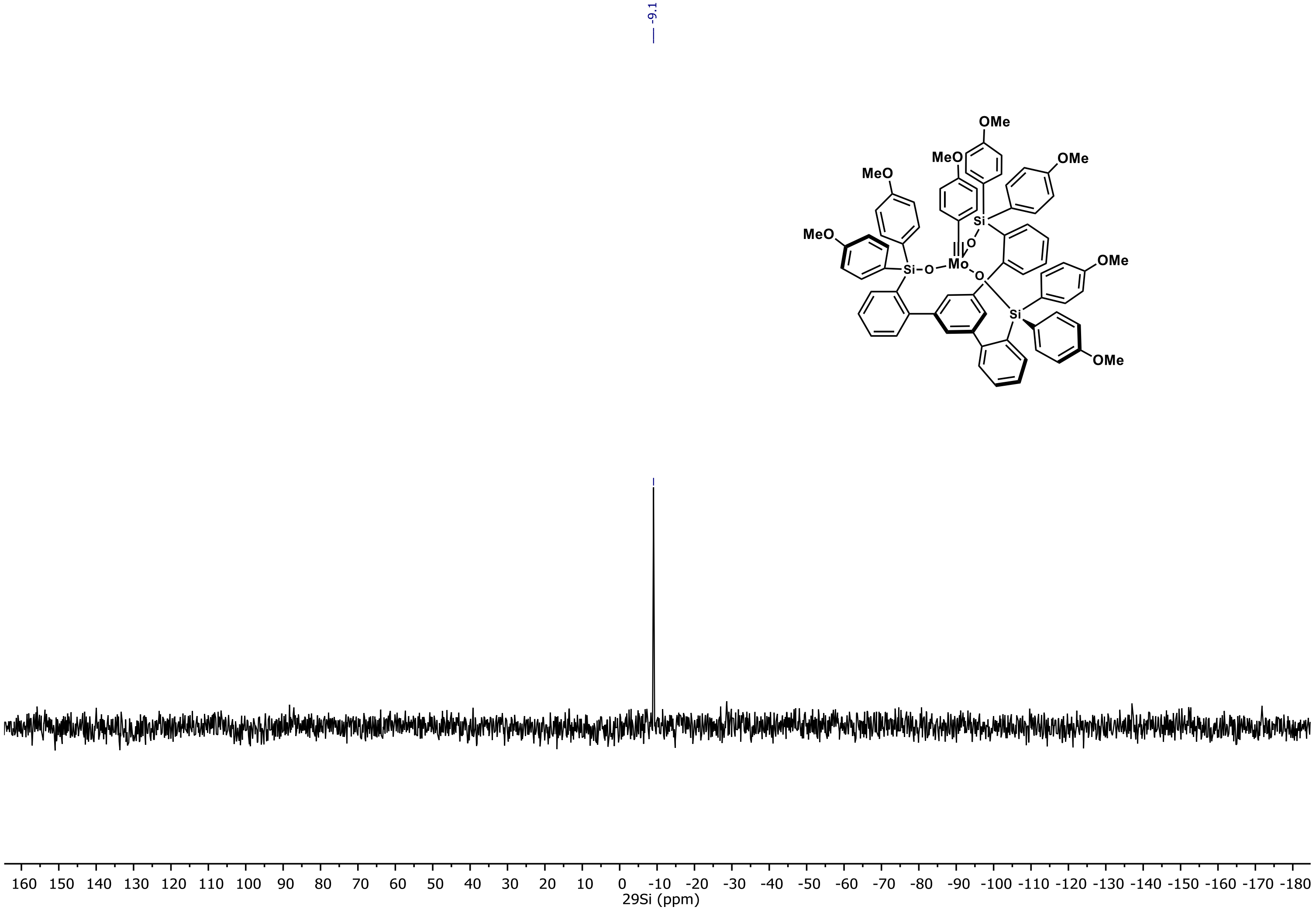

^{95}Mo NMR spectrum ([D$_8$]-toluene, 60°C) of the monomeric complex **1e**

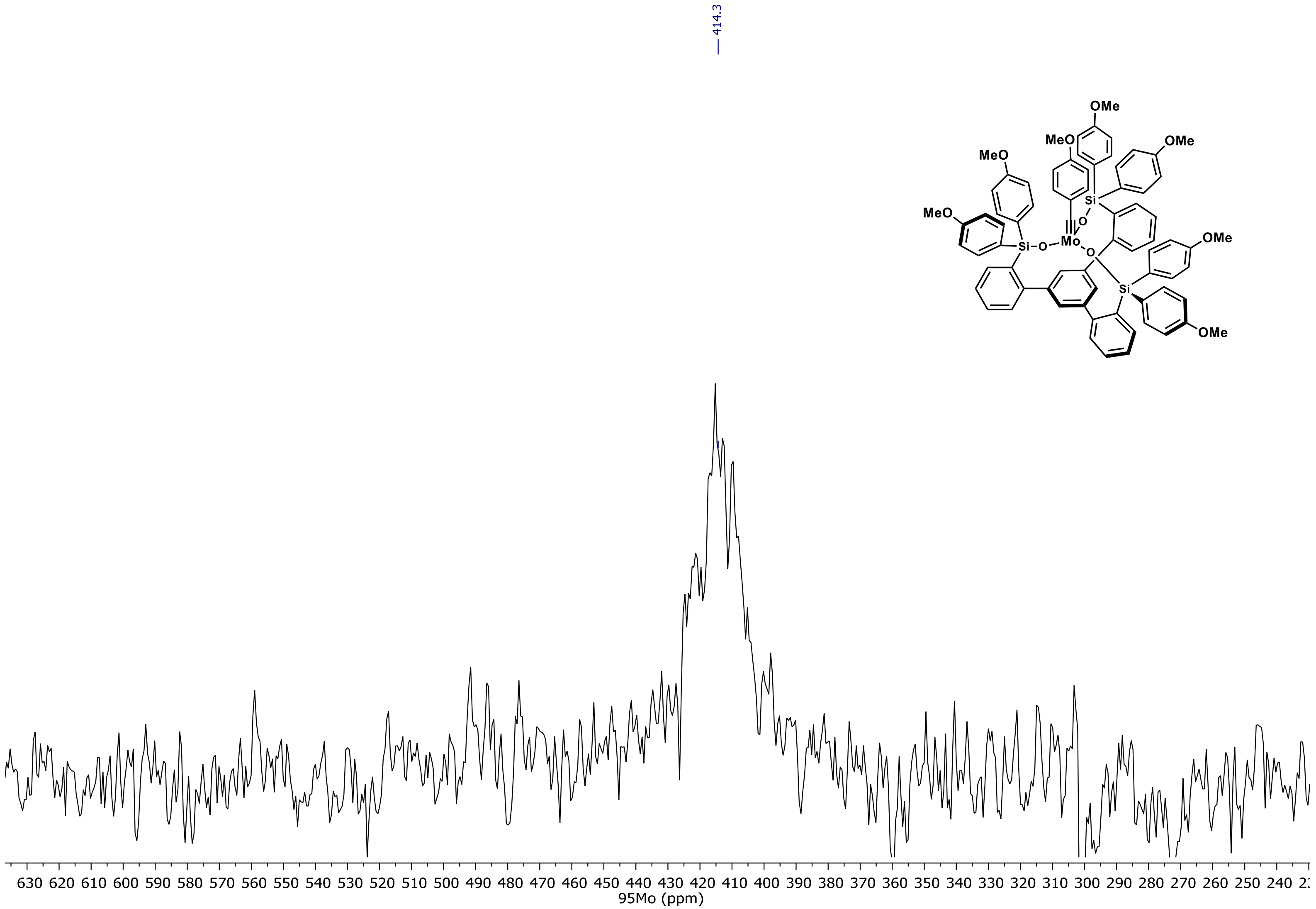

^{1}H NMR spectrum ([D$_8$]-toluene, 25°C) of the tetrameric pyridine adduct **10**

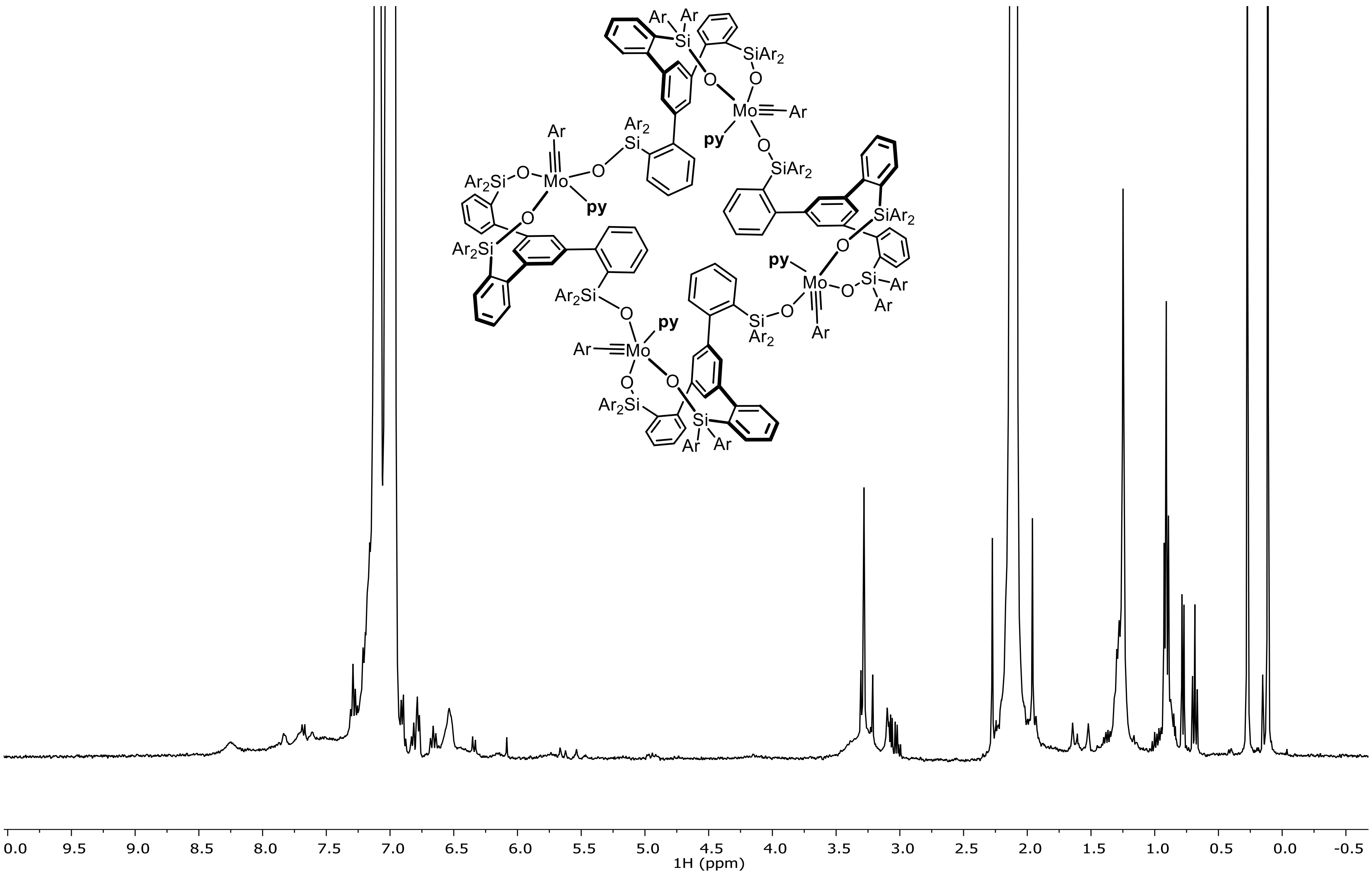

^{1}H NMR spectrum (25°C) of complex [**1e**]$_n$ dissolved in neat [D_5]-pyridine (tetrameric pyridine adduct **10**)

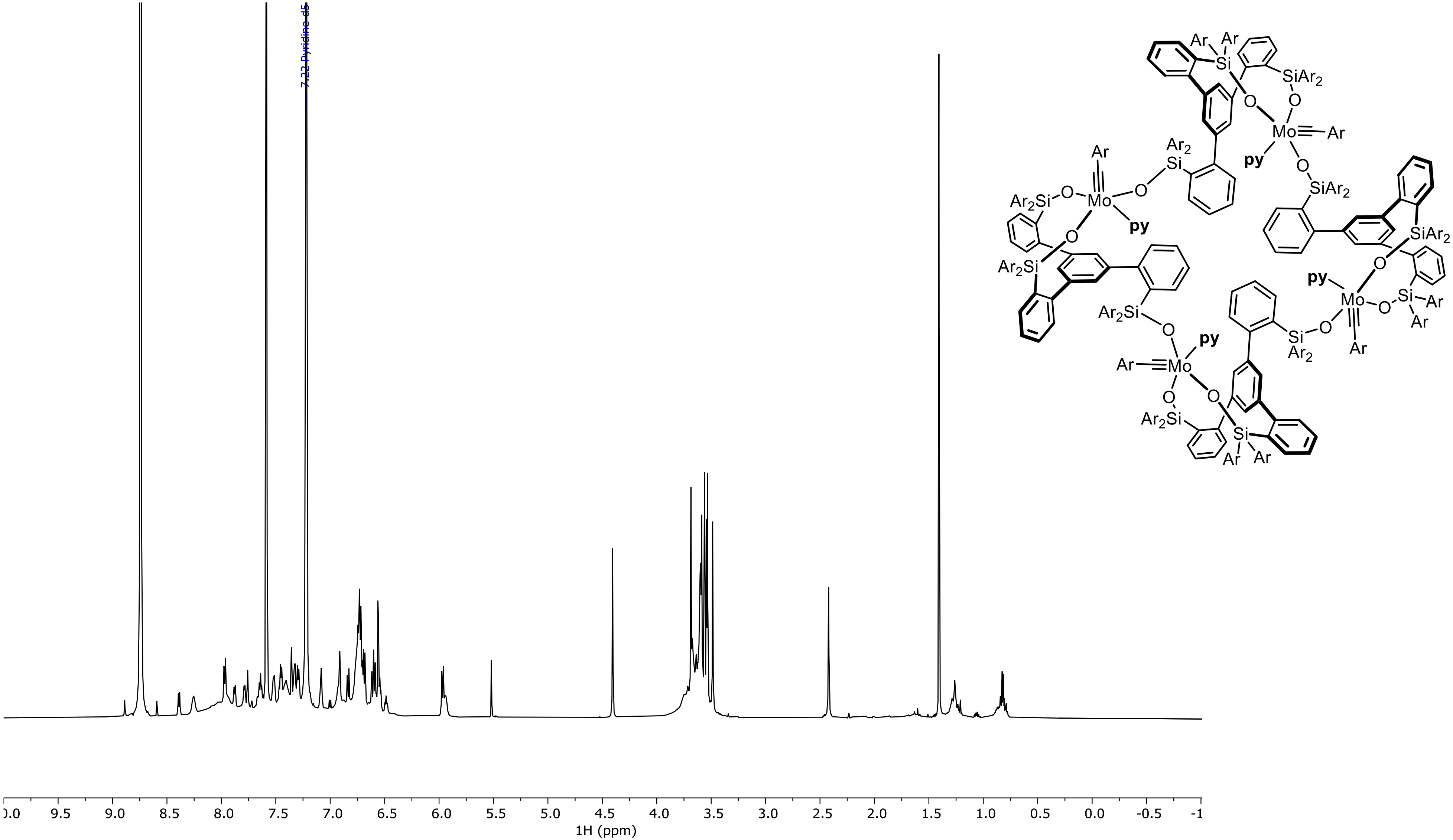

^{1}H NMR of complex **6** recorded at **0.005 mM** concentration (600 MHz, [D$_8$]-toluene, 25ºC)

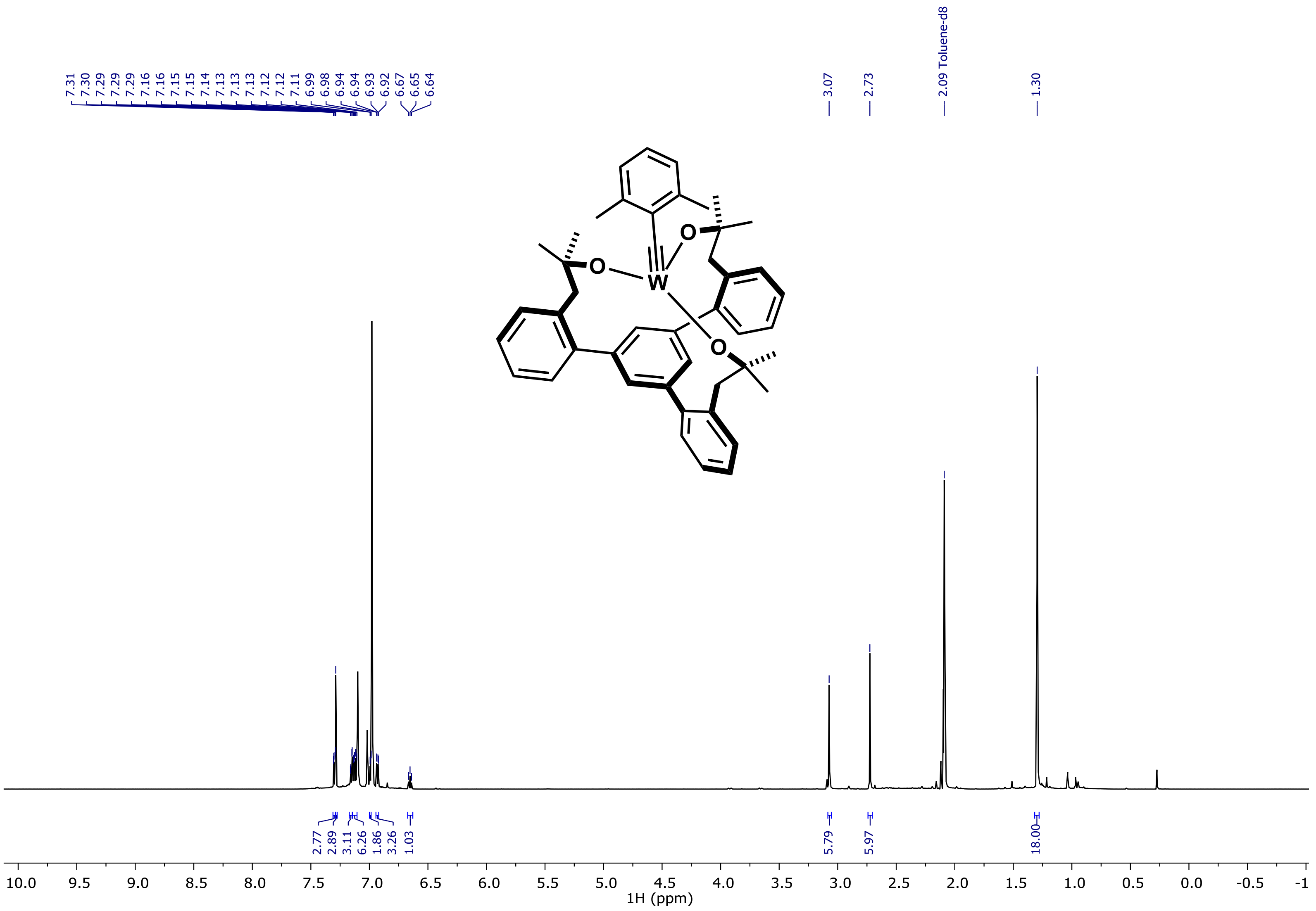

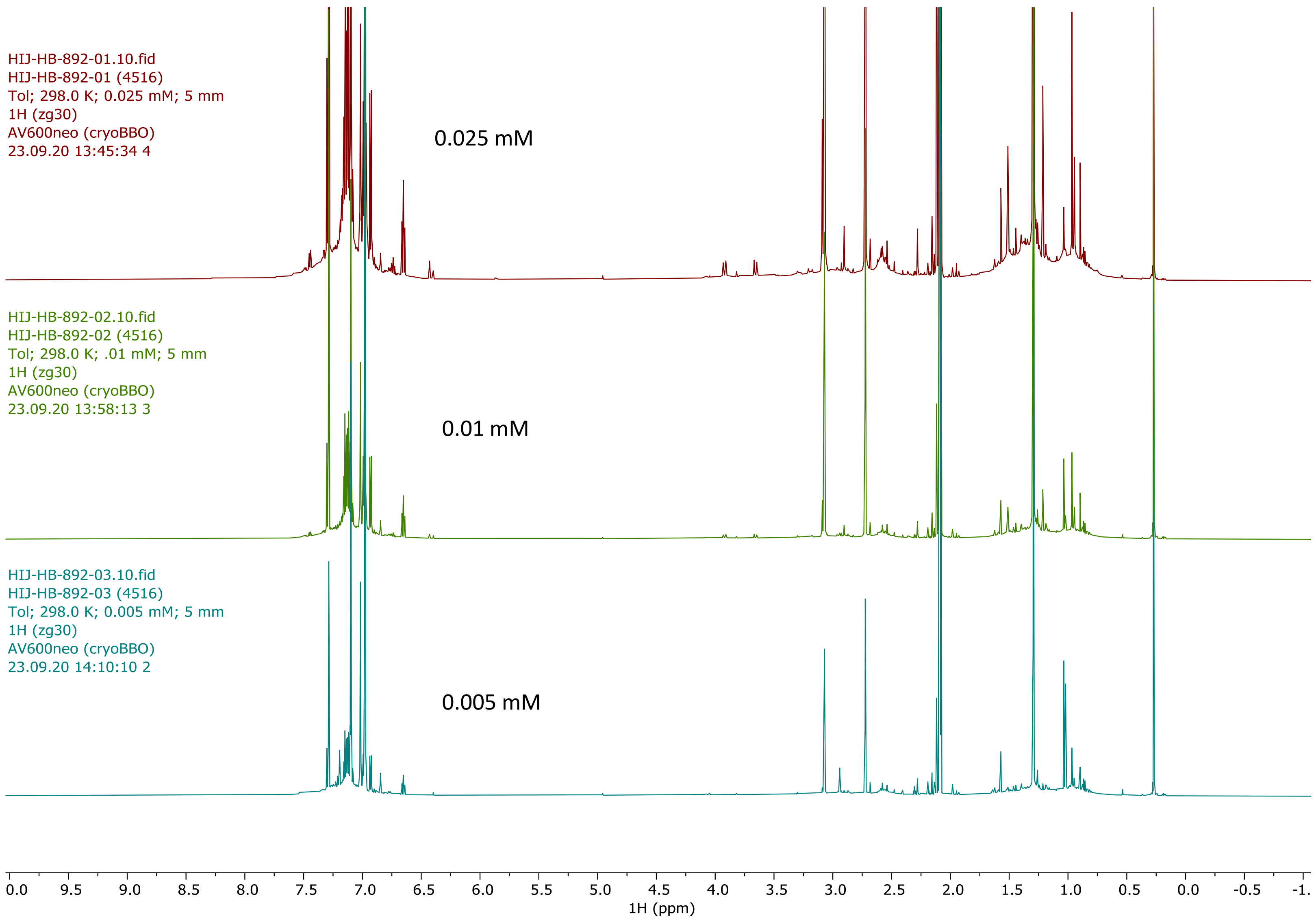

Dilution experiment (0.025 mM, 0.01 mM, 0.005 mM), revealing the concentration-dependent aggregation of the tungsten alkylidynes **6** and **12**

^{1}H NMR of complexes **6** and **12** recorded at **0.025 mM** concentration (600 MHz, [D8]-toluene); insert: diastereotopic benzylic protons

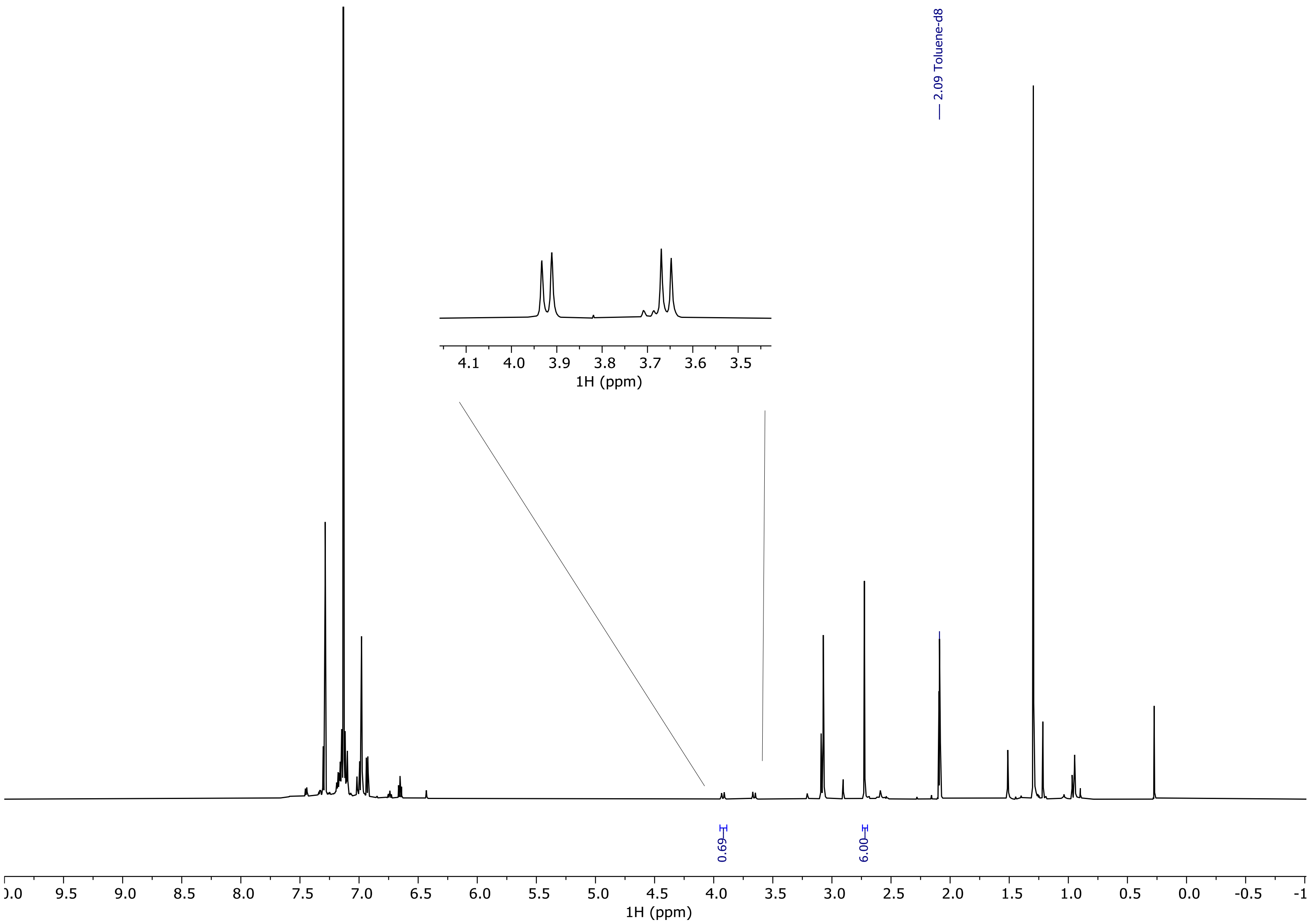

^{13}C NMR of complex **6** recorded at **0.005 mM** concentration (600 MHz, [D$_8$]-toluene, 25ºC)

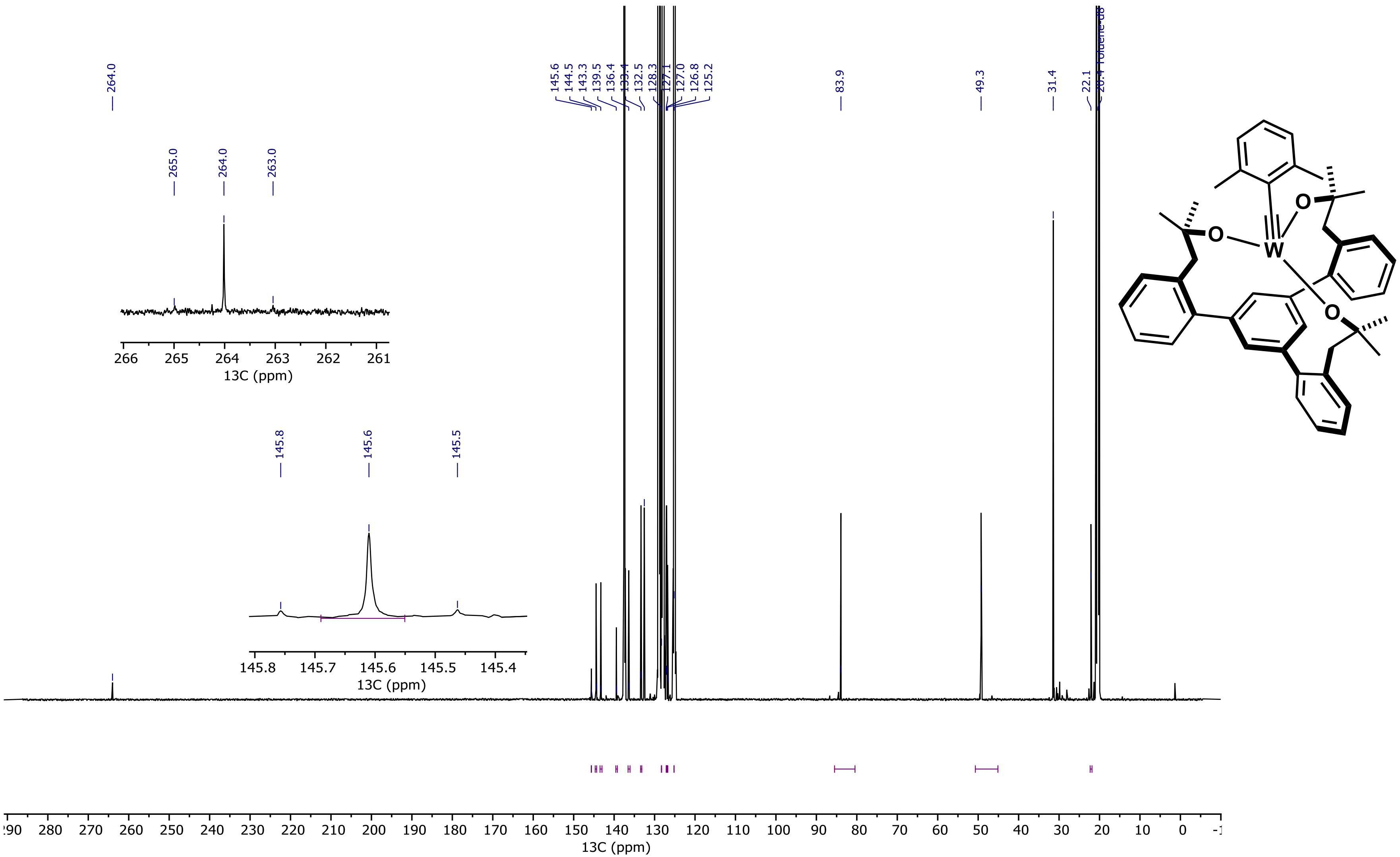

Excerpt of the ^{13}C NMR spectrum recorded at **0.025 mM** concentration ($[D_8]$-toluene), showing the presence of the two distinct tungsten alkylidyne species **6** and **12**

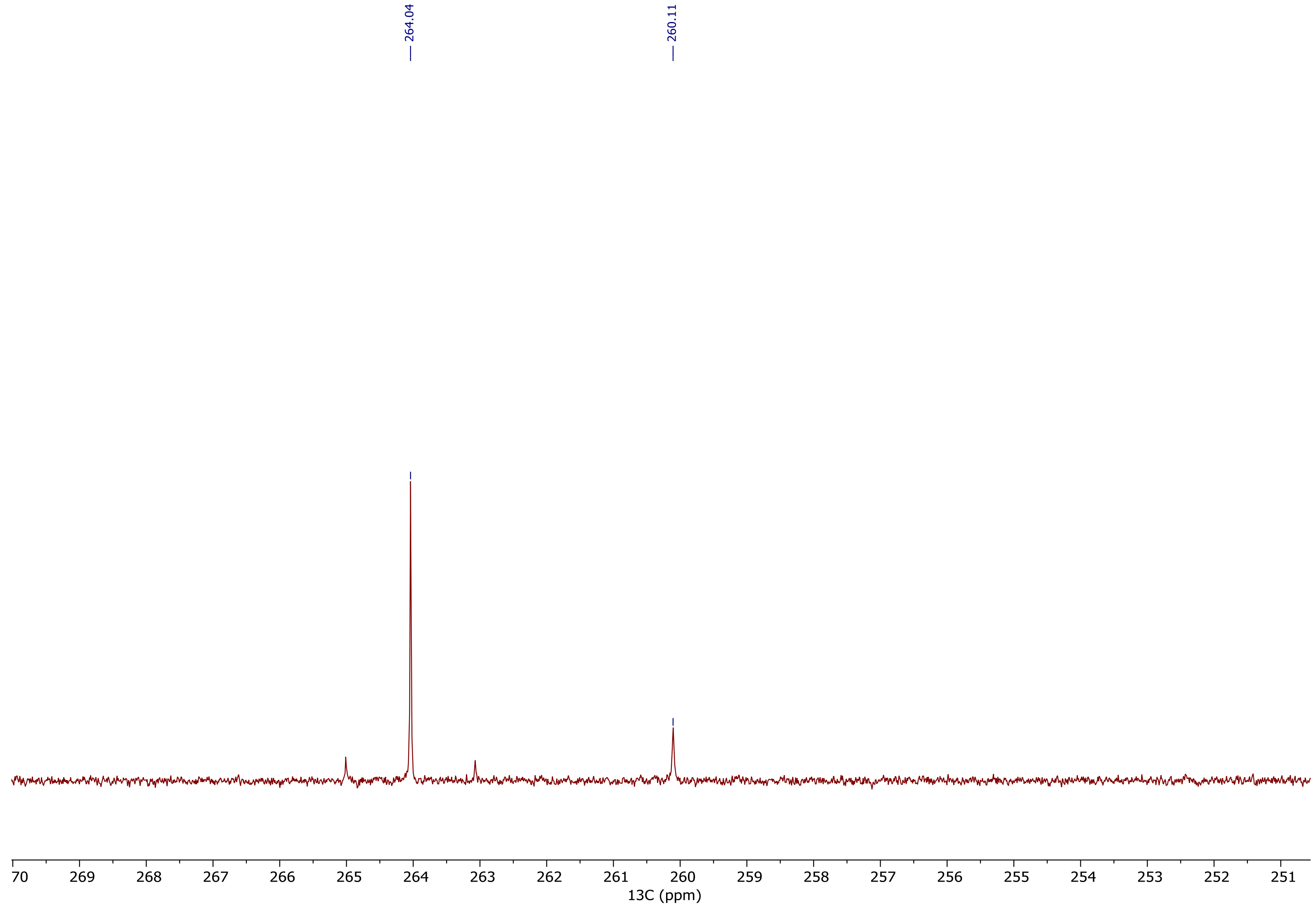

^{183}W NMR projection created from a 2D-HMBC experiment recorded at **0.005 mM** concentration (17 MHz, [D$_8$]-toluene, 25°C), showing the resonance of complex **6**

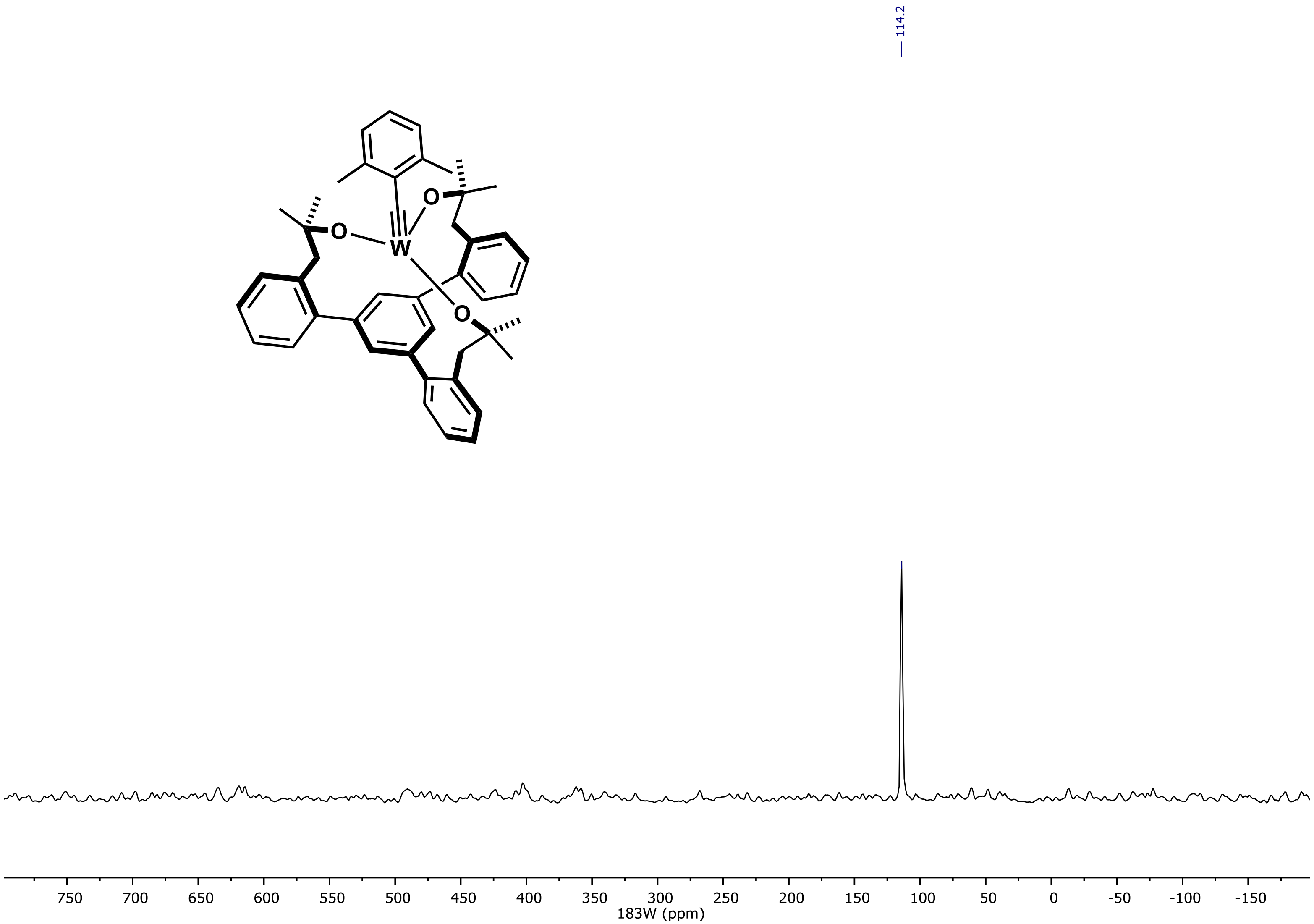

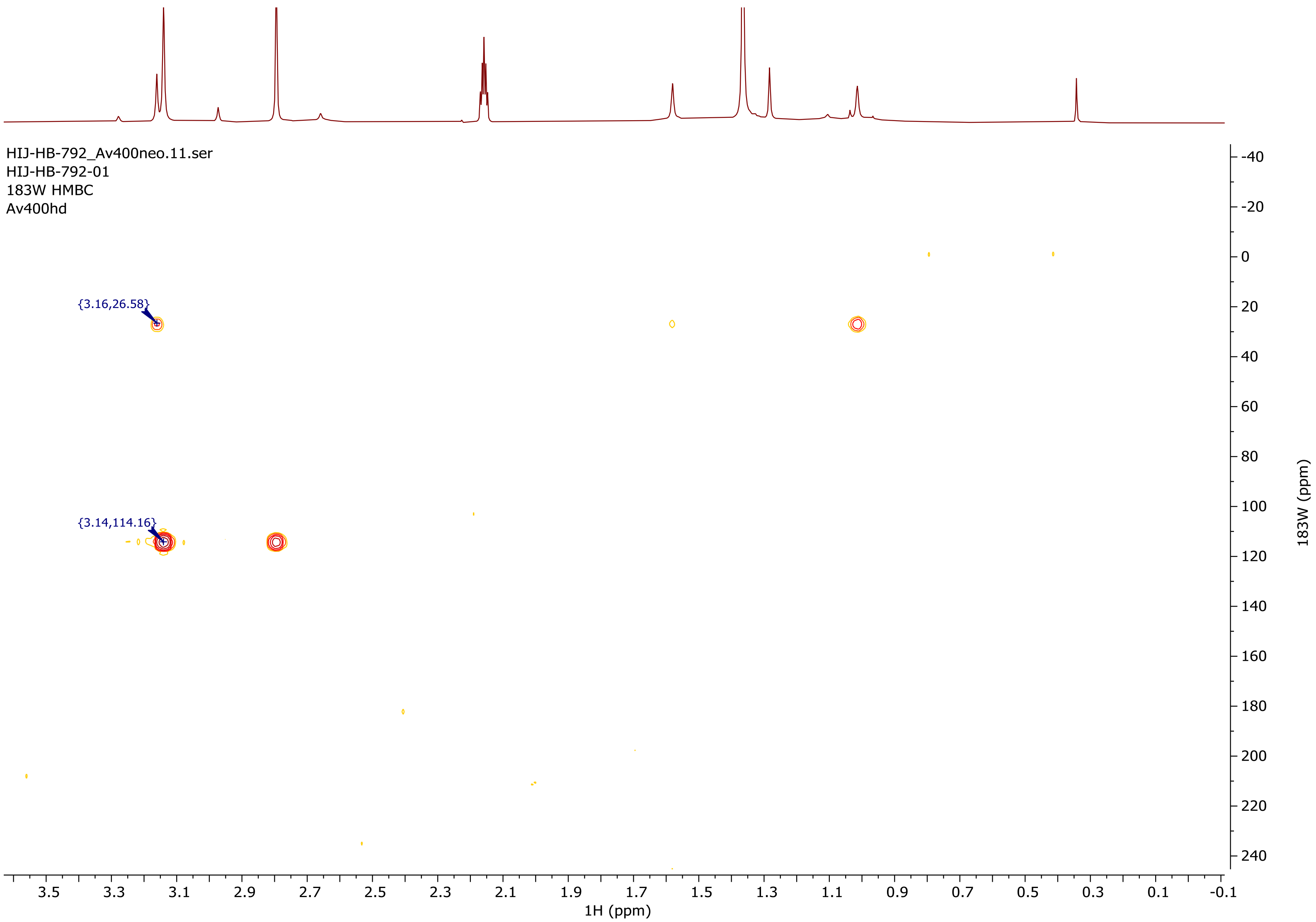

$^{1}H,^{183}W$-HMBC spectrum ([D_8]-toluene) recorded at **0.025 mM** concentration, confirming the presence of two distinct alkylidyne species **6** and **12**

References

1. Harris, R. K.; Becker, E. D.; De Menezes, S. M.; Granger, P.; Hoffman, R. E.; Zilm, K. W., Further conventions for NMR shielding and chemical shifts (IUPAC Recommendations 2008). *Magn. Reson. Chem.* **2008,** *46* (6), 582-98.
2. Evans, R.; Dal Poggetto, G.; Nilsson, M.; Morris, G. A., Improving the Interpretation of Small Molecule Diffusion Coefficients. *Analytical Chemistry* **2018,** *90* (6), 3987-3994.
3. Hillenbrand, J.; Leutzsch, M.; Gordon, C. P.; Copéret, C.; Fürstner, A., 183W NMR Spectroscopy Guides the Search for Tungsten Alkylidyne Catalysts for Alkyne Metathesis. *Angew. Chem. Int. Ed.* **2020,** *59*, 21758-21768.
4. Hillenbrand, J.; Leutzsch, M.; Yiannakas, E.; Gordon, C. P.; Wille, C.; Nöthling, N.; Copéret, C.; Fürstner, A., "Canopy Catalysts" for Alkyne Metathesis: Molybdenum Alkylidyne Complexes with a Tripodal Ligand Framework. *J. Am. Chem. Soc.* **2020,** *142* (25), 11279-11294.
5. Hillenbrand, J.; Leutzsch, M.; Fürstner, A., Molybdenum Alkylidyne Complexes with Tripodal Silanolate Ligands. The Next Generation of Alkyne Metathesis Catalysts. *Angew. Chem. Int. Ed.* **2019,** *58*, 15690-15696.
